# Supplementary material for: The novel anti-CRISPR AcrIIA22 relieves DNA torsion in target plasmids and impairs SpyCas9 activity
Source: PLoS Biol. 2021 Oct 13;19(10):e3001428. doi: 10.1371/journal.pbio.3001428 (PMC8545432; doi:10.1371/journal.pbio.3001428)
Supplement: S1 Fig — Growth rates with orf_1 induction (green) are 7% lower than those without orf_1 induction (orange). The CFU data shown in Fig 1C were generated from the same experiment depicted here (samples were removed after 6 hours of growth to determine these CFU counts). Thus, these data demonstrate that anti-SpyCas9 activity occurs under conditions with mild orf_1 toxicity. Growth curves are shown for samples without SpyCas9 induction to ensure that orf_1 toxicity is not mitigated due to elimination of its plasmid. Points indicate averages from 3 replicates. Standard deviations at each time point are so small that the error bars do not exceed the bounds of the data point. The individual numerical values that underlie the summary data in this figure may be found in S1 Data. CFU, colony-forming unit; SpyCas9, Streptococcus pyogenes Cas9. (PDF) [file pbio.3001428.s001.pdf]

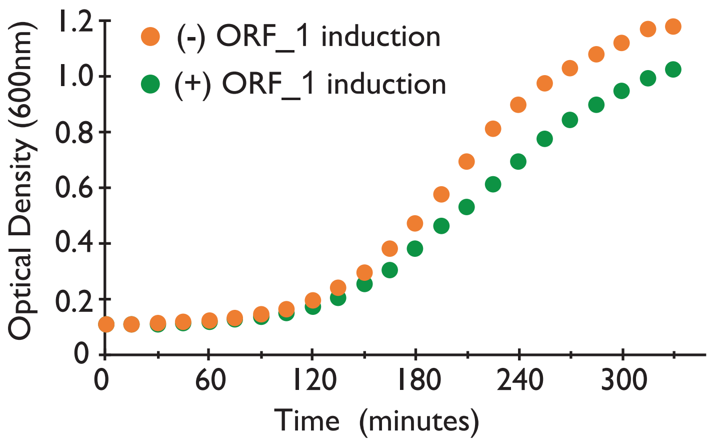

S1 Fig. ***Orf_1* (*acrIIA22*) confers mild toxicity in *E. coli*.** Growth rates with *orf_1* induction (green) are 7% lower than those without *orf_1* induction (orange). The cfu data shown in Fig 1C were generated from the same experiment depicted here (samples were removed after six hours of growth to determine these cfu counts). Thus, these data demonstrate that anti-SpyCas9 activity occurs under conditions with mild *orf_1* toxicity. Growth curves are shown for samples without SpyCas9 induction to ensure that *orf_1* toxicity is not mitigated due to elimination of its plasmid. Points indicate averages from three replicates. Standard deviations at each timepoint are so small that the error bars do not exceed the bounds of the data point. The individual numerical values that underlie the summary data in this figure may be found in S1 Data.
