# Supplementary material for: The novel anti-CRISPR AcrIIA22 relieves DNA torsion in target plasmids and impairs SpyCas9 activity
Source: PLoS Biol. 2021 Oct 13;19(10):e3001428. doi: 10.1371/journal.pbio.3001428 (PMC8545432; doi:10.1371/journal.pbio.3001428)
Supplement: S2 Fig — (A) A schematic description of the experimental design shown in panel (B) is presented. If ORF_1 prevented transcription from pCas9 or altered its copy number, we would expect expression of the orf_1 gene to deplete the level of green fluorescence observed from a construct that replaces the spycas9 gene with gfp. (B) Fluorescence measurements for the experiment depicted in panel A show that ORF_1 does not impact GFP expression throughout an E. coli growth curve. Points indicate averages from 3 replicates; error bars indicate standard deviation. A western blot shows no depletion of SpyCas9 expression as a function of ORF_1 or GFP expression in growing E. coli cultures at 3 hours (C) or 6 hours (D). As an internal control, GAPDH expression was also detected. The individual numerical values and original images for the data presented in this figure may be found in S1 Data and S1 Raw Images, respectively. SpyCas9, Streptococcus pyogenes Cas9. (PDF) [file pbio.3001428.s002.pdf]

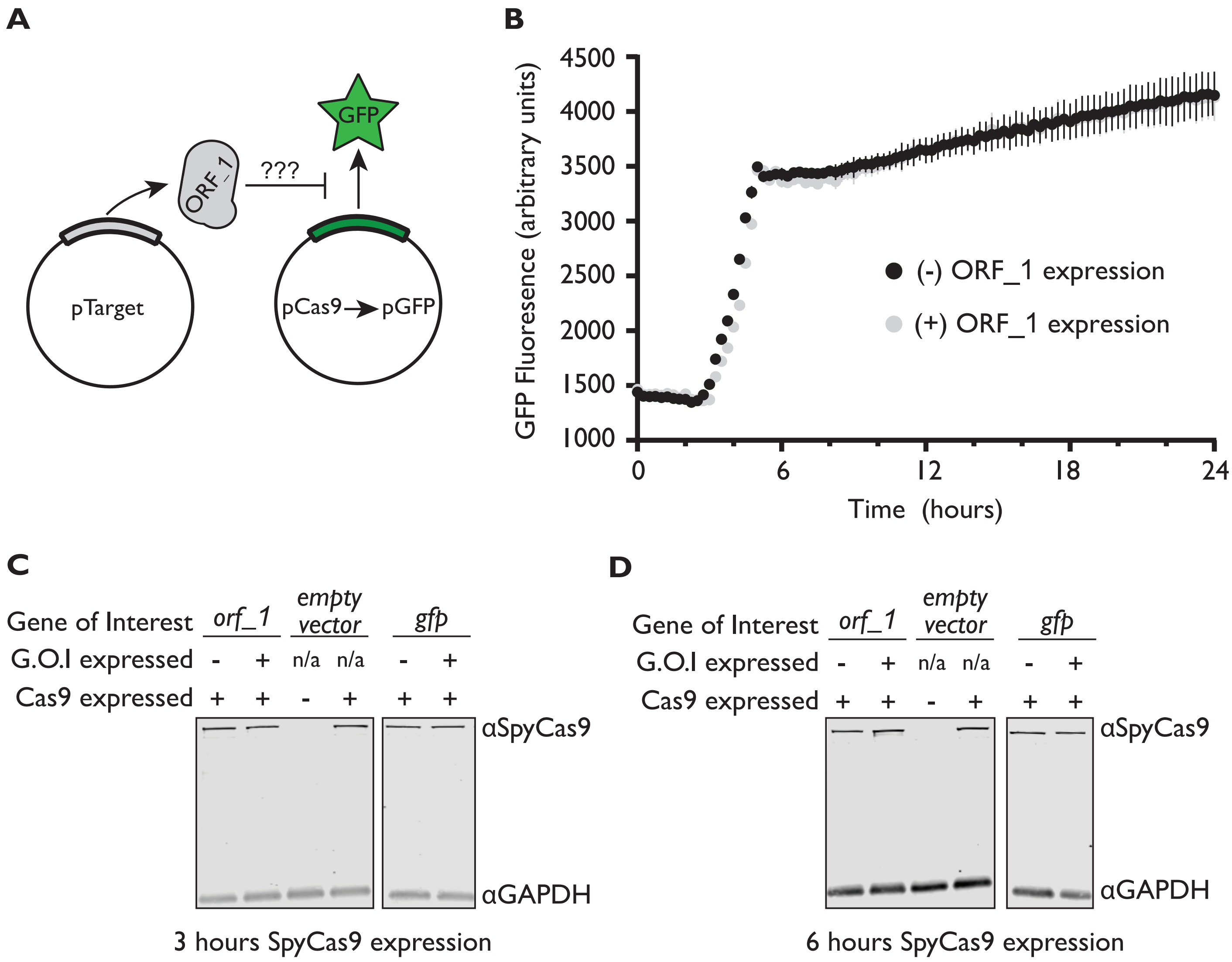

S2 Fig. ***Orf_1 (acrIIA22)* does not impact SpyCas9 expression.** (**A**) A schematic description of the experimental design shown in panel (**B**) is presented. If ORF_1 prevented transcription from pCas9 or altered its copy number, we would expect expression of the *orf_1* gene to deplete the level of green fluorescence observed from a construct that replaces the *spycas9* gene with *gfp*. (**B**) Fluorescence measurements for the experiment depicted in panel A show that ORF_1 does not impact GFP expression throughout an *E. coli* growth curve. Points indicate averages from three replicates, error bars indicate standard deviation. A western blot shows no depletion of SpyCas9 expression as a function of ORF_1 or GFP expression in growing *E. coli* cultures at three hours (**C**) or six hours **(D)**. As an internal control, GAPDH expression was also detected. The individual numerical values and original images for the data presented in this figure may be found in S1 Data and S1_raw_images, respectively.
