# Supplementary material for: The novel anti-CRISPR AcrIIA22 relieves DNA torsion in target plasmids and impairs SpyCas9 activity
Source: PLoS Biol. 2021 Oct 13;19(10):e3001428. doi: 10.1371/journal.pbio.3001428 (PMC8545432; doi:10.1371/journal.pbio.3001428)
Supplement: S3 Fig — Mu phage fitness was measured by plaquing on E. coli in the presence of gfp, acrIIA22, or acrIIA4 via serial 10-fold dilutions. Bacterial clearing (black) occurs when phage Mu overcomes SpyCas9 immunity and lyses E. coli. In (A) and in (B), SpyCas9 with a Mu-targeting crRNA confers substantial protection against phage Mu relative to an n.t. control, in both conditions tested. These conditions are depicted at left, with the only difference being whether SpyCas9 was only expressed in liquid growth prior to phage infection (panel A) or expressed both in liquid media and in solid media throughout infection (panel B). When expressed from a second plasmid, the positive control acrIIA4 significantly enhances Mu fitness by inhibiting SpyCas9 in all conditions in trans. Though acrIIA22 confers protection against SpyCas9 compared to gfp (negative control), this effect is milder than with acrIIA4 and dependent on SpyCas9 expression. crRNA, CRISPR RNA; n.t., nontargeting; SpyCas9, Streptococcus pyogenes Cas9. (PDF) [file pbio.3001428.s003.pdf]

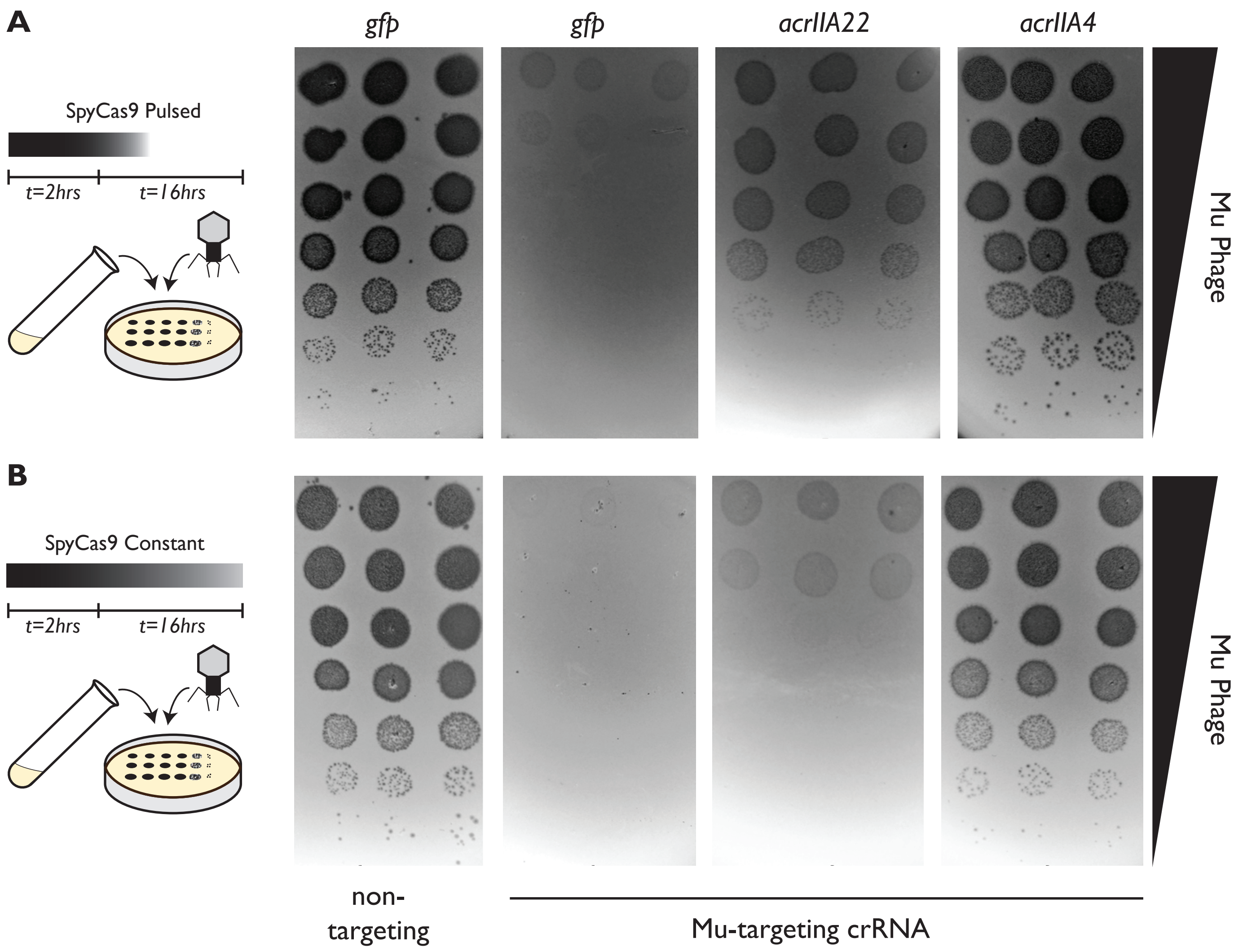

S3 Fig. **AcrIIA22 only modestly protects Mu phages against SpyCas9.** Mu phage fitness was measured by plaquing on *E. coli* in the presence of *gfp*, *acrIIA22*, or *acrIIA4* via serial ten-fold dilutions. Bacterial clearing (black) occurs when phage Mu overcomes SpyCas9 immunity and lyses *E. coli*. In (**A**) and in (**B**), SpyCas9 with a Mu-targeting crRNA confers substantial protection against phage Mu relative to a non-targeting (n.t.) control, in both conditions tested. These conditions are depicted at left, with the only difference being whether SpyCas9 was only expressed in liquid growth prior to phage infection (panel A) or expressed both in liquid media and in solid media throughout infection (panel B). When expressed from a second plasmid, the positive control *acrIIA4* significantly enhances Mu fitness by inhibiting SpyCas9 in all conditions *in trans*. Though *acrIIA22* confers protection against SpyCas9 compared to *gfp* (negative control), this effect is milder than with *acrIIA4* and dependent on SpyCas9 expression.
