# Supplementary material for: The novel anti-CRISPR AcrIIA22 relieves DNA torsion in target plasmids and impairs SpyCas9 activity
Source: PLoS Biol. 2021 Oct 13;19(10):e3001428. doi: 10.1371/journal.pbio.3001428 (PMC8545432; doi:10.1371/journal.pbio.3001428)
Supplement: S4 Fig — (A) Homologs of acrIIA22 are depicted in 3 related prophage genomes, integrated at 3 different genomic loci, revealed by a comparison of prophage-bearing contigs (#57, #56, #37) relative to unintegrated contigs (#55, #58, #17, respectively), which are otherwise nearly identical. Prophage genes are colored by functional category, according to the legend at the left of panel A. Genes immediately adjacent to acrIIA22 (solid boxes) vary across phages, despite strong relatedness across much of the prophage genomes. Bacterial genes are colored gray, except for contig #17, which is also depicted in panel B, below. (B) Homologs of acrIIA22 are depicted in diverse genomic islands, including Contig #1, whose sequence includes a portion that is identical to the original metagenomic contig we recovered (F01A_4). All acrIIA22 homologs in these loci are closely related but differ in their adjacent genes, which often have homologs in the prophages depicted in panel A (dashed boxes). Bacterial genomic regions flanking these hypervariable islands are nearly identical to one another and to prophage integration locus #3, as shown by homology to contig #17 from panel A. Contigs are numbered to indicate their descriptions in S3 Table, which contains their metadata, taxonomy, and sequence retrieval information. All sequences and annotations can also be found in S2 and S3 Data. (C) We tabulate the prevalence of various protein families (clustered at 65% amino acid identity) in a set of 54 unique genomic islands. Each of these islands is flanked by the conserved genes purF and radC but contains a different arrangement of encoded genes. Domain-level annotations are indicated below each protein family. Gene symbols above each protein family are colored and lettered to indicate their counterparts or homologs in panels A and B. The phage capsid icon indicates sequences with homologs in prophage genomes. (D) An evolutionary model for the origin of the acrIIA22-encoding hypervariable genomic isl [file pbio.3001428.s004.pdf]

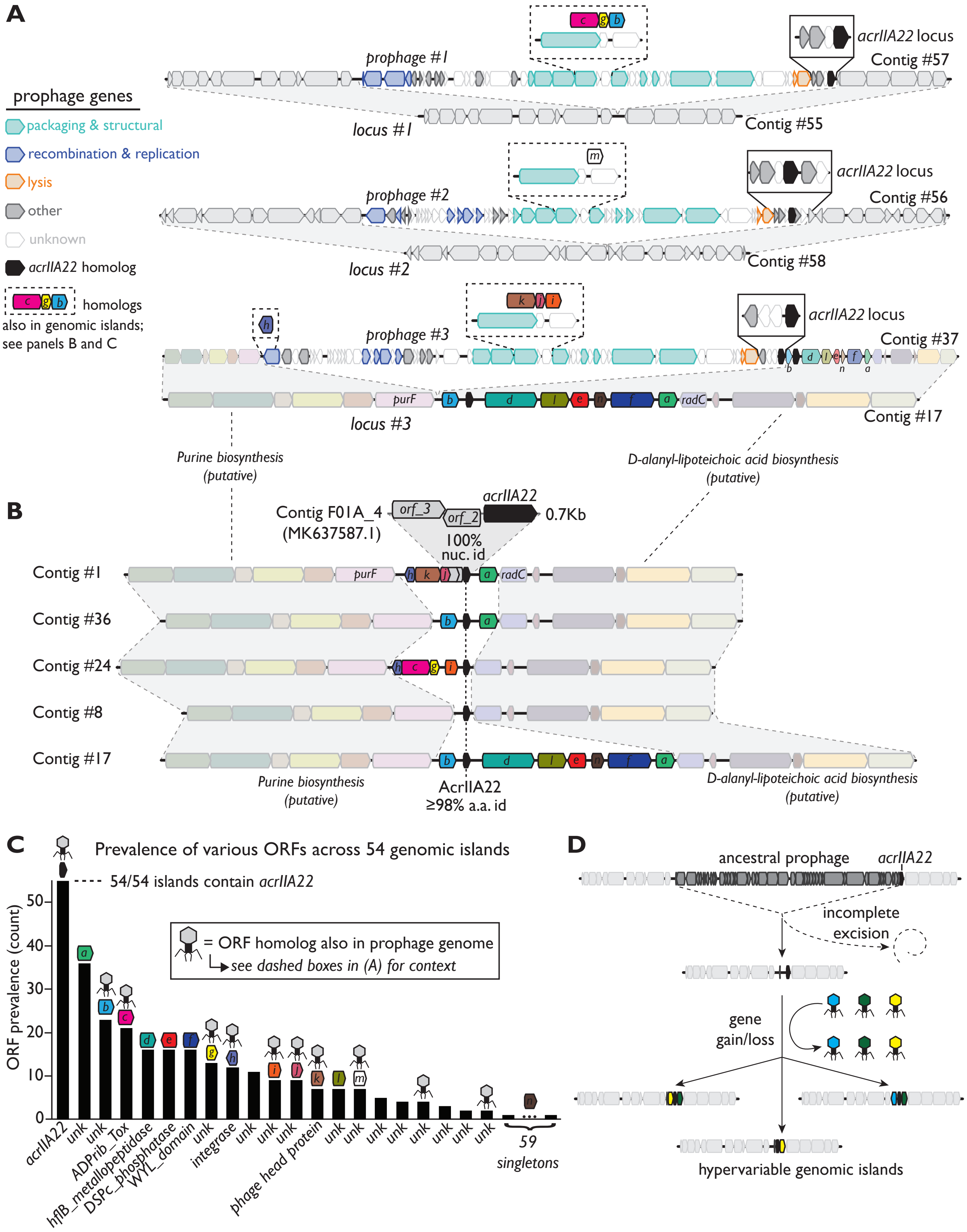
A
prophage genes
packaging & structural
recombination & replication
lysis
other
unknown
acrIIA22 homolog
homologs also in genomic islands; see panels B and C
prophage #1
acrIIA22 locus
Contig #57
Contig #55
locus #1
prophage #2
Contig #56
Contig #58
locus #2
prophage #3
Contig #37
purF
radC
Contig #17
locus #3
Purine biosynthesis (putative)
D-alanyl-lipoteichoic acid biosynthesis (putative)
B
Contig F01A_4 (MK637587.1)
orf_3
orf_2
acrIIA22
0.7Kb
100% nuc. id
Contig #1
Contig #36
Contig #24
Contig #8
Contig #17
AcrIIA22 ≥98% a.a. id
C
Prevalence of various ORFs across 54 genomic islands
54/54 islands contain acrIIA22
= ORF homolog also in prophage genome
see dashed boxes in (A) for context
ORF prevalence (count)
acrIIA22
unk
ADPrib_Tox
hflB_metallopeptidase
DSPc_phosphatase
WYL_domain
integrase
phage head protein
59 singletons
D
ancestral prophage
incomplete excision
gene gain/loss
hypervariable genomic islands

S4 Fig.

S4 Fig. ***AcrIIA22* homologs are found in hypervariable regions of prophage and bacterial genomes in the CAG-217clostridial genus.** (**A**) Homologs of *acrIIA22* are depicted in three related prophage genomes, integrated at three different genomic loci, revealed by a comparison of prophage-bearing contigs (#57, #56, #37) relative to unintegrated contigs (#55, #58, #17 respectively), which are otherwise nearly identical. Prophage genes are colored by functional category, according to the legend at the left of panel A. Genes immediately adjacent to *acrIIA22* (solid boxes) vary across phages, despite strong relatedness across much of the prophage genomes. Bacterial genes are colored gray, except for contig #17, which is also depicted in panel B, below. (**B**) Homologs of *acrIIA22* are depicted in diverse genomic islands, including Contig #1, whose sequence includes a portion that is identical to the original metagenomic contig we recovered (F01A_4). All *acrIIA22* homologs in these loci are closely related but differ in their adjacent genes, which often have homologs in the prophages depicted in panel A (dashed boxes). Bacterial genomic regions flanking these hypervariable islands are nearly identical to one another and to prophage integration locus #3, as shown by homology to contig #17 from panel A. Contigs are numbered to indicate their descriptions in S3 Table, which contains their metadata, taxonomy, and sequence retrieval information. All sequences and annotations can also be found in S2 Data and S3 Data. (**C**) We tabulate the prevalence of various protein families (clustered at 65% amino acid identity) in a set of 54 unique genomic islands. Each of these islands is flanked by the conserved genes *purF* and *radC* but contains a different arrangement of encoded genes. Domain-level annotations are indicated below each protein family (unk; unknown function). Gene symbols above each protein family are colored and lettered to indicate their counterparts or homologs in panels A and B. The phage capsid icon indicates sequences with homologs in prophage genomes. (**D**) An evolutionary model for the origin of the *acrIIA22*-encoding hypervariable genomic islands depicted in panel B is shown. This panel is reprinted from Fig 2C, for continuity. We propose that *acrIIA22* moved via a phage insertion into a bacterial genomic locus, remained following an incomplete prophage excision event, and its neighboring genes subsequently diversified via horizontal exchange with additional phage genomes. The individual numerical values that underlie the summary data in this figure may be found in S1 Data.
