# Supplementary material for: The novel anti-CRISPR AcrIIA22 relieves DNA torsion in target plasmids and impairs SpyCas9 activity
Source: PLoS Biol. 2021 Oct 13;19(10):e3001428. doi: 10.1371/journal.pbio.3001428 (PMC8545432; doi:10.1371/journal.pbio.3001428)
Supplement: S5 Fig — An acrIIA22-encoding prophage like the one depicted in Fig 2A and those in S4A Fig is shown. This prophage encodes for a homolog of the previously described SpyCas9 inhibitor acrIIA17 within 1 kilobase of an acrIIA22 homolog. Sequence relatedness between the depicted acrIIA17 gene and the originally discovered acrIIA17 is shown [22]. Because phages often encode multiple acrs in the same locus, the colocalization of acrIIA17 with acrIIA22 is consistent with the latter gene functioning natively to inhibit CRISPR-Cas activity. Prophage genes are colored by functional category, per the legend and as in S4A Fig. Contigs are numbered to indicate their descriptions in S3 Table, which contains their metadata, taxonomy, and sequence retrieval information. All sequences and annotations can also be found in S2 and S3 Data. Acr, anti-CRISPR; SpyCas9, Streptococcus pyogenes Cas9. (PDF) [file pbio.3001428.s005.pdf]

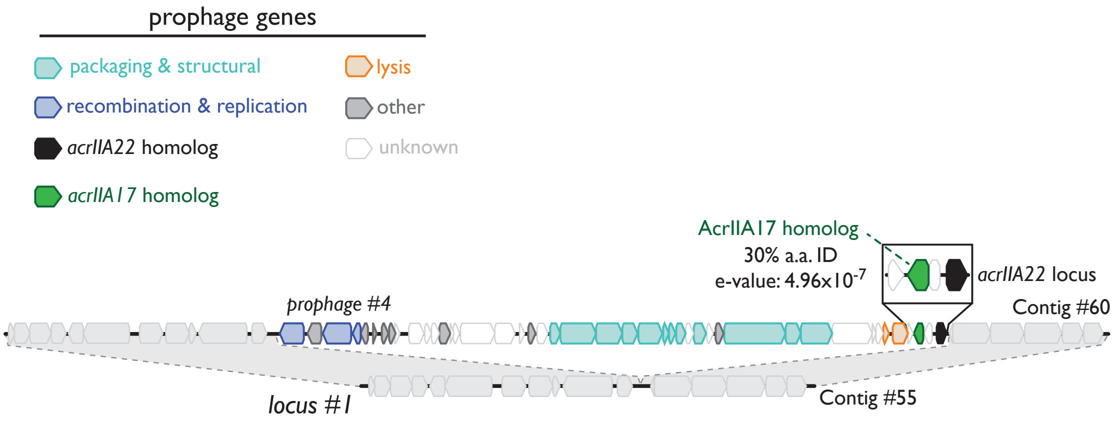

S5 Fig. **Genomic proximity of *acrIIA22* homologs to other *acr* genes.** An *acrIIA22*-encoding prophage like the one depicted in Fig 2A and those in S4A Fig is shown. This prophage encodes for a homolog of the previously described SpyCas9 inhibitor *acrIIA17* within one kilobase of an *acrIIA22* homolog. Sequence relatedness between the depicted *acrIIA17* gene and the originally discovered acrIIA17 is shown[22]. Because phages often encode multiple *acr*s in the same locus, the co-localization of *acrIIA17* with *acrIIA22* is consistent with the latter gene functioning natively to inhibit CRISPR-Cas activity. Prophage genes are colored by functional category, per the legend and as in S4A Fig. Contigs are numbered to indicate their descriptions in S3 Table, which contains their metadata, taxonomy, and sequence retrieval information. All sequences and annotations can also be found in S2 Data and S3 Data.
