# Supplementary material for: The novel anti-CRISPR AcrIIA22 relieves DNA torsion in target plasmids and impairs SpyCas9 activity
Source: PLoS Biol. 2021 Oct 13;19(10):e3001428. doi: 10.1371/journal.pbio.3001428 (PMC8545432; doi:10.1371/journal.pbio.3001428)
Supplement: S6 Fig — SpyCas9 and sgRNA were preincubated before mixing with a TS-tagged AcrIIA22 or AcrIIA4. SpyCas9 without sgRNA was also used. Strep-Tactin pulldowns on AcrIIA4 also pulled down SpyCas9 preincubated with sgRNA, as previously reported [12]. Similar pulldowns with AcrIIA22 indicate little to no interaction with SpyCas9, regardless of whether sgRNA was used. These images depict total protein content visualized by Coomassie stain. Reaction components are indicated below the gel image. Asterisks (*) and dagger (†) symbols indicate AcrIIA4 and AcrIIA22 protein bands that run at slightly different positions than expected due to gel distortion. Original, uncropped versions of images depicted in figure may be found in the Supporting information file, S1 Raw Images. sgRNA, single-guide RNA; SpyCas9, Streptococcus pyogenes Cas9; TS, twin-strep. (PDF) [file pbio.3001428.s006.pdf]

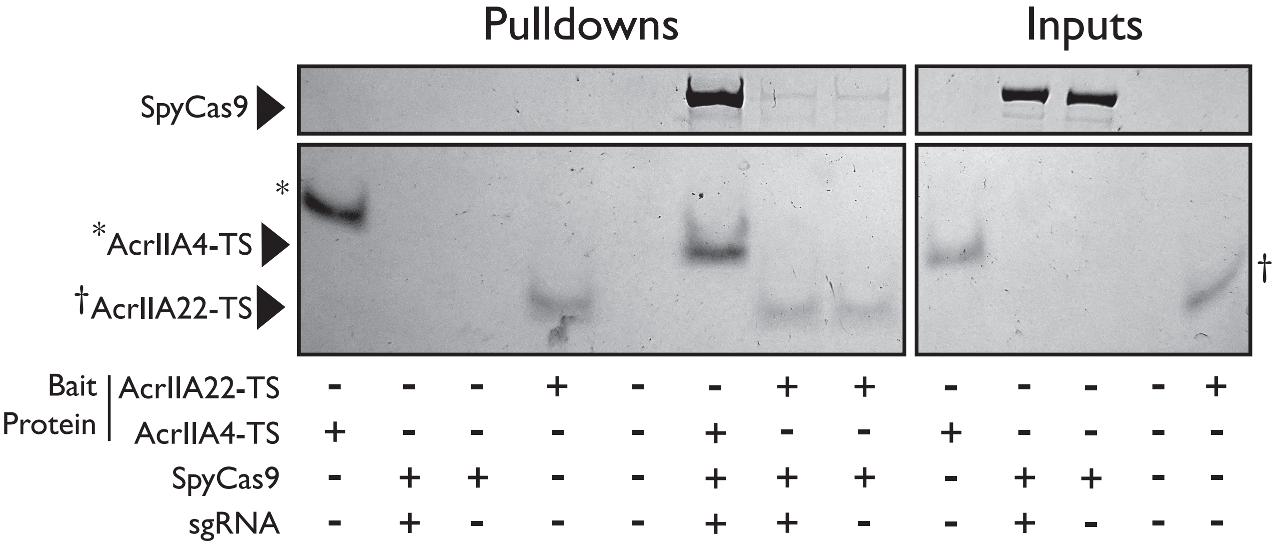

S6 Fig. **AcrIIA22 does not strongly bind SpyCas9.** SpyCas9 and sgRNA were pre-incubated before mixing with a twin-strep (TS) tagged AcrIIA22 or AcrIIA4. SpyCas9 without sgRNA was also used. Strep-Tactin pulldowns on AcrIIA4 also pulled down SpyCas9 pre-incubated with sgRNA, as previously reported[12]. Similar pulldowns with AcrIIA22 indicate little to no interaction with SpyCas9, regardless of whether sgRNA was used. These images depict total protein content visualized by Coomassie stain. Reaction components are indicated below the gel image. Asterisks (*) and dagger (†) symbols indicate AcrIIA4 and AcrIIA22 protein bands that run at slightly different positions than expected due to gel distortion. Original, uncropped versions of images depicted in figure may be found in the supporting information file, S1_raw_images.
