# Supplementary material for: The novel anti-CRISPR AcrIIA22 relieves DNA torsion in target plasmids and impairs SpyCas9 activity
Source: PLoS Biol. 2021 Oct 13;19(10):e3001428. doi: 10.1371/journal.pbio.3001428 (PMC8545432; doi:10.1371/journal.pbio.3001428)
Supplement: S7 Fig — (A) A schematic cartoon depicts the experiment in panel (B). SpyCas9 was preincubated with sgRNA targeting linear DNA. Then, Acr candidates were added. Subsequently, cleavage reactions were performed, and the DNA products visualized by gel electrophoresis. (B) We show the products of the reactions described in panel A for the inhibitors AcrIIA22 and AcrIIA4. SpyCas9 activity is greatly inhibited by AcrIIA4 but unaffected by AcrIIA22, as indicated by the proportion of cleaved DNA product. Reaction components are depicted atop the gel image, with molar equivalents relative to SpyCas9 indicated. The percent of DNA substrate cleaved by SpyCas9 is quantified below each lane. (C) We perform a similar experiment as in panel A, except candidate Acrs were incubated with SpyCas9 before sgRNA addition. Reactions were begun via the simultaneous addition of sgRNA and linear dsDNA instead of just dsDNA. (D) The products of the reactions described in panel C for AcrIIA22 and AcrIIA4 inhibitors are shown. SpyCas9 activity is inhibited by AcrIIA4 but unaffected by AcrIIA22, as indicated by the proportion of cleaved DNA product. The data depicted in this figure are not directly comparable to those in Fig 7, due to methodological differences and because the preparations of SpyCas9 used in each experiment exhibited different activities. Original, uncropped versions of images depicted in figure may be found in the supporting information file, S1 Raw Images. Acr, anti-CRISPR; dsDNA, double-stranded DNA; SpyCas9, Streptococcus pyogenes Cas9; sgRNA, single-guide RNA. (PDF) [file pbio.3001428.s007.pdf]

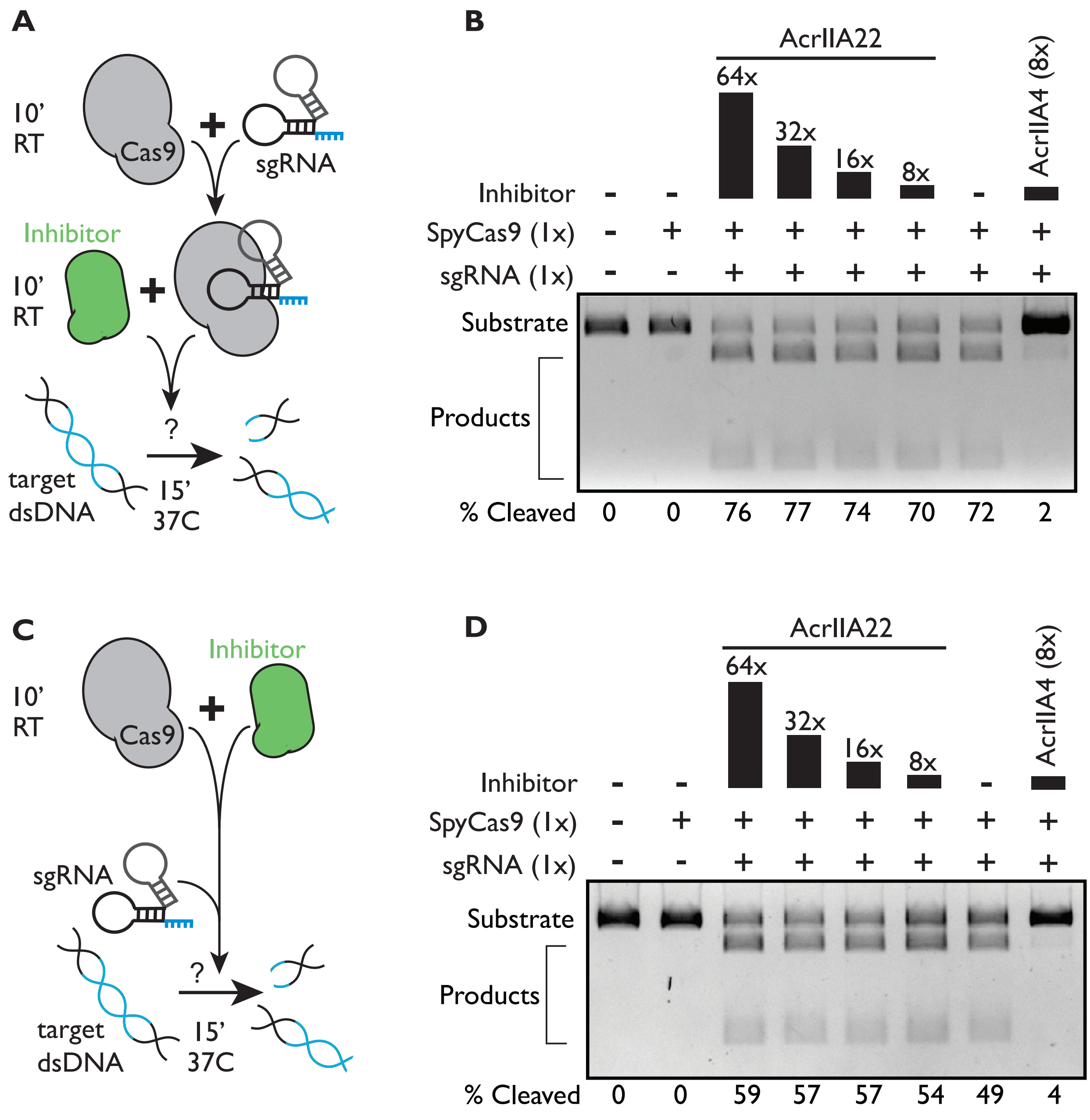

S7 Fig. AcrIIA22 does not protect linear DNA from SpyCas9 cleavage. (**A**) A schematic cartoon depicts the experiment in panel (B). SpyCas9 was pre-incubated with sgRNA targeting linear DNA. Then, Acr candidates were added. Subsequently, cleavage reactions were performed, and the DNA products visualized by gel electrophoresis. (**B**) We show the products of the reactions described in panel A for the inhibitors AcrIIA22 and AcrIIA4. SpyCas9 activity is greatly inhibited by AcrIIA4 but unaffected by AcrIIA22, as indicated by the proportion of cleaved DNA product. Reaction components are depicted atop the gel image, with molar equivalents relative to SpyCas9 indicated. The percent of DNA substrate cleaved by SpyCas9 is quantified below each lane. (**C**) We perform a similar experiment as in panel A, except candidate Acrs were incubated with SpyCas9 before sgRNA addition. Reactions were begun via the simultaneous addition of sgRNA and linear dsDNA instead of just dsDNA. (**D**) The products of the reactions described in panel C for AcrIIA22 and AcrIIA4 inhibitors are shown. SpyCas9 activity is inhibited by AcrIIA4 but unaffected by AcrIIA22, as indicated by the proportion of cleaved DNA product. The data depicted in this figure are not directly comparable to those in Fig 7, due to methodological differences and because the preparations of SpyCas9 used in each experiment exhibited different activities. Original, uncropped versions of images depicted in figure may be found in the supporting information file, S1_raw_images.
