# Supplementary material for: The novel anti-CRISPR AcrIIA22 relieves DNA torsion in target plasmids and impairs SpyCas9 activity
Source: PLoS Biol. 2021 Oct 13;19(10):e3001428. doi: 10.1371/journal.pbio.3001428 (PMC8545432; doi:10.1371/journal.pbio.3001428)
Supplement: S8 Fig — (A) We present a ribbon diagram of a proposed AcrIIA22 tetramer, which requires binding between antiparallel β-strands at the C-termini of AcrIIA22 monomers to form extended, concave β-sheets. This putative oligomerization interface is indicated by the regions highlighted in yellow. Each monomer in the proposed tetramer is labeled with lowercase Roman numerals (i-iv). (B) Space filling model of the tetrameric AcrIIA22 structure from panel A, with relative charge depicted, highlighting a groove (dashed line with arrowhead) that may accommodate nucleic acids (based on analogy to other PC4-like proteins). (C) AcrIIA22 monomers (i) and (ii) from the tetramer in panel A likely interact via a series of hydrophobic interactions, as indicated by the predominantly nonpolar sidechains colored in yellow. The boxed region highlights residue D14, which is important for nicking activity and plasmid protection against SpyCas9, and is enlarged in panel F. (D) In conventional PC4-like family proteins, such as the putative single-stranded DNA binding protein from phage T5 depicted in gray (PDB:4BG7), the same topology of outward facing, concave β-sheets are instead stabilized via interactions between opposing α-helices (depicted in opaque light blue). (E) An overlay of β-sheets from AcrIIA22 (blue, PDB:7JTA) and the phage T5 PC4-like protein (gray, PDB:4BG7) illustrates their similar topologies. (F) Two D14 residues in loop regions of AcrIIA22 monomers (i) and (ii) are in close proximity. These residues are important for nicking activity and may bind divalent cations in cells under physiological pH. (G) A close view of a putative salt bridge between R30 of monomers (i)/(ii) and the peptide backbone of the C-terminus of monomers (iv)/(iii), respectively. AcrIIA22 monomers are colored as described in panel A. (PDF) [file pbio.3001428.s008.pdf]

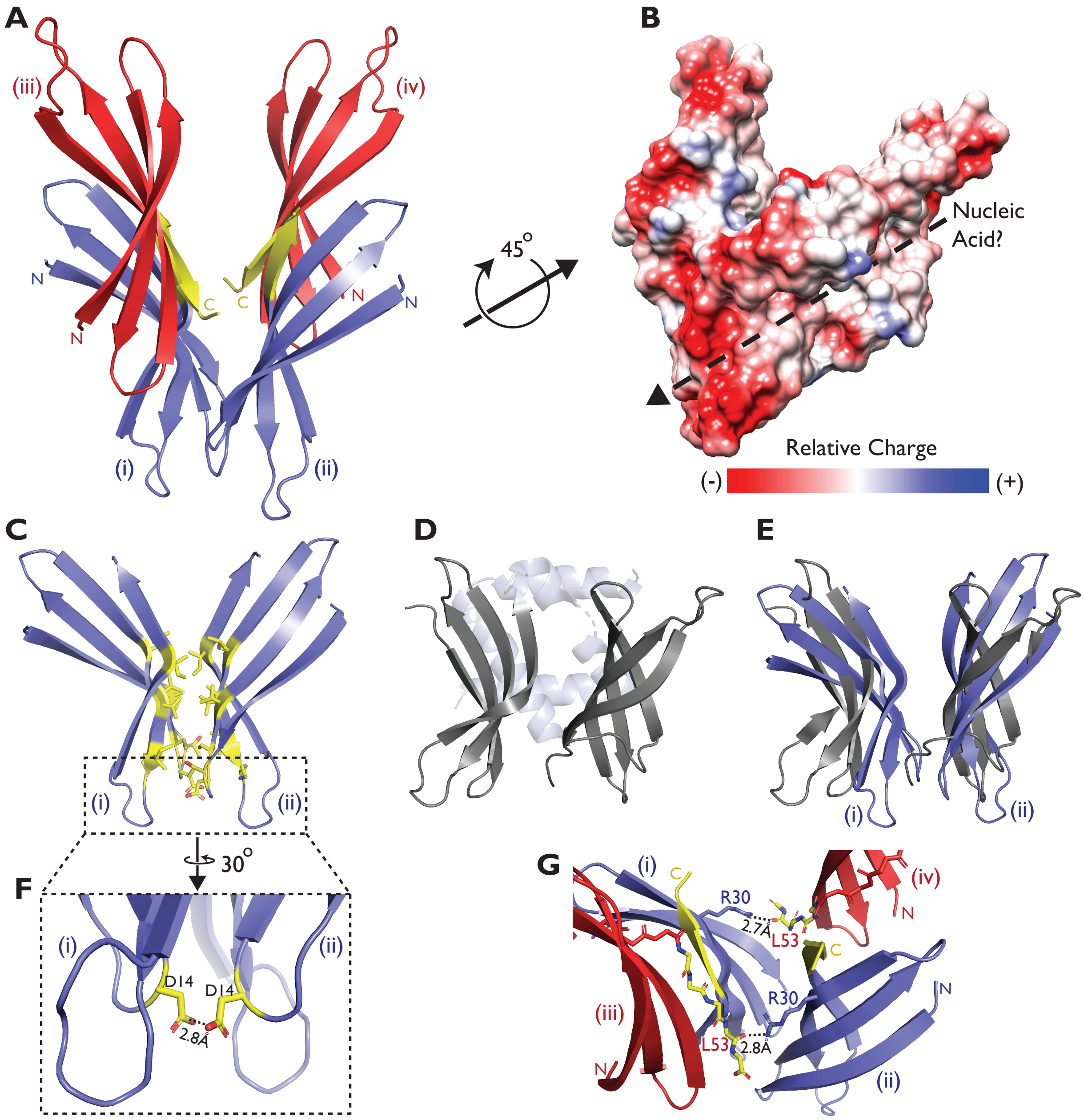

S8 Fig. **AcrIIA22 resembles a PC4-like protein.** (**A**) We present a ribbon diagram of a proposed AcrIIA22 tetramer, which requires binding between anti-parallel β-strands at the C-termini of AcrIIA22 monomers to form extended, concave β-sheets. This putative oligomerization interface is indicated by the regions highlighted in yellow. Each monomer in the proposed tetramer is labeled with lower-case Roman numerals (i-iv). (**B**) Space filling model of the tetrameric AcrIIA22 structure from panel A, with relative charge depicted, highlighting a groove (dashed line with arrowhead) that may accommodate nucleic acids (based on analogy to other PC4-like proteins). (**C**) AcrIIA22 monomers (i) and (ii) from the tetramer in panel A likely interact via a series of hydrophobic interactions, as indicated by the predominantly non-polar sidechains colored in yellow. The boxed region highlights residue D14, which is important for nicking activity and plasmid protection against SpyCas9, and is enlarged in panel F. (**D**) In conventional PC4-like family proteins, such as the putative single-stranded DNA binding protein from phage T5 depicted in gray (PDB:4BG7), the same topology of outward facing, concave β-sheets are instead stabilized via interactions between opposing α-helices (depicted in opaque light blue). (**E**) An overlay of β-sheets from AcrIIA22 (blue, PDB:7JTA) and the phage T5 PC4-like protein (gray, PDB:4BG7) illustrates their similar topologies. (**F**) Two D14 residues in loop regions of AcrIIA22 monomers (i) and (ii) are in close proximity. These residues are important for nicking activity and may bind divalent cations in cells under physiological pH. (**G**) A close view of a putative salt bridge between R30 of monomers (i) / (ii) and the peptide backbone of the C-terminus of monomers (iv) / (iii), respectively. AcrIIA22 monomers are colored as described in panel A.
