# Supplementary material for: The novel anti-CRISPR AcrIIA22 relieves DNA torsion in target plasmids and impairs SpyCas9 activity
Source: PLoS Biol. 2021 Oct 13;19(10):e3001428. doi: 10.1371/journal.pbio.3001428 (PMC8545432; doi:10.1371/journal.pbio.3001428)
Supplement: S9 Fig — (A) An in vivo plasmid protection assay. Asterisks depict statistically significant differences in plasmid retention under SpyCas9-inducing conditions with either wild-type AcrIIA22, a null mutant with an early stop codon, a 2-aa truncation, or a negative control gfp gene (adj. p < 0.005, Student t test, n = 3). The truncation mutant retains mild but severely impaired activity, as it protects a plasmid from SpyCas9 more effectively than a null mutant (p = 0.012) or GFP control (p = 0.015). All p-values were corrected for multiple hypotheses using Bonferroni method. (B) The 2-aa truncation mutant is impaired for nicking in vitro, relative to wild-type AcrIIA22. In both cases, 25 μM of protein was used following Ni-NTA–based purification of an N-terminal, His6-tagged construct. An asterisk (*) denotes significant differences between AcrIIA22-treated and untreated substrates (Student t test, p < 0.05, n = 3). Standard deviations are indicated by dashed lines (in most cases, the data points obscure these error bars). The individual numerical values that underlie the summary data in this figure may be found in S1 Data. CFU, colony-forming unit; SpyCas9, Streptococcus pyogenes Cas9. (PDF) [file pbio.3001428.s009.pdf]

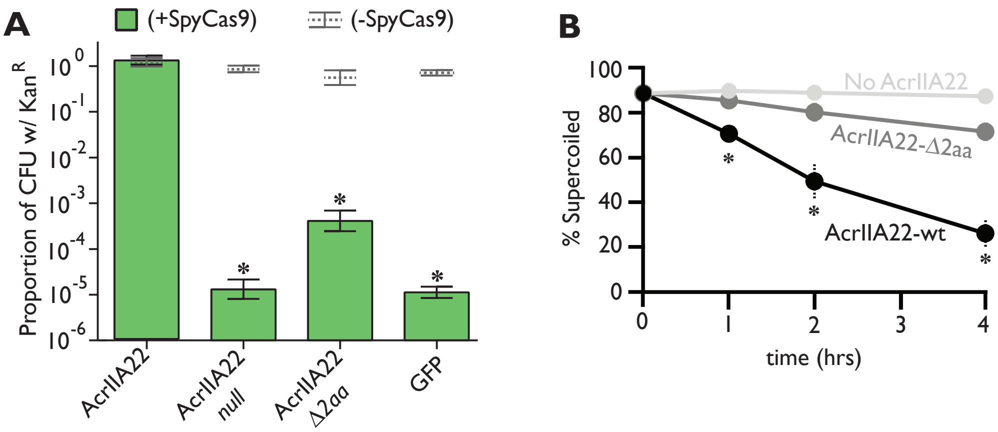

S9 Fig. A 2-aa truncation mutant of AcrIIA22 is impaired for SpyCas9 inhibition and nicking activity. (**A**) An *in vivo* plasmid protection assay. Asterisks depict statistically significant differences in plasmid retention under SpyCas9-inducing conditions with either wild-type AcrIIA22, a null mutant with an early stop codon, a 2-aa truncation, or a negative control *gfp* gene (adj. $p < 0.005$, Student's t-test, n=3). The truncation mutant retains mild but severely impaired activity, as it protects a plasmid from SpyCas9 more effectively than a null mutant ($p = 0.012$) or GFP control ($p = 0.015$). All p-values were corrected for multiple hypotheses using Bonferroni's method. (**B**) The 2-aa truncation mutant is impaired for nicking *in vitro*, relative to wild-type AcrIIA22. In both cases, 25μM of protein was used following NiNTA-based purification of an N-terminal, His6-tagged construct. An asterisk (*) denotes significant differences between AcrIIA22-treated and untreated substrates (Student's t-test, $p < 0.05$, n=3). Standard deviations are indicated by dashed lines (in most cases, the data points obscure these error bars). The individual numerical values that underlie the summary data in this figure may be found in S1_Data.
