# Supplementary material for: The novel anti-CRISPR AcrIIA22 relieves DNA torsion in target plasmids and impairs SpyCas9 activity
Source: PLoS Biol. 2021 Oct 13;19(10):e3001428. doi: 10.1371/journal.pbio.3001428 (PMC8545432; doi:10.1371/journal.pbio.3001428)
Supplement: S10 Fig — (A) A Coomassie stain of an N-terminally His6-tagged AcrIIA22 construct shows no copurifying proteins. (B) The nicking activity for this protein preparation (bottom) correlates with the intensity of the Coomassie-stained protein band across purification fractions (top). In each lane, SC plasmid DNA represents the unnicked fraction, whereas OC and linear DNA have been nicked at least once. (C) This panel is a quantification of the experiment depicted in panel B across all 13 fractions collected. (D) His6-AcrIIA22 nicks SC plasmids in a time- and concentration-dependent manner. A decrease in the proportion of SC plasmid DNA indicates nicking activity, as depicted in Fig 5B. (E) A silver stain of a C-terminally twin-strep-tagged AcrIIA22 construct shows no copurifying proteins. Equal volumes of each protein fraction were loaded in each lane for all samples. Fraction 4 was concentrated and used for all in vitro experiments. (F) A C-terminal, but not N-terminal twin-strep tag, is compatible with AcrIIA22’s ability to protect a target plasmid from SpyCas9 elimination in vivo. Statistically significant differences in plasmid retention between SpyCas9-inducing and noninducing conditions were determined via a Student t test (n = 3); “**” indicates p ≤ 0.001. All p-values were adjusted for multiple hypotheses using the Bonferroni correction. (G) The D14A mutation in AcrIIA22 impairs nicking activity. Over time, the wild-type AcrIIA22-twin-strep construct consistently converts a higher fraction of plasmid DNA from its SC form to an OC conformation than a D14A mutant. Control plasmids include a miniprepped sample and sample pretreated with the commercial nickase, Nb.BssSI. Reaction times are indicated to the right of each image. (H) AcrIIA22a (Fig 3B) is impaired for nicking activity relative to AcrIIA22. As in panel G, both constructs were purified via C-terminal twin-strep tags. The individual numerical values and original images for the data presented in this figure may be f [file pbio.3001428.s010.pdf]

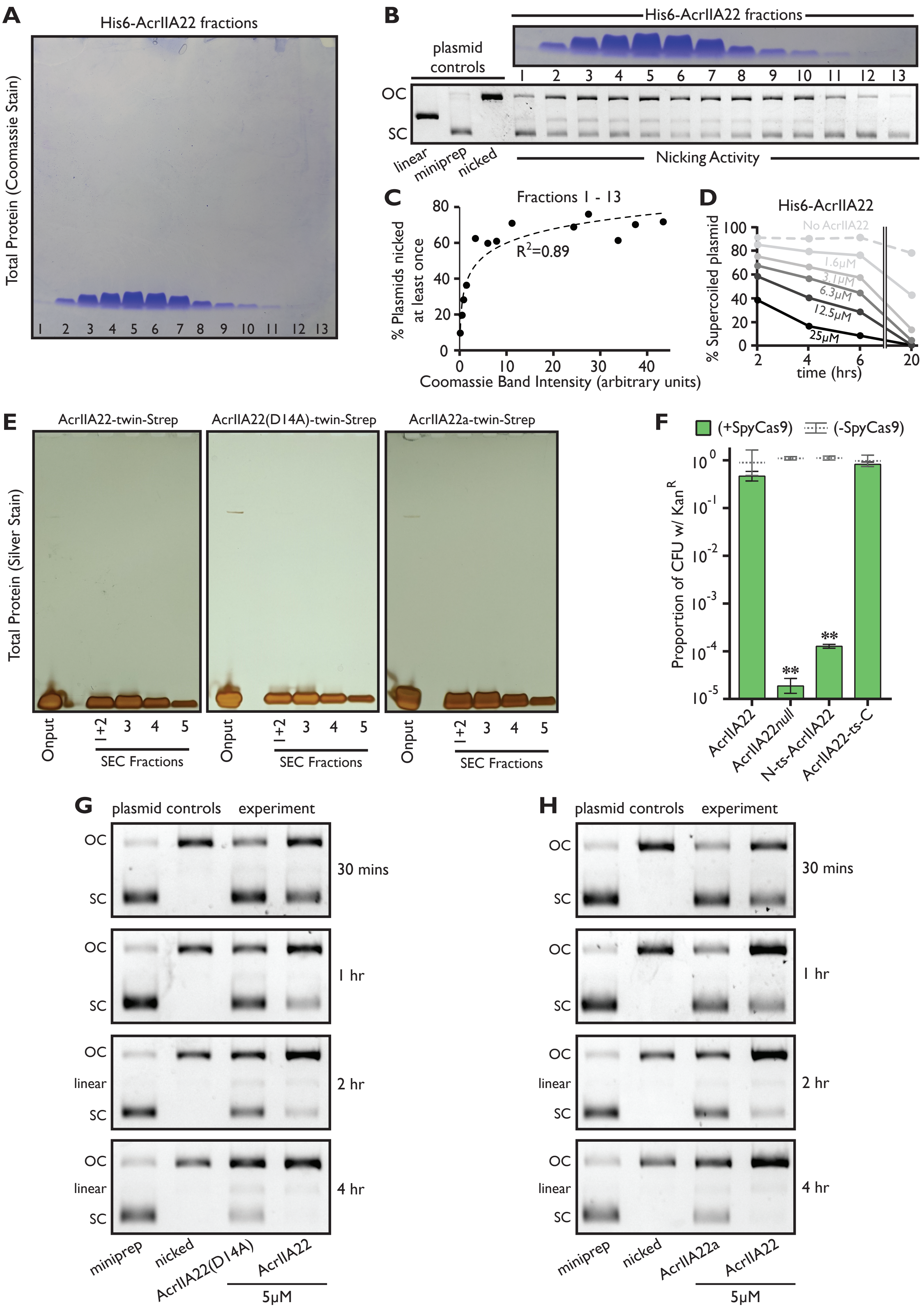

A
His6-AcrIIA22 fractions
Total Protein (Coomassie Stain)
1 2 3 4 5 6 7 8 9 10 11 12 13
B
plasmid controls
His6-AcrIIA22 fractions
1 2 3 4 5 6 7 8 9 10 11 12 13
OC
SC
linear
miniprep
nicked
Nicking Activity
C
Fractions 1 - 13
% Plasmids nicked at least once
R2=0.89
Coomassie Band Intensity (arbitrary units)
D
His6-AcrIIA22
No AcrIIA22
1.6μM
3.1μM
6.3μM
12.5μM
25μM
% Supercoiled plasmid
time (hrs)
E
AcrIIA22-twin-Strep
AcrIIA22(D14A)-twin-Strep
AcrIIA22a-twin-Strep
Total Protein (Silver Stain)
Onput
1+2 3 4 5
SEC Fractions
F
(+SpyCas9)
(-SpyCas9)
Proportion of CFU w/ KanR
**
AcrIIA22
AcrIIA22null
N-ts-AcrIIA22
AcrIIA22-ts-C
G
plasmid controls
experiment
OC
SC
linear
30 mins
1 hr
2 hr
4 hr
miniprep
nicked
AcrIIA22(D14A)
AcrIIA22
5μM
H
plasmid controls
experiment
OC
SC
linear
30 mins
1 hr
2 hr
4 hr
miniprep
nicked
AcrIIA22a
AcrIIA22
5μM

S10 Fig.

S10 Fig. **AcrIIA22 nicks supercoiled plasmids.** (**A**) A Coomassie stain of an N-terminally His6-tagged AcrIIA22 construct shows no co-purifying proteins. (**B**) The nicking activity for this protein preparation (bottom) correlates with the intensity of the Coomassie-stained protein band across purification fractions (top). In each lane, supercoiled (SC) plasmid DNA represents the un-nicked fraction whereas open circle (OC) and linear DNA have been nicked at least once. (**C**) This panel is a quantification of the experiment depicted in panel B across all 13 fractions collected. (**D**) His6-AcrIIA22 nicks supercoiled plasmids in a time and concentration dependent manner. A decrease in the proportion of supercoiled plasmid DNA indicates nicking activity, as depicted in Fig 5B. (**E**) A silver stain of a C-terminally twin-strep-tagged AcrIIA22 construct shows no co-purifying proteins. Equal volumes of each protein fraction were loaded in each lane, for all samples. Fraction 4 was concentrated and used for all *in vitro* experiments. (**F**) A C-terminal, but not N-terminal twin-strep tag is compatible with AcrIIA22's ability to protect a target plasmid from SpyCas9 elimination *in vivo*. Statistically significant differences in plasmid retention between SpyCas9-inducing and non-inducing conditions were determined via a Student's t-test (n=3); '**' indicates $p \leq 0.001$. All p-values were adjusted for multiple hypotheses using the Bonferroni correction. (**G**) The D14A mutation in AcrIIA22 impairs nicking activity. Over time, the wild-type AcrIIA22-twin-strep construct consistently converts a higher fraction of plasmid DNA from its supercoiled (SC) form to an open-circle (OC) conformation than a D14A mutant. Control plasmids include a miniprepped sample and sample pre-treated with the commercial nickase, Nb.BssSI. Reaction times are indicated to the right of each image. (**H**) AcrIIA22a (Fig 3B) is impaired for nicking activity relative to AcrIIA22. As in panel G, both constructs were purified via C-terminal twin-strep tags. The individual numerical values and original images for the data presented in this figure may be found in S1_Data and S1_raw_images, respectively.
