# Supplementary material for: The novel anti-CRISPR AcrIIA22 relieves DNA torsion in target plasmids and impairs SpyCas9 activity
Source: PLoS Biol. 2021 Oct 13;19(10):e3001428. doi: 10.1371/journal.pbio.3001428 (PMC8545432; doi:10.1371/journal.pbio.3001428)
Supplement: S11 Fig — (A) We present the impact of different divalent cations on AcrIIA22’s nicking activity, which is highest with Mg2+, Mn2+, and Co2+. (B) The OC plasmid product persists through phenol-chloroform extraction following AcrIIA22 treatment, indicating that it directly results from AcrIIA22’s nicking activity. OC, open-circle; SC, supercoiled. (PDF) [file pbio.3001428.s011.pdf]

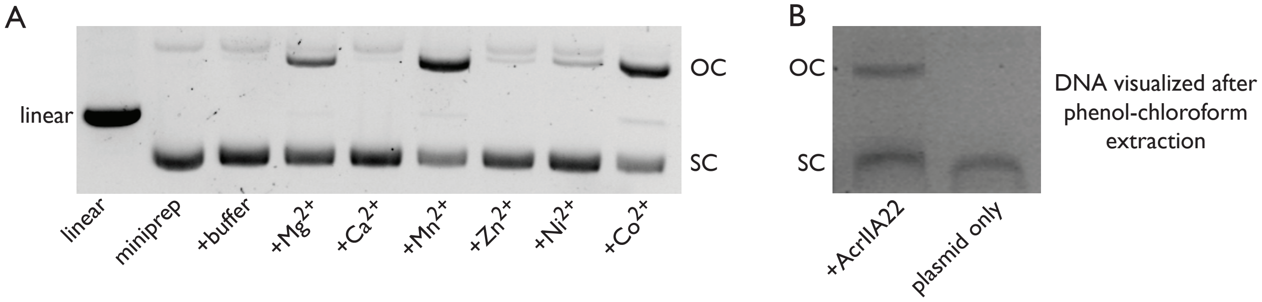

S11 Fig. Divalent cations influence AcrIIA22's nicking activity. (**A**) We present the impact of different divalent cations on AcrIIA22's nicking activity, which is highest with Mg^{2+}, Mn^{2+}, and Co^{2+}. OC, open-circle plasmid form. SC, supercoiled plasmid. (**B**) The open-circle plasmid product persists through phenol-chloroform extraction following AcrIIA22 treatment, indicating that it directly results from AcrIIA22's nicking activity.
