# Supplementary material for: The novel anti-CRISPR AcrIIA22 relieves DNA torsion in target plasmids and impairs SpyCas9 activity
Source: PLoS Biol. 2021 Oct 13;19(10):e3001428. doi: 10.1371/journal.pbio.3001428 (PMC8545432; doi:10.1371/journal.pbio.3001428)
Supplement: S1 Table — (PDF) [file pbio.3001428.s012.pdf]

S1 Table. Whether all known anti-CRISPRs can bind Cas proteins or inhibit their cleavage activity as purified proteins.

Acr	Binds cognate Cas protein?	Inhibit as pure proteins?	References
AcrIIA1	Yes	No	(Osuna et al., 2020)
AcrIIA2	Yes	Yes	(Jiang et al., 2019; Liu et al., 2019)
AcrIIA3	unknown	unknown	(Rauch et al., 2017)
AcrIIA4	Yes	Yes	(Dong et al., 2017; Shin et al., 2017; Yang and Patel, 2017)
AcrIIA5	Yes	Yes	(An et al., 2020; Garcia et al., 2019; Song et al., 2019)
AcrIIA6	Yes	Yes	(Fuchsbauer et al., 2019)
AcrIIA7	No	Yes	(Uribe et al., 2019)
AcrIIA8	Yes	Yes	(Uribe et al., 2019)
AcrIIA9	Yes	Yes	(Uribe et al., 2019)
AcrIIA10	Yes	Yes	(Uribe et al., 2019)
AcrIIA11	Yes	Yes	(Forsberg et al., 2019)
AcrIIA12	probable	Yes	(Eitzinger et al., 2020; Osuna et al., 2020)
AcrIIA13	unknown	Yes	(Watters et al., 2020)
AcrIIA14	unknown	Yes	(Watters et al., 2020)
AcrIIA15	unknown	Yes	(Watters et al., 2020)
AcrIIA16	Yes	Yes	(Mahendra et al., 2020)
AcrIIA17	Yes	No	(Mahendra et al., 2020)
AcrIIA18	Yes	No	(Mahendra et al., 2020)
AcrIIA19	Yes	No	(Mahendra et al., 2020)
AcrIIA20	unknown	Yes	(Eitzinger et al., 2020)
AcrIIA21	unknown	Yes	(Eitzinger et al., 2020)
**AcrIIA22**	**No**	**No**	**This study**
AcrIIA23	unknown	unknown	(Varble et al., 2020)
AcrIIC1	Yes	Yes	(Pawluk et al., 2016)
AcrIIC2	Yes	Yes	(Pawluk et al., 2016)
AcrIIC3	Yes	Yes	(Pawluk et al., 2016)
AcrIIC4	Yes	Yes	(Lee et al., 2018)
AcrIIC5	Yes	Yes	(Lee et al., 2018)
AcrVA1	Yes	Yes	(Knott et al., 2019b; Watters et al., 2018; Zhang et al., 2019)
AcrVA2	unknown	unknown	(Marino et al., 2018)
AcrVA3	unknown	unknown	(Marino et al., 2018)

AcrVA4	Yes	Yes	(Knott et al., 2019a; Knott et al., 2019b; Watters et al., 2018; Zhang et al., 2019)
AcrVA5	transiently	Yes	(Knott et al., 2019b; Watters et al., 2018; Zhang et al., 2019)
AcrVIA1(Lse)	Yes	Yes	(Meeske et al., 2020)
AcrVIA1(Lwa)	Yes	unknown	(Lin et al., 2020)
AcrVIA2	Yes	unknown	(Lin et al., 2020)
AcrVIA3	Yes	unknown	(Lin et al., 2020)
AcrVIA4	Yes	unknown	(Lin et al., 2020)
AcrVIA5	Yes	unknown	(Lin et al., 2020)
AcrVIA6	Yes	unknown	(Lin et al., 2020)
AcrVIA7	unknown	unknown	(Lin et al., 2020)
AcrIB1	unknown	unknown	(Lin et al., 2020)
AcrIC1	unknown	unknown	(Leon et al., 2020)
AcrIC2	probable	unknown	(Leon et al., 2020)
AcrIC3	unknown	unknown	(Leon et al., 2020)
AcrIC4	probable	unknown	(Leon et al., 2020)
AcrIC5	probable	unknown	(Leon et al., 2020)
AcrIC6	unknown	unknown	(Leon et al., 2020)
AcrIC7	probable	unknown	(Leon et al., 2020)
AcrIC8	probable	unknown	(Leon et al., 2020)
AcrID1	Yes	unknown	(He et al., 2018)
AcrIE1	Yes	unknown	(Pawluk et al., 2017)
AcrIE2	unknown	unknown	(Pawluk et al., 2014)
AcrIE3	probable	unknown	(Stanley, 2018)
AcrIE4	unknown	unknown	(Pawluk et al., 2014)
AcrIE5	unknown	unknown	(Pawluk et al., 2014)
AcrIE6	unknown	unknown	(Pawluk et al., 2014)
AcrIE7	unknown	unknown	(Pawluk et al., 2014)
AcrIE4-IF7	unknown	unknown	(Marino et al., 2018)
AcrIE8	unknown	unknown	(Pinilla-Redondo et al., 2020)
AcrIF1	Yes	unknown	(Bondy-Denomy et al., 2015; Chowdhury et al., 2017; Guo et al., 2017)
AcrIF2	Yes	unknown	(Bondy-Denomy et al., 2015; Chowdhury et al., 2017; Guo et al., 2017)
AcrIF3	Yes	unknown	(Bondy-Denomy et al., 2015; Wang et al., 2016a; Wang et al., 2016b)
AcrIF4	Yes	unknown	(Bondy-Denomy et al., 2015)
AcrIF5	unknown	unknown	(Bondy-Denomy et al., 2013)

AcrIF6	Yes	Yes	(Zhang et al., 2020)
AcrIF7	Yes	unknown	(Hirschi et al., 2020)
AcrIF8	Yes	Yes	(Zhang et al., 2020)
AcrIF9	Yes	Yes	(Hirschi et al., 2020; Zhang et al., 2020)
AcrIF10	Yes	unknown	(Guo et al., 2017)
AcrIF11	unknown	unknown	(Marino et al., 2018)
AcrIF12	unknown	unknown	(Marino et al., 2018)
AcrIF13	unknown	unknown	(Marino et al., 2018)
AcrIF14	unknown	unknown	(Marino et al., 2018)
AcrIF15	probable	unknown	(Pinilla-Redondo et al., 2020)
AcrIF16	unknown	unknown	(Pinilla-Redondo et al., 2020)
AcrIF17	unknown	unknown	(Pinilla-Redondo et al., 2020)
AcrIF18	probable	unknown	(Pinilla-Redondo et al., 2020)
AcrIF19	unknown	unknown	(Pinilla-Redondo et al., 2020)
AcrIF20	unknown	unknown	(Pinilla-Redondo et al., 2020)
AcrIF21	unknown	unknown	(Pinilla-Redondo et al., 2020)
AcrIF22	unknown	unknown	(Pinilla-Redondo et al., 2020)
AcrIF23	unknown	unknown	(Pinilla-Redondo et al., 2020)
AcrIF24	unknown	unknown	(Pinilla-Redondo et al., 2020)
AcrIII-1	No (degrades CA4 second messenger)	No	(Athukoralage et al., 2020)
AcrIIIB1	Yes	unknown	(Bhoobalan-Chitty et al., 2019)

References S1 Table.

An, S.Y., Ka, D., Kim, I., Kim, E.-H., Kim, N.-K., Bae, E., and Suh, J.-Y. (2020). Intrinsic disorder is essential for Cas9 inhibition of anti-CRISPR AcrIIA5. Nucleic Acids Research *48*, 7584-7594.

Athukoralage, J.S., McMahon, S.A., Zhang, C., Gruschow, S., Graham, S., Krupovic, M., Whitaker, R.J., Gloster, T.M., and White, M.F. (2020). An anti-CRISPR viral ring nuclease subverts type III CRISPR immunity. Nature *577*, 572-575.

Bhoobalan-Chitty, Y., Johansen, T.B., Di Cianni, N., and Peng, X. (2019). Inhibition of Type III CRISPR-Cas Immunity by an Archaeal Virus-Encoded Anti-CRISPR Protein. Cell *179*, 448-458 e411.

Bondy-Denomy, J., Garcia, B., Strum, S., Du, M., Rollins, M.F., Hidalgo-Reyes, Y., Wiedenheft, B., Maxwell, K.L., and Davidson, A.R. (2015). Multiple mechanisms for CRISPR-Cas inhibition by anti-CRISPR proteins. Nature *526*, 136-139.

Bondy-Denomy, J., Pawluk, A., Maxwell, K.L., and Davidson, A.R. (2013). Bacteriophage genes that inactivate the CRISPR/Cas bacterial immune system. Nature *493*, 429-432.

Chowdhury, S., Carter, J., Rollins, M.F., Golden, S.M., Jackson, R.N., Hoffmann, C., Nosaka, L., Bondy-Denomy, J., Maxwell, K.L., Davidson, A.R., *et al.* (2017). Structure Reveals Mechanisms of Viral Suppressors that Intercept a CRISPR RNA-Guided Surveillance Complex. Cell *169*, 47-57 e11.

Dong, Guo, M., Wang, S., Zhu, Y., Wang, S., Xiong, Z., Yang, J., Xu, Z., and Huang, Z. (2017). Structural basis of CRISPR-SpyCas9 inhibition by an anti-CRISPR protein. Nature *546*, 436-439.

Eitzinger, S., Asif, A., Watters, K.E., Iavarone, A.T., Knott, G.J., Doudna, J.A., and Minhas, F. (2020). Machine learning predicts new anti-CRISPR proteins. Nucleic Acids Res *48*, 4698-4708.

Forsberg, K.J., Bhatt, I.V., Schmidtke, D.T., Javanmardi, K., Dillard, K.E., Stoddard, B.L., Finkelstein, I.J., Kaiser, B.K., and Malik, H.S. (2019). Functional metagenomics-guided discovery of potent Cas9 inhibitors in the human microbiome. Elife *8*.

Fuchsbauer, O., Swuec, P., Zimberger, C., Amigues, B., Levesque, S., Agudelo, D., Duringer, A., Chaves-Sanjuan, A., Spinelli, S., Rousseau, G.M., *et al.* (2019). Cas9 Allosteric Inhibition by the Anti-CRISPR Protein AcrIIA6. Mol Cell *76*, 922-937 e927.

Garcia, B., Lee, J., Edraki, A., Hidalgo-Reyes, Y., Erwood, S., Mir, A., Trost, C.N., Seroussi, U., Stanley, S.Y., Cohn, R.D., *et al.* (2019). Anti-CRISPR AcrIIA5 Potently Inhibits All Cas9 Homologs Used for Genome Editing. Cell Rep *29*, 1739-1746 e1735.

Guo, T.W., Bartesaghi, A., Yang, H., Falconieri, V., Rao, P., Merk, A., Eng, E.T., Raczkowski, A.M., Fox, T., Earl, L.A., *et al.* (2017). Cryo-EM Structures Reveal Mechanism and Inhibition of DNA Targeting by a CRISPR-Cas Surveillance Complex. Cell *171*, 414-426 e412.

He, F., Bhoobalan-Chitty, Y., Van, L.B., Kjeldsen, A.L., Dedola, M., Makarova, K.S., Koonin, E.V., Brodersen, D.E., and Peng, X. (2018). Anti-CRISPR proteins encoded by archaeal lytic viruses inhibit subtype I-D immunity. Nat Microbiol *3*, 461-469.

Hirschi, M., Lu, W.T., Santiago-Frangos, A., Wilkinson, R., Golden, S.M., Davidson, A.R., Lander, G.C., and Wiedenheft, B. (2020). AcrIF9 tethers non-sequence specific dsDNA to the CRISPR RNA-guided surveillance complex. Nat Commun *11*, 2730.

Jiang, F., Liu, J.J., Osuna, B.A., Xu, M., Berry, J.D., Rauch, B.J., Nogales, E., Bondy-Denomy, J., and Doudna, J.A. (2019). Temperature-Responsive Competitive Inhibition of CRISPR-Cas9. Mol Cell *73*, 601-610 e605.

Knott, G.J., Cress, B.F., Liu, J.J., Thornton, B.W., Lew, R.J., Al-Shayeb, B., Rosenberg, D.J., Hammel, M., Adler, B.A., Lobba, M.J.*, et al.* (2019a). Structural basis for AcrVA4 inhibition of specific CRISPR-Cas12a. Elife *8*.

Knott, G.J., Thornton, B.W., Lobba, M.J., Liu, J.J., Al-Shayeb, B., Watters, K.E., and Doudna, J.A. (2019b). Broad-spectrum enzymatic inhibition of CRISPR-Cas12a. Nat Struct Mol Biol *26*, 315-321.

Lee, J., Mir, A., Edraki, A., Garcia, B., Amrani, N., Lou, H.E., Gainetdinov, I., Pawluk, A., Ibraheim, R., Gao, X.D.*, et al.* (2018). Potent Cas9 Inhibition in Bacterial and Human Cells by AcrIIC4 and AcrIIC5 Anti-CRISPR Proteins. mBio *9*.

Leon, L.M., Park, A.E., Borges, A.L., Zhang, J.Y., and Bondy-Denomy, J. (2020). Mobile element warfare via CRISPR and anti-CRISPR in Pseudomonas aeruginosa. bioRxiv, 2020.2006.2015.151498.

Lin, P., Qin, S., Pu, Q., Wang, Z., Wu, Q., Gao, P., Schettler, J., Guo, K., Li, R., Li, G.*, et al.* (2020). CRISPR-Cas13 Inhibitors Block RNA Editing in Bacteria and Mammalian Cells. Mol Cell *78*, 850-861 e855.

Liu, L., Yin, M., Wang, M., and Wang, Y. (2019). Phage AcrIIA2 DNA Mimicry: Structural Basis of the CRISPR and Anti-CRISPR Arms Race. Mol Cell *73*, 611-620 e613.

Mahendra, C., Christie, K.A., Osuna, B.A., Pinilla-Redondo, R., Kleinstiver, B.P., and Bondy-Denomy, J. (2020). Broad-spectrum anti-CRISPR proteins facilitate horizontal gene transfer. Nat Microbiol *5*, 620-629.

Marino, N.D., Zhang, J.Y., Borges, A.L., Sousa, A.A., Leon, L.M., Rauch, B.J., Walton, R.T., Berry, J.D., Joung, J.K., Kleinstiver, B.P.*, et al.* (2018). Discovery of widespread Type I and Type V CRISPR-Cas inhibitors. Science.

Meeske, A.J., Jia, N., Cassel, A.K., Kozlova, A., Liao, J., Wiedmann, M., Patel, D.J., and Marraffini, L.A. (2020). A phage-encoded anti-CRISPR enables complete evasion of type VI-A CRISPR-Cas immunity. Science *369*, 54-59.

Osuna, B.A., Karambelkar, S., Mahendra, C., Christie, K.A., Garcia, B., Davidson, A.R., Kleinstiver, B.P., Kilcher, S., and Bondy-Denomy, J. (2020). Listeria Phages Induce Cas9 Degradation to Protect Lysogenic Genomes. Cell Host Microbe *28*, 31-40 e39.

Pawluk, A., Amrani, N., Zhang, Y., Garcia, B., Hidalgo-Reyes, Y., Lee, J., Edraki, A., Shah, M., Sontheimer, E.J., Maxwell, K.L.*, et al.* (2016). Naturally Occurring Off-Switches for CRISPR-Cas9. Cell *167*, 1829-1838 e1829.

Pawluk, A., Bondy-Denomy, J., Cheung, V.H., Maxwell, K.L., and Davidson, A.R. (2014). A new group of phage anti-CRISPR genes inhibits the type I-E CRISPR-Cas system of Pseudomonas aeruginosa. MBio *5*, e00896.

Pawluk, A., Shah, M., Mejdani, M., Calmettes, C., Moraes, T.F., Davidson, A.R., and Maxwell, K.L. (2017). Disabling a Type I-E CRISPR-Cas Nuclease with a Bacteriophage-Encoded Anti-CRISPR Protein. MBio *8*.

Pinilla-Redondo, R., Shehreen, S., Marino, N.D., Fagerlund, R.D., Brown, C.M., Sørensen, S.J., Fineran, P.C., and Bondy-Denomy, J. (2020). Discovery of multiple anti-CRISPRs uncovers anti-defense gene clustering in mobile genetic elements. bioRxiv, 2020.2005.2022.110304.

Rauch, B.J., Silvis, M.R., Hultquist, J.F., Waters, C.S., McGregor, M.J., Krogan, N.J., and Bondy-Denomy, J. (2017). Inhibition of CRISPR-Cas9 with Bacteriophage Proteins. Cell *168*, 150-158 e110.

Shin, J., Jiang, F., Liu, J.-J., Bray, N.L., Rauch, B.J., Baik, S.H., Nogales, E., Bondy-Denomy, J., Corn, J.E., and Doudna, J.A. (2017). Disabling Cas9 by an anti-CRISPR DNA mimic. Science Advances *3*.

Song, G., Zhang, F., Zhang, X., Gao, X., Zhu, X., Fan, D., and Tian, Y. (2019). AcrIIA5 Inhibits a Broad Range of Cas9 Orthologs by Preventing DNA Target Cleavage. Cell Rep *29*, 2579-2589 e2574.

Stanley, S.Y. (2018). An Investigation of Bacteriophage Anti-CRISPR and Anti-CRISPR Associated Proteins. In Department of Molecular Genetics (http://hdl.handle.net/1807/97883: University of Toronto), pp. 120.

Uribe, R.V., van der Helm, E., Misiakou, M.A., Lee, S.W., Kol, S., and Sommer, M.O.A. (2019). Discovery and Characterization of Cas9 Inhibitors Disseminated across Seven Bacterial Phyla. Cell Host Microbe *25*, 233-241 e235.

Varble, A., Campisi, E., Euler, C.W., Fyodorova, J., Rostøl, J.T., Fischetti, V.A., and Marraffini, L.A. (2020). Integration of prophages into CRISPR loci remodels viral immunity in <em>Streptococcus pyogenes</em&gt. bioRxiv, 2020.2010.2009.333658.

Wang, J., Ma, J., Cheng, Z., Meng, X., You, L., Wang, M., Zhang, X., and Wang, Y. (2016a). A CRISPR evolutionary arms race: structural insights into viral anti-CRISPR/Cas responses. Cell Res *26*, 1165-1168.

Wang, X., Yao, D., Xu, J.G., Li, A.R., Xu, J., Fu, P., Zhou, Y., and Zhu, Y. (2016b). Structural basis of Cas3 inhibition by the bacteriophage protein AcrF3. Nat Struct Mol Biol *23*, 868-870.

Watters, K.E., Fellmann, C., Bai, H.B., Ren, S.M., and Doudna, J.A. (2018). Systematic discovery of natural CRISPR-Cas12a inhibitors. Science.

Watters, K.E., Shivram, H., Fellmann, C., Lew, R.J., McMahon, B., and Doudna, J.A. (2020). Potent CRISPR-Cas9 inhibitors from Staphylococcus genomes. Proc Natl Acad Sci U S A *117*, 6531-6539.

Yang, H., and Patel, D.J. (2017). Inhibition Mechanism of an Anti-CRISPR Suppressor AcrIIA4 Targeting SpyCas9. Mol Cell *67*, 117-127 e115.

Zhang, H., Li, Z., Daczkowski, C.M., Gabel, C., Mesecar, A.D., and Chang, L. (2019). Structural Basis for the Inhibition of CRISPR-Cas12a by Anti-CRISPR Proteins. Cell Host Microbe *25*, 815-826 e814.

Zhang, K., Wang, S., Li, S., Zhu, Y., Pintilie, G.D., Mou, T.C., Schmid, M.F., Huang, Z., and Chiu, W. (2020). Inhibition mechanisms of AcrF9, AcrF8, and AcrF6 against type I-F CRISPR-Cas complex revealed by cryo-EM. Proc Natl Acad Sci U S A *117*, 7176-7182.
