# Supplementary material for: The novel anti-CRISPR AcrIIA22 relieves DNA torsion in target plasmids and impairs SpyCas9 activity
Source: PLoS Biol. 2021 Oct 13;19(10):e3001428. doi: 10.1371/journal.pbio.3001428 (PMC8545432; doi:10.1371/journal.pbio.3001428)
Supplement: S2 Table — (PDF) [file pbio.3001428.s013.pdf]

S2 Table. PC4-like proteins with structural homology to AcrIIA22

Structural Homolog		Function	Similarity to AcrIIA22			
PDBID	Name	DNA/RNA Binding*	Zscore	r.m.s.d.	n-align	% A.A. ID
4bg7	PC4 putative transcriptional coactivator p15	DNA	6.2	2.5	54	15
3k44	*D. melanogaster* Pur-α	DNA/RNA	5.9	2.6	47	9
5fgp	Pur-α repeat I and II from *D. melanogaster*	DNA/RNA	5.6	2.1	48	8
3n8b	Pur-α from *B. burgdorferi*	DNA/RNA	5	2.8	48	6
2gje	Mitochondrial RNA Binding Protein (*T. brucei*)	RNA	4.9	2.5	52	8
5zkl	Protein of unknown function SP_0782, *S. pneumoniae*	DNA	4.7	3.6	52	12
5fgo	*D. melanogaster* Pur-α repeat III	No info	4.5	2.7	44	14
1pcf	Replication & transcription cofactor PC4 CTD	DNA	4.5	2.5	45	7
2ltt	Putative Uncharacterized Protein YDBC	DNA	4.5	2.8	50	12
4bhm	MoSub1-DNA PC4 transcription cofactor	DNA	3.9	2.8	45	4
3cm1	SSGA-like sporulation specific cell division protein	No info	2.8	3.7	47	13
1l3a	Transcription factor PBF-2 (P24, WHY1)	DNA	2.8	5	48	8
4ntq	Anti-toxin CdiI, *E. cloacae*	No info	2.7	3	49	12
3n1k	WHY2 transcription factor, *S. tuberosum*	DNA	2.6	2.8	52	4

*RNA/DNA binding data from (Janowski and Niessing, 2020).

Reference for S2 Table.

Janowski, R., and Niessing, D. (2020). The large family of PC4-like domains - similar folds and functions throughout all kingdoms of life. RNA Biol *17*, 1228-1238.
