# Supplementary material for: The novel anti-CRISPR AcrIIA22 relieves DNA torsion in target plasmids and impairs SpyCas9 activity
Source: PLoS Biol. 2021 Oct 13;19(10):e3001428. doi: 10.1371/journal.pbio.3001428 (PMC8545432; doi:10.1371/journal.pbio.3001428)
Supplement: S3 Table — All sequences and annotations are also available as supplemental data. (PDF) [file pbio.3001428.s014.pdf]

S3 Table. All sequences used in this study. Sequence names and databases are indicated. All sequences and annotations are also available as supplemental data. Sequences retrieved from Pasolli *et al.* refer to the following study: (Pasolli et al., 2019).

Contig No.	Sequence Name	How Used?	NCBI Nuc. ID	Pasolli et al SGB?	Pasolli et al Raw Assembly?	Pasolli Reconstructed Genome Name	SGB id	GTDB Taxonomy	External Data Available at:
1	4303_LiJ_2014__V1.UC63-0__bin.67_NODE_112_length_95405_cov_4.60675	Fig 2, find gene functions from 54 unique genomic loci; Fig 3 Acr Seq	n/a	Yes	No	LiJ_2014__V1.UC63-0__bin.67	4303	d__Bacteria; p__Firmicutes_A; c__Clostridia; o__Oscillospirales; f__Acutalibacteraceae; g__CAG-217	http://segatalab.cibio.unitn.it/data/Pasolli_et_al.html
2	Bengtsson-PalmeJ_2015__TRAVELRES9_NODE_4_length_538580_cov_9.43148	find gene functions from 54 unique genomic loci	n/a	No	Yes	n/a	n/a	d__Bacteria; p__Firmicutes_A; c__Clostridia; o__Oscillospirales; f__Acutalibacteraceae; g__CAG-217 (inferred)	http://segatalab.cibio.unitn.it/data/Pasolli_et_al.html
3	ChengpingW_2017__AS9raw_NODE_922_length_27664_cov_3.49089	find gene functions from 54 unique genomic loci	n/a	No	Yes	n/a	n/a	d__Bacteria; p__Firmicutes_A; c__Clostridia; o__Oscillospirales; f__Acutalibacteraceae; g__CAG-217 (inferred)	http://segatalab.cibio.unitn.it/data/Pasolli_et_al.html
4	CosteaPI_2017__SID713B025-11-0-0_NODE_4_length_351620_cov_7.46108	find gene functions from 54 unique genomic loci	n/a	No	Yes	n/a	n/a	d__Bacteria; p__Firmicutes_A; c__Clostridia; o__Oscillospirales; f__Acutalibacteraceae; g__CAG-217 (inferred)	http://segatalab.cibio.unitn.it/data/Pasolli_et_al.html
5	BritoIL_2016__M1.64.ST_NODE_47_length_140472_cov_9.49805	find gene functions from 54 unique genomic loci; Fig 3 Acr Seq	n/a	No	Yes	n/a	n/a	d__Bacteria; p__Firmicutes_A; c__Clostridia; o__Oscillospirales; f__Acutalibacteraceae; g__CAG-217 (inferred)	http://segatalab.cibio.unitn.it/data/Pasolli_et_al.html
6	BritoIL_2016__M2.57.ST_NODE_3_length_405636_cov_14.0428	find gene functions from 54 unique genomic loci	n/a	No	Yes	n/a	n/a	d__Bacteria; p__Firmicutes_A; c__Clostridia; o__Oscillospirales; f__Acutalibacteraceae; g__CAG-217 (inferred)	http://segatalab.cibio.unitn.it/data/Pasolli_et_al.html
7	BritoIL_2016__WL.14.ST_NODE_13_length_259523_cov_10.8408	find gene functions from 54 unique genomic loci	n/a	No	Yes	n/a	n/a	d__Bacteria; p__Firmicutes_A; c__Clostridia; o__Oscillospirales; f__Acutalibacteraceae; g__CAG-217 (inferred)	http://segatalab.cibio.unitn.it/data/Pasolli_et_al.html
8	ChengpingW_2017__AS67raw_NODE_2_length_439177_cov_9.00174	Fig 2, find gene functions from 54 unique genomic loci; Fig 3 Acr Seq	n/a	No	Yes	n/a	n/a	d__Bacteria; p__Firmicutes_A; c__Clostridia; o__Oscillospirales; f__Acutalibacteraceae; g__CAG-217 (inferred)	http://segatalab.cibio.unitn.it/data/Pasolli_et_al.html
9	CM_madagascar__A90_04_1FE_NODE_125_length_81453_cov_9.00904	find gene functions from 54 unique genomic loci	n/a	No	Yes	n/a	n/a	d__Bacteria; p__Firmicutes_A; c__Clostridia; o__Oscillospirales; f__Acutalibacteraceae; g__CAG-217 (inferred)	http://segatalab.cibio.unitn.it/data/Pasolli_et_al.html
10	CM_madagascar__V12_01_2FE_NODE_5_length_202628_cov_9.50435	find gene functions from 54 unique genomic loci	n/a	No	Yes	n/a	n/a	d__Bacteria; p__Firmicutes_A; c__Clostridia; o__Oscillospirales; f__Acutalibacteraceae; g__CAG-217 (inferred)	http://segatalab.cibio.unitn.it/data/Pasolli_et_al.html
11	CosteaPI_2017__SID713A046-11-0-0_NODE_322_length_69000_cov_4.32987	find gene functions from 54 unique genomic loci; Fig 3 Acr Seq	n/a	No	Yes	n/a	n/a	d__Bacteria; p__Firmicutes_A; c__Clostridia; o__Oscillospirales; f__Acutalibacteraceae; g__CAG-217 (inferred)	http://segatalab.cibio.unitn.it/data/Pasolli_et_al.html
12	CosteaPI_2017__SID713A045-11-0-0_NODE_78_length_64886_cov_3.97493	find gene functions from 54 unique genomic loci	n/a	No	Yes	n/a	n/a	d__Bacteria; p__Firmicutes_A; c__Clostridia; o__Oscillospirales; f__Acutalibacteraceae; g__CAG-217 (inferred)	http://segatalab.cibio.unitn.it/data/Pasolli_et_al.html
13	CosteaPI_2017__SID713A004-11-0-0_NODE_1_length_647860_cov_14.1013	find gene functions from 54 unique genomic loci; Fig 3 Acr Seq	n/a	No	Yes	n/a	n/a	d__Bacteria; p__Firmicutes_A; c__Clostridia; o__Oscillospirales; f__Acutalibacteraceae; g__CAG-217 (inferred)	http://segatalab.cibio.unitn.it/data/Pasolli_et_al.html
14	CosteaPI_2017__peacemaker-11-60-	find gene functions from 54 unique genomic loci	n/a	No	Yes	n/a	n/a	d__Bacteria; p__Firmicutes_A; c__Clostridia; o__Oscillospirales; f__Acutalibacteraceae; g__CAG-217 (inferred)	http://segatalab.cibio.unitn.it/data/Pasolli_et_al.html

	0_NODE_48_length_49378_cov_15.5445								
**15**	CosteaPI_2017__SID713A063-11-90-0_NODE_2082_length_16960_cov_2.98527	find gene functions from 54 unique genomic loci	n/a	No	Yes	n/a	n/a	d__Bacteria; p__Firmicutes_A; c__Clostridia; o__Oscillospirales; f__Acutalibacteraceae; g__CAG-217 (inferred)	http://segatalab.cibio.unitn.it/data/Pasolli_et_al.html
**16**	CosteaPI_2017__SID713A088-11-0-0_NODE_89_length_113297_cov_5.77445	find gene functions from 54 unique genomic loci; Fig 3 Acr Seq	n/a	No	Yes	n/a	n/a	d__Bacteria; p__Firmicutes_A; c__Clostridia; o__Oscillospirales; f__Acutalibacteraceae; g__CAG-217 (inferred)	http://segatalab.cibio.unitn.it/data/Pasolli_et_al.html
**17**	CosteaPI_2017__SID713A062-11-0-0_NODE_38_length_192196_cov_4.03099	Find gene functions from 54 unique genomic loci	n/a	No	Yes	n/a	n/a	d__Bacteria; p__Firmicutes_A; c__Clostridia; o__Oscillospirales; f__Acutalibacteraceae; g__CAG-217 (inferred)	http://segatalab.cibio.unitn.it/data/Pasolli_et_al.html
**18**	CosteaPI_2017__SID713B051-11-0-0_NODE_14_length_298619_cov_7.20988	find gene functions from 54 unique genomic loci	n/a	No	Yes	n/a	n/a	d__Bacteria; p__Firmicutes_A; c__Clostridia; o__Oscillospirales; f__Acutalibacteraceae; g__CAG-217 (inferred)	http://segatalab.cibio.unitn.it/data/Pasolli_et_al.html
**19**	FengQ_2015__SID31872_NODE_2_length_392843_cov_5.93617	find gene functions from 54 unique genomic loci	n/a	No	Yes	n/a	n/a	d__Bacteria; p__Firmicutes_A; c__Clostridia; o__Oscillospirales; f__Acutalibacteraceae; g__CAG-217 (inferred)	http://segatalab.cibio.unitn.it/data/Pasolli_et_al.html
**20**	FengQ_2015__SID530258_NODE_5_length_350476_cov_17.595	find gene functions from 54 unique genomic loci; Fig 3 Acr Seq	n/a	No	Yes	n/a	n/a	d__Bacteria; p__Firmicutes_A; c__Clostridia; o__Oscillospirales; f__Acutalibacteraceae; g__CAG-217 (inferred)	http://segatalab.cibio.unitn.it/data/Pasolli_et_al.html
**21**	FengQ_2015__SID530373_NODE_21_length_272157_cov_9.73468	find gene functions from 54 unique genomic loci	n/a	No	Yes	n/a	n/a	d__Bacteria; p__Firmicutes_A; c__Clostridia; o__Oscillospirales; f__Acutalibacteraceae; g__CAG-217 (inferred)	http://segatalab.cibio.unitn.it/data/Pasolli_et_al.html
**22**	HeQ_2017__SZAXPI029561-52_NODE_1_length_502752_cov_8.09488	find gene functions from 54 unique genomic loci; Fig 3 Acr Seq	n/a	No	Yes	n/a	n/a	d__Bacteria; p__Firmicutes_A; c__Clostridia; o__Oscillospirales; f__Acutalibacteraceae; g__CAG-217 (inferred)	http://segatalab.cibio.unitn.it/data/Pasolli_et_al.html
**23**	HeQ_2017__SZAXPI029575-90_NODE_229_length_94918_cov_3.79903	find gene functions from 54 unique genomic loci	n/a	No	Yes	n/a	n/a	d__Bacteria; p__Firmicutes_A; c__Clostridia; o__Oscillospirales; f__Acutalibacteraceae; g__CAG-217 (inferred)	http://segatalab.cibio.unitn.it/data/Pasolli_et_al.html
**24**	KarlssonFH_2013__S463_NODE_1_length_570037_cov_16.3973	Fig 2, find gene functions from 54 unique genomic loci	n/a	No	Yes	n/a	n/a	d__Bacteria; p__Firmicutes_A; c__Clostridia; o__Oscillospirales; f__Acutalibacteraceae; g__CAG-217 (inferred)	http://segatalab.cibio.unitn.it/data/Pasolli_et_al.html
**25**	LiJ_2014__O2.UC12-1_NODE_323_length_49995_cov_5.04395	find gene functions from 54 unique genomic loci	n/a	No	Yes	n/a	n/a	d__Bacteria; p__Firmicutes_A; c__Clostridia; o__Oscillospirales; f__Acutalibacteraceae; g__CAG-217 (inferred)	http://segatalab.cibio.unitn.it/data/Pasolli_et_al.html
**26**	LiJ_2014__V1.FI02_NODE_274_length_84286_cov_3.49253	find gene functions from 54 unique genomic loci	n/a	No	Yes	n/a	n/a	d__Bacteria; p__Firmicutes_A; c__Clostridia; o__Oscillospirales; f__Acutalibacteraceae; g__CAG-217 (inferred)	http://segatalab.cibio.unitn.it/data/Pasolli_et_al.html
**27**	LiJ_2017__H1M413815_NODE_71_length_81514_cov_18.301	find gene functions from 54 unique genomic loci	n/a	No	Yes	n/a	n/a	d__Bacteria; p__Firmicutes_A; c__Clostridia; o__Oscillospirales; f__Acutalibacteraceae; g__CAG-217 (inferred)	http://segatalab.cibio.unitn.it/data/Pasolli_et_al.html
**28**	LiJ_2017__H2M514909_NODE_68_length_69076_cov_10.283	find gene functions from 54 unique genomic loci	n/a	No	Yes	n/a	n/a	d__Bacteria; p__Firmicutes_A; c__Clostridia; o__Oscillospirales; f__Acutalibacteraceae; g__CAG-217 (inferred)	http://segatalab.cibio.unitn.it/data/Pasolli_et_al.html
**29**	LiuW_2016__SRR3992969_NODE_1149_length_18999_cov_8.45033	find gene functions from 54 unique genomic loci; Fig 3 Acr Seq	n/a	No	Yes	n/a	n/a	d__Bacteria; p__Firmicutes_A; c__Clostridia; o__Oscillospirales; f__Acutalibacteraceae; g__CAG-217 (inferred)	http://segatalab.cibio.unitn.it/data/Pasolli_et_al.html

**30**	LiuW_2016__SRR3992984_NODE_127_length_61384_cov_18.0593	find gene functions from 54 unique genomic loci	n/a	No	Yes	n/a	n/a	d__Bacteria; p__Firmicutes_A; c__Clostridia; o__Oscillospirales; f__Acutalibacteraceae; g__CAG-217 (inferred)	http://segatalab.cibio.unitn.it/data/Pasolli_et_al.html
**31**	LiuW_2016__SRR3993014_NODE_8_length_143441_cov_89.3981	find gene functions from 54 unique genomic loci	n/a	No	Yes	n/a	n/a	d__Bacteria; p__Firmicutes_A; c__Clostridia; o__Oscillospirales; f__Acutalibacteraceae; g__CAG-217 (inferred)	http://segatalab.cibio.unitn.it/data/Pasolli_et_al.html
**32**	QinJ_2012__NOM001_NODE_179_length_28679_cov_2.87521	find gene functions from 54 unique genomic loci	n/a	No	Yes	n/a	n/a	d__Bacteria; p__Firmicutes_A; c__Clostridia; o__Oscillospirales; f__Acutalibacteraceae; g__CAG-217 (inferred)	http://segatalab.cibio.unitn.it/data/Pasolli_et_al.html
**33**	QinJ_2012__T2D-050_NODE_25_length_192521_cov_10.1129	find gene functions from 54 unique genomic loci	n/a	No	Yes	n/a	n/a	d__Bacteria; p__Firmicutes_A; c__Clostridia; o__Oscillospirales; f__Acutalibacteraceae; g__CAG-217 (inferred)	http://segatalab.cibio.unitn.it/data/Pasolli_et_al.html
**34**	VatanenT_2016__G78791_NODE_43_length_22491_cov_6.98654	find gene functions from 54 unique genomic loci	n/a	No	Yes	n/a	n/a	d__Bacteria; p__Firmicutes_A; c__Clostridia; o__Oscillospirales; f__Acutalibacteraceae; g__CAG-217 (inferred)	http://segatalab.cibio.unitn.it/data/Pasolli_et_al.html
**35**	XieH_2016__YSZC12003_35392_NODE_87_length_196476_cov_13.3023	find gene functions from 54 unique genomic loci	n/a	No	Yes	n/a	n/a	d__Bacteria; p__Firmicutes_A; c__Clostridia; o__Oscillospirales; f__Acutalibacteraceae; g__CAG-217 (inferred)	http://segatalab.cibio.unitn.it/data/Pasolli_et_al.html
**36**	XieH_2016__YSZC12003_35563_NODE_11_length_353850_cov_18.1068	Fig 2, find gene functions from 54 unique genomic loci	n/a	No	Yes	n/a	n/a	d__Bacteria; p__Firmicutes_A; c__Clostridia; o__Oscillospirales; f__Acutalibacteraceae; g__CAG-217 (inferred)	http://segatalab.cibio.unitn.it/data/Pasolli_et_al.html
**37**	XieH_2016__YSZC12003_36005_NODE_238_length_99923_cov_5.91259	Fig 2, find gene functions from 54 unique genomic loci, source of orf1-encoding phage genome; Fig 3 Acr Seq	n/a	No	Yes	n/a	n/a	d__Bacteria; p__Firmicutes_A; c__Clostridia; o__Oscillospirales; f__Acutalibacteraceae; g__CAG-217 (inferred)	http://segatalab.cibio.unitn.it/data/Pasolli_et_al.html
**38**	XieH_2016__YSZC12003_36794_NODE_1_length_781521_cov_10.2961	find gene functions from 54 unique genomic loci, source of orf1-encoding phage genome; Fig 3 Acr Seq	n/a	No	Yes	n/a	n/a	d__Bacteria; p__Firmicutes_A; c__Clostridia; o__Oscillospirales; f__Acutalibacteraceae; g__CAG-217 (inferred)	http://segatalab.cibio.unitn.it/data/Pasolli_et_al.html
**39**	XieH_2016__YSZC12003_37133_NODE_3_length_676817_cov_24.9073	find gene functions from 54 unique genomic loci	n/a	No	Yes	n/a	n/a	d__Bacteria; p__Firmicutes_A; c__Clostridia; o__Oscillospirales; f__Acutalibacteraceae; g__CAG-217 (inferred)	http://segatalab.cibio.unitn.it/data/Pasolli_et_al.html
**40**	XieH_2016__YSZC12003_37322_NODE_5_length_601737_cov_115.712	find gene functions from 54 unique genomic loci	n/a	No	Yes	n/a	n/a	d__Bacteria; p__Firmicutes_A; c__Clostridia; o__Oscillospirales; f__Acutalibacteraceae; g__CAG-217 (inferred)	http://segatalab.cibio.unitn.it/data/Pasolli_et_al.html
**41**	XieH_2016__YSZC12003_37399_NODE_3_length_598430_cov_49.9887	find gene functions from 54 unique genomic loci	n/a	No	Yes	n/a	n/a	d__Bacteria; p__Firmicutes_A; c__Clostridia; o__Oscillospirales; f__Acutalibacteraceae; g__CAG-217 (inferred)	http://segatalab.cibio.unitn.it/data/Pasolli_et_al.html
**42**	XieH_2016__YSZC12003_37878_NODE_8_length_402183_cov_76.149	find gene functions from 54 unique genomic loci	n/a	No	Yes	n/a	n/a	d__Bacteria; p__Firmicutes_A; c__Clostridia; o__Oscillospirales; f__Acutalibacteraceae; g__CAG-217 (inferred)	http://segatalab.cibio.unitn.it/data/Pasolli_et_al.html
**43**	YuJ_2015__SZAXPI003435-11_NODE_1_length_772218_cov_11.5924	find gene functions from 54 unique genomic loci	n/a	No	Yes	n/a	n/a	d__Bacteria; p__Firmicutes_A; c__Clostridia; o__Oscillospirales; f__Acutalibacteraceae; g__CAG-217 (inferred)	http://segatalab.cibio.unitn.it/data/Pasolli_et_al.html
**44**	YuJ_2015__SZAXPI015230-16_NODE_32_length_174349_cov_17.3543	find gene functions from 54 unique genomic loci	n/a	No	Yes	n/a	n/a	d__Bacteria; p__Firmicutes_A; c__Clostridia; o__Oscillospirales; f__Acutalibacteraceae; g__CAG-217 (inferred)	http://segatalab.cibio.unitn.it/data/Pasolli_et_al.html
**45**	ZeeviD_2015__PNP_DietIntervention_11_NODE_16_le	find gene functions from 54 unique genomic loci	n/a	No	Yes	n/a	n/a	d__Bacteria; p__Firmicutes_A; c__Clostridia; o__Oscillospirales; f__Acutalibacteraceae; g__CAG-217 (inferred)	http://segatalab.cibio.unitn.it/data/Pasolli_et_al.html

	ngth_97163_cov_10.0000_ID_22997								
**46**	ZeeviD_2015__PNP_Main_234_NODE_10_length_202229_cov_13.9987_ID_180729	find gene functions from 54 unique genomic loci	n/a	No	Yes	n/a	n/a	d__Bacteria; p__Firmicutes_A; c__Clostridia; o__Oscillospirales; f__Acutalibacteraceae; g__CAG-217 (inferred)	http://segatalab.cibio.unitn.it/data/Pasolli_et_al.html
**47**	ZeeviD_2015__PNP_Main_294_NODE_20_length_208110_cov_20.9981_ID_106095	find gene functions from 54 unique genomic loci	n/a	No	Yes	n/a	n/a	d__Bacteria; p__Firmicutes_A; c__Clostridia; o__Oscillospirales; f__Acutalibacteraceae; g__CAG-217 (inferred)	http://segatalab.cibio.unitn.it/data/Pasolli_et_al.html
**48**	ZeeviD_2015__PNP_Main_390_NODE_33_length_137723_cov_10.9985_ID_46475	find gene functions from 54 unique genomic loci	n/a	No	Yes	n/a	n/a	d__Bacteria; p__Firmicutes_A; c__Clostridia; o__Oscillospirales; f__Acutalibacteraceae; g__CAG-217 (inferred)	http://segatalab.cibio.unitn.it/data/Pasolli_et_al.html
**49**	ZeeviD_2015__PNP_Main_578_NODE_20_length_138741_cov_8.9988_ID_132563	find gene functions from 54 unique genomic loci	n/a	No	Yes	n/a	n/a	d__Bacteria; p__Firmicutes_A; c__Clostridia; o__Oscillospirales; f__Acutalibacteraceae; g__CAG-217 (inferred)	http://segatalab.cibio.unitn.it/data/Pasolli_et_al.html
**50**	ZeeviD_2015__PNP_Main_741_NODE_13_length_214417_cov_12.0572_ID_91679	find gene functions from 54 unique genomic loci	n/a	No	Yes	n/a	n/a	d__Bacteria; p__Firmicutes_A; c__Clostridia; o__Oscillospirales; f__Acutalibacteraceae; g__CAG-217 (inferred)	http://segatalab.cibio.unitn.it/data/Pasolli_et_al.html
**51**	ZellerG_2014__CCIS03857607ST-4-0_NODE_542_length_35291_cov_2.7674	find gene functions from 54 unique genomic loci	n/a	No	Yes	n/a	n/a	d__Bacteria; p__Firmicutes_A; c__Clostridia; o__Oscillospirales; f__Acutalibacteraceae; g__CAG-217 (inferred)	http://segatalab.cibio.unitn.it/data/Pasolli_et_al.html
**52**	ZellerG_2014__CCIS22958137ST-20-0_NODE_40_length_181493_cov_7.91373	find gene functions from 54 unique genomic loci, source of orf1-encoding phage genome; Fig 3 Acr Seq	n/a	No	Yes	n/a	n/a	d__Bacteria; p__Firmicutes_A; c__Clostridia; o__Oscillospirales; f__Acutalibacteraceae; g__CAG-217 (inferred)	http://segatalab.cibio.unitn.it/data/Pasolli_et_al.html
**53**	XieH_2016__YSZC12003_35635_NODE_109_length_156568_cov_5.12141	find gene functions from 54 unique genomic loci	n/a	No	Yes	n/a	n/a	d__Bacteria; p__Firmicutes_A; c__Clostridia; o__Oscillospirales; f__Acutalibacteraceae; g__CAG-217 (inferred)	http://segatalab.cibio.unitn.it/data/Pasolli_et_al.html
**54**	ZeeviD_2015__PNP_Main_85_NODE_182_length_52997_cov_7.0000_ID_133080	find gene functions from 54 unique genomic loci	n/a	No	Yes	n/a	n/a	d__Bacteria; p__Firmicutes_A; c__Clostridia; o__Oscillospirales; f__Acutalibacteraceae; g__CAG-217 (inferred)	http://segatalab.cibio.unitn.it/data/Pasolli_et_al.html
**55**	4303_HeQ_2017__SZAXPI029570-85__bin.1_NODE_2_length_608092_cov_26.3259	Fig 2	n/a	Yes	No	HeQ_2017__SZAXPI029570-85__bin.1	4303	d__Bacteria; p__Firmicutes_A; c__Clostridia; o__Oscillospirales; f__Acutalibacteraceae; g__CAG-217	http://segatalab.cibio.unitn.it/data/Pasolli_et_al.html
**56**	4303_CosteaPI_2017__SID713B074-11-90-0__bin.57_NODE_18_length_238289_cov_5.37382	Source of orf1-encoding phage genome; Fig 3 Acr Seq	n/a	Yes	No	CosteaPI_2017__SID713B074-11-90-0__bin.57	4303	d__Bacteria; p__Firmicutes_A; c__Clostridia; o__Oscillospirales; f__Acutalibacteraceae; g__CAG-217	http://segatalab.cibio.unitn.it/data/Pasolli_et_al.html
**57**	Clostridiales_bacterium_isolate_CIM:MAG_317_1 contig_8085	Fig 2, source of orf1-encoding phage genome	QALM01000002.1	No	No	n/a	n/a	d__Bacteria; p__Firmicutes_A; c__Clostridia; o__Oscillospirales; f__Acutalibacteraceae; g__CAG-217 (inferred)	NCBI Genbank
**58**	TPA_asm:_Ruminococcaceae_bacterium_isolate_UBA8277_contig_226	Fig 3 Acr Seq	DPDR01000010.1	No	No	n/a	n/a	d__Bacteria; p__Firmicutes_A; c__Clostridia; o__Oscillospirales; f__Acutalibacteraceae; g__CAG-217 (inferred)	NCBI Genbank
**59**	KarlssonFH_2013__S424_NODE_2_length_526279_cov_9.22761	Source of orf1-encoding phage genome	n/a	No	Yes	n/a	n/a	d__Bacteria; p__Firmicutes_A; c__Clostridia; o__Oscillospirales; f__Acutalibacteraceae; g__CAG-217 (inferred)	http://segatalab.cibio.unitn.it/data/Pasolli_et_al.html

**60**	XieH_2016__YSZC12003_36696_NODE_1_length_776477_cov_39.8546	Source of orf1-encoding phage genome, AcrIIA17 encoding phage (S5 Fig)	n/a	No	Yes	n/a	n/a	d__Bacteria; p__Firmicutes_A; c__Clostridia; o__Oscillospirales; f__Acutalibacteraceae; g__CAG-217 (inferred)	http://segatalab.cibio.unitn.it/data/Pasolli_et_al.html
**61**	XieH_2016__YSZC12003_37308R1_NODE_3_length_717276_cov_26.9646	source of orf1-encoding phage genome	n/a	No	Yes	n/a	n/a	d__Bacteria; p__Firmicutes_A; c__Clostridia; o__Oscillospirales; f__Acutalibacteraceae; g__CAG-217 (inferred)	http://segatalab.cibio.unitn.it/data/Pasolli_et_al.html
**62**	ZellerG_2014__CCIS88007743ST-4-0_NODE_31_length_210910_cov_8.07406	source of orf1-encoding phage genome	n/a	No	Yes	n/a	n/a	d__Bacteria; p__Firmicutes_A; c__Clostridia; o__Oscillospirales; f__Acutalibacteraceae; g__CAG-217 (inferred)	http://segatalab.cibio.unitn.it/data/Pasolli_et_al.html
**63**	4303_QinN_2014__LD-22__bin.75_NODE_22_length_329763_cov_10.7401	Fig 3 Acr Seq	n/a	Yes	No	QinN_2014__LD-22__bin.75	4303	d__Bacteria; p__Firmicutes_A; c__Clostridia; o__Oscillospirales; f__Acutalibacteraceae; g__CAG-217	http://segatalab.cibio.unitn.it/data/Pasolli_et_al.html
**64**	ZellerG_2014__CCMD25963797ST-21-0_NODE_9_length_356111_cov_10.1715	Fig 3 Acr Seq	n/a	No	Yes	n/a	n/a	d__Bacteria; p__Firmicutes_A; c__Clostridia; o__Oscillospirales; f__Acutalibacteraceae; g__CAG-217 (inferred)	http://segatalab.cibio.unitn.it/data/Pasolli_et_al.html
**65**	ZellerG_2014__CCIS41222843ST-4-0_NODE_17_length_267133_cov_14.7383	Fig 3 Acr Seq	n/a	No	Yes	n/a	n/a	d__Bacteria; p__Firmicutes_A; c__Clostridia; o__Oscillospirales; f__Acutalibacteraceae; g__CAG-217 (inferred)	http://segatalab.cibio.unitn.it/data/Pasolli_et_al.html
**66**	FengQ_2015__SID530168_NODE_20_length_224404_cov_6.02914	Fig 3 Acr Seq	n/a	No	Yes	n/a	n/a	d__Bacteria; p__Firmicutes_A; c__Clostridia; o__Oscillospirales; f__Acutalibacteraceae; g__CAG-217 (inferred)	http://segatalab.cibio.unitn.it/data/Pasolli_et_al.html
**67**	FengQ_2015__SID530041_NODE_7_length_421742_cov_9.32571	Fig 3 Acr Seq	n/a	No	Yes	n/a	n/a	d__Bacteria; p__Firmicutes_A; c__Clostridia; o__Oscillospirales; f__Acutalibacteraceae; g__CAG-217 (inferred)	http://segatalab.cibio.unitn.it/data/Pasolli_et_al.html
**68**	FengQ_2015__SID31223_NODE_13_length_228767_cov_7.50553	Fig 3 Acr Seq	n/a	No	Yes	n/a	n/a	d__Bacteria; p__Firmicutes_A; c__Clostridia; o__Oscillospirales; f__Acutalibacteraceae; g__CAG-217 (inferred)	http://segatalab.cibio.unitn.it/data/Pasolli_et_al.html

Reference for S3 Table.

Pasolli, E., Asnicar, F., Manara, S., Zolfo, M., Karcher, N., Armanini, F., Beghini, F., Manghi, P., Tett, A., Ghensi, P., *et al.* (2019). Extensive Unexplored Human Microbiome Diversity Revealed by Over 150,000 Genomes from Metagenomes Spanning Age, Geography, and Lifestyle. Cell *176*, 649-662 e620.
