# Supplementary material for: The novel anti-CRISPR AcrIIA22 relieves DNA torsion in target plasmids and impairs SpyCas9 activity
Source: PLoS Biol. 2021 Oct 13;19(10):e3001428. doi: 10.1371/journal.pbio.3001428 (PMC8545432; doi:10.1371/journal.pbio.3001428)
Supplement: S4 Table — (PDF) [file pbio.3001428.s015.pdf]

S4 Table. Plasmids used in this study. S5 Table indicates genes expressed from pZE21_tetR.

Plasmid	crRNA promoter, sequence (5'-3')	Notes	Refs	Purpose
pZE21_tetR	n/a	Contains tetR behind pLac promoter for inducible expression of candidate Acrs. Targeted by crRNA_A; PAM = AGG.	(Forsberg et al., 2019)	Expressing genes to test *in vivo* anti-CRISPR activity
pSpyCas9 _crA	pJ23100, GTTCATTCAGGGCAC CGGAC	Arabinose-inducible SpyCas9 with pZE21 targeting pZE21_tetR	(Forsberg et al., 2019)	Target pZE21_tetR for elimination with SpyCas9
pSpyCas9 _crMu	pJ23100, GTAATACTTGTCCCGC AAAG	Mu-targeting spacer for phage Mu immunity testing. Otherwise identical to pSpyCas9_crA	(Forsberg et al., 2019)	Phage Mu immunity testing
pSpyCa9_crNT	pJ23100, GAACGAAAAGCTGCG CCGGG	non-targeting spacer used as control. Otherwise identical to pSpyCas9_crA	(Forsberg et al., 2019)	Phage Mu immunity testing, Western blots
pCloDF13_GFP	pJ23100, GAACGAAAAGCTGCG CCGGG	*eGFP* gene replaces *spyCas9* in pSpyCas9_crA		Measure generic protein expression from pSpyCas9 expression vector
pIDTsmart	n/a	Plasmid used for *in-vitro* SpyCas9 digestion. Sequence available at: https://www.idtdna.com/pages/products/genes-and-gene-fragments/custom-gene-synthesis		Plasmid template for *in-vitro* nuclease reactions
pET15b/HE	n/a	Novagen Cat. No. 69661-3; pET15 variants 'b' and 'HE' differ only by a few bases upstream of the N-terminal thrombin cut site		Protein purification
pSpyCas9_Fig3C	n/a	J23100 promoter expressing a theophylline inducible SpyCas9, used in Fig 3C	(Uribe et al., 2019)	Testing AcrIIA22 activity against a panel of Cas9 and Cas12 effector nucleases
pNmCas9_Fig3C	n/a	J23100 promoter expressing a theophylline inducible NmCas9, used in Fig 3C	(Uribe et al., 2019)	Testing AcrIIA22 activity against a panel of Cas9 and Cas12 effector nucleases
pLbCas12_Fig3C	n/a	J23100 promoter expressing a theophylline inducible LbCas12, used in Fig 3C	(Uribe et al., 2019)	Testing AcrIIA22 activity against a panel of Cas9 and Cas12 effector nucleases
pFnCas12_Fig3C	n/a	J23100 promoter expressing a theophylline inducible FnCas12, used in Fig 3C	(Uribe et al., 2019)	Testing AcrIIA22 activity against a panel of Cas9 and Cas12 effector nucleases
pDual4_Spy	P_{BAD}, GTTCATTCAGGGCAC CGGAC	Arabinose inducible gRNA for SpyCas9 targeting pZE21	(Uribe et al., 2019)	Testing AcrIIA22 activity against a panel of Cas9 and Cas12 effector nucleases
pDual4_Nm	P_{BAD}, GAACACGGCGGCATC AGAGC	Arabinose inducible gRNA for NmCas9 targeting pZE21	(Uribe et al., 2019)	Testing AcrIIA22 activity against a panel of Cas9 and Cas12 effector nucleases
pDual4_Lb	P_{BAD}, TCAAGACCGACCTGT CCGGTGCCCTGAATG	Arabinose inducible gRNA for LbCas12 targeting pZE21	(Uribe et al., 2019)	Testing AcrIIA22 activity against a panel of Cas9 and Cas12 effector nucleases
pDual4_Fn	P_{BAD}, TCAAGACCGACCTGT CCGGTGCCCTGAATG	Arabinose inducible gRNA for FnCas12 targeting pZE21	(Uribe et al., 2019)	Testing AcrIIA22 activity against a panel of Cas9 and Cas12 effector nucleases

References for S4 Table.

Forsberg, K.J., Bhatt, I.V., Schmidtke, D.T., Javanmardi, K., Dillard, K.E., Stoddard, B.L., Finkelstein, I.J., Kaiser, B.K., and Malik, H.S. (2019). Functional metagenomics-guided discovery of potent Cas9 inhibitors in the human microbiome. Elife *8*.

Uribe, R.V., van der Helm, E., Misiakou, M.A., Lee, S.W., Kol, S., and Sommer, M.O.A. (2019). Discovery and Characterization of Cas9 Inhibitors Disseminated across Seven Bacterial Phyla. Cell Host Microbe *25*, 233-241 e235.
