# Supplementary material for: The novel anti-CRISPR AcrIIA22 relieves DNA torsion in target plasmids and impairs SpyCas9 activity
Source: PLoS Biol. 2021 Oct 13;19(10):e3001428. doi: 10.1371/journal.pbio.3001428 (PMC8545432; doi:10.1371/journal.pbio.3001428)
Supplement: S5 Table — (PDF) [file pbio.3001428.s016.pdf]

S5 Table. Gene sequences used in this study.

Gene Name	Sequence	Notes
acrIIA22wt	atggtagtagaagagacgcgggatttagccgaaactgcggattgtgtagtgatcgaagccattttagtggatgacggattgcgttacagacagctttctgtcggcatcaaagacgaaaacggcgacattattcgtatcgtccctatttcaaccgtt*ctgatc*tag	The italicized six base pairs were deleted in the ∆2aa truncation mutant via Q5 site-directed mutagenesis (NEB).
acrIIA22-null	atggtagtagaagagacgcgggatttagccgaaactgcggattgtgtagtgatcgaagccattt**A**agtggatgacggattgcgttacagacagctttctgtcggcatcaaagacgaaaacggcgacattattcgtatcgtccctatttcaaccgttctgatctag	Mutation to introduce early stop codon via Q5 site-directed mutagenesis (NEB). Indicated in bold, capitalized, underline
acrIIA22a	atggtcatagaagagacgcgggatttagctgaaactgcggattgtgtagtgatcgaagccattttagtggatgacggattgcgttacaaacagctttccgtcggcatcaaagacgaaaacggtgacattattcgtatcgtccctatttcaaccgttctgatctag	Same amino acid sequence as NCBI protein CDB51368.1; synthesized by GenScript and cloned into pZE21_tetR
acrIIA22b	atgattgtggaagataccaaagatttggttgaaactgcggactatgtgatcatcgaagctgttttagtggatgatggattgcgttacaaacaactttctgttggcattaaagccaaaaatggtgacattatccgcataattccaatatcgacaatgctgatgtaa	Synthesized by GenScript and cloned into pZE21_tetR
acrIIA22c	atgaaaatgattgtggaagatacgaaagatctggtagaaacggacgattatgtaatcattgaagcgactttgtcagagggcgatttgttgtttgtgcaaattgccgtgggcattcgcaacgaagtgggcgacattgttcgtattattcccatttccaccaacccaatctaa	Same amino acid sequence as NCBI protein CDB51757.1; synthesized by GenScript and cloned into pZE21_tetR
ts-acrIIA22	atg***tggagtcatccacaatttgagaag****ggaggaggcagtggaggaggcag****tggaggaagtgcctggagccacccgcagttcgaaaaa****ggcagtggtggtggtagtggtggagga*atggtagtagaagagacgcgggatttagccgaaactgcggattgtgtagtgatcgaagccattttagtggatgacggattgcgttacagacagctttctgtcggcatcaaagacgaaaacggcgacattattcgtatcgtccctatttcaaccgttctgatctag	N-terminal twin-strep (ts) tagged AcrIIA22. The tag is indicated in bold italics, linker regions are only italicized; synthesized by GenScript and cloned into pZE21_tetR for functional testing.
acrIIA22-ts	atggtagtagaagagacgcgggatttagccgaaactgcggattgtgtagtgatcgaagccattttagtggatgacggattgcgttacagacagctttctgtcggcatcaaagacgaaaacggcgacattattcgtatcgtccctatttcaaccgttctgatc*ggaggaggcagtggaggaggcagtggaggaagtgcc****tggagtcatccacaatttgagaag****ggaggaggcagtggaggaggcagtggaggaagtgcc****tggagccacccgcagttcgaaaaa***tag	C-terminal twin-strep (ts) tagged AcrIIA22. The tag is indicated in bold italics, linker regions are only italicized; synthesized by GenScript and cloned into pZE21_tetR or pET15 for functional testing or protein purification, respectively.
purF	atgttcgatagtttgcacgaggaatgcggtgttttcggcgtatttgaaaatcagaccactacggtggcccagacggcgtatctggctctgtttgccttgcagcacagagggcaggagagttgcggcattgccgtgaatgacgacggcgtgtttcgccaccatcggggcgacggactggtgccggatgtgtttagcaaggagcagctggctgccctgggtacaggtaatatggccatcggtcatgtgcgctactccaccaccggcggcaaaaacgccaacaatattcagcccctggtcattcgccatattaagggtaatttggcggtggcacataacggcaatttggtaaacgccccggagctgcgccgccagtttgagctgaagggcgccatttttcacggcacatcggacaccgagtccattgcctattctattgtagaggagcgcctgcacagtaagagcacggaagaggccatcgaaaaaatcatgccccggctgcaaggggcattctcttgcgtggtgatgactgccaccaaactcattgcgtttcgtgaccccaacggctttcggcctctttgcctgggtaagactgcggacgatgcttatgtggtggcgtcggagagttgtgcgctggattccatcggcgcccactttgtgcggaatattgctcccggcgagatcgttgtgatcagcaaggatggcgtgcgctctattaccacccattgcggcggactacgccacatttgtgtgtttgagtacatctattttgctcggccggacagtgtgattgagggcgtgtctgtgcagcacgccagaatgcgggccggtgcgtacctggcgaaggaacacccggtagacgcggatattgtcatcggcgtgccggacagcggcctggacgccgccttgggctatgcccaggagagcggcattccttacggtattggatttatcaagaaccgctacatcggccgcagctttattcagcctacccaaggtcagcgtgaggacgcggtgaagatcaagctgaatgtactgcgagagaatatcaagggcaagcgggtggtgatgatcgatgactctattgttcgcggcaccaccagcgctcggattgtcagcctgctgcgagaggccggcgccaccgaggtgcatatgcgggtttctgcccctccgtttcggcatccttgcttctttggaacggacattgatagcgaagaaaacctgattgcatgcaaatttcacgaaatttctgaaatttcttgccaattaggggttgacagcttggggtatcttagtgtaaaatctactcacgaacttgcgaaggagtccggattcgatttttgcgacggttgcttcaccggccattatcccatccccaccccgaagcaacagtccaaggataagtttgaggaaaagctgaatcagttctcctcttactaccaggtcttggattaa	Flanks *acrIIA22*-encoding bacterial genomic islands. Used as bait to retrieve additional examples of this locus for genomic and evolutionary analyses.
radC	atgcgtgccgcttatctgcaaggcggcggcgacgctatgccggaccaccagttgctggaattgctgctgtccatcagcattccccgcagagatgtaaagcccattgcctatgcgctcattaaccgcttcggctcgctggagcaggtgtttgccgccggcgcagcagatctgcaacaagtgccgggcgtcggcgaacagaccgccgtacagattctgctggtacgggatctgaaccggcggatccatcaaaatcaaaacaaaccggtcaagcacctgacagatgccacccagtcctgcgcctacttttccaatctgttacgggacaaaaccgccgagcaggtgtacttggtcaccctggacggcagtgccaaaatcctgcaaacccacgccgtaggcagcggcagcgtcaacctggcctctgtggatcagcgcactttgatggaacatattctgcgagacaacgccaacgctgttatgctggcacacaaccatcccggcggcaaggcccagccctctgcgcaggatctggaattcaccattcgtctgctttccattctgcgttccattcatgtgcagctgctggatcatattatcgtcagtcctaccgacacctactccatgcgcagcgacccggagtacggcagcttcttcaccgtcaaataa	Flanks *acrIIA22*-encoding bacterial genomic islands. Used as bait to retrieve additional examples of this locus for genomic and evolutionary analyses.
acrIIA4	atgaatattaacgatttgatccgtgagattaagaataaggattatactgtcaaattgtccgggacagattccaattctattacacaattaatcatccgtgtgaataacgatggtaatgagtatgtcatctctgaatcagaaaacgagagcatcgtagaaaagttcatcagtgccttcaagaacgggtggaaccaagagtatgaagatgaggaggaattttacaatgatatgcagacaattacgcttaaatcagaattgaattaa	Discovered by (Rauch et al., 2017); synthesized by GenScript and cloned into pZE21_tetR
spyCas9	atggataagaaatactcaataggcttagatatcggcacaaatagcgtcggatgggcggtgatcactgatgaatataaggttccgtctaaaaagttcaaggttctgggaaatacagaccgccacagtatcaaaaaaaatcttataggggctcttttatttgacagtggagagacagcggaagcgactcgtctcaaacggacagctcgtagaaggtatacacgtcggaagaatcgtatttgttatctacaggagattttttcaaatgagatggcgaaagtagatgatagtttctttcatcgacttgaagagtcttttttggtggaagaagacaagaagcatgaacgtcatcctatttttggaaatatagtagatgaagttgcttatcatgagaaatatccaactatctatcatctgcgaaaaaaattggtagattctactgataaagcggatttgcgcttaatctatttggccttagcgcatatgattaagtttcgtggtcatttttgattgagggagatttaaatcctgataatagtgatgtggacaaactatttatccagttggtacaaacctac	The sequence was amplified from Addgene plasmid #48645 (Esvelt *et al.*, 2013) for use in this study as described in (Forsberg *et al.*, 2019).

	aatcaattatttgaagaaaccctattaacgcaagtggagtagatgctaaagcgattctttctgcacgattgagtaaatcaagacgattagaaaatctcattgctcagctcccc<br>ggtgagaagaaaaatggcttatttgggaatctcattgctttgtcattgggtttgacccctaattttaaatcaaattttgatttggcagaagatgctaaattacagctttcaaaagata<br>cttacgatgatgatttagataatttattggcgcaaattggagatcaatatgctgatttgtttttggcagctaagaatttatcagatgctattttactttcagatatcctaagagtaaatac<br>tgaaataactaaggctcccctatcagcttcaatgattaaacgctacgatgaacatcatcaagacttgactcttttaaaagctttagttcgacaacaacttccagaaaagtataa<br>agaaatctttttgatcaatcaaaaaacggatatgcaggttatattgatgggggagctagccaagaagaattttataaatttatcaaaccaattttagaaaaaatggatggtact<br>gaggaattattggtgaaactaaatcgtgaagatttgctgcgcaagcaacggacctttgacaacggctctattccccatcaaattcacttgggtgagctgcatgctattttgagaa<br>gacaagaagacttttatccatttttaaaagacaatcgtgagaagattgaaaaaatcttgacttttcgaattccttattatgttggtccattggcgcgtggcaatagtcgttttgcatgg<br>atgactcggaagtctgaagaaacaattaccccatggaattttgaagaagttgtcgataaaggtgcttcagctcaatcatttattgaacgcatgacaaactttgataaaaatcttc<br>caaatgaaaaagtactaccaaaacatagtttgctttatgagtattttacggtttataacgaattgacaaaggtcaaatatgttactgaaggaatgcgaaaaccagcatttctttc<br>aggtgaacagaagaaagccattgttgatttactcttcaaaacaaatcgaaaagtaaccgttaagcaattaaaagaagattatttcaaaaaaatagaatgttttgatagtgttg<br>aaatttcaggagttgaagatagatttaatgcttcattaggtacctaccatgatttgctaaaaattattaaagataaagatttttggataatgaagaaaatgaagatatcttagagg<br>atattgttttaacattgaccttatttgaagatagggagatgattgaggaaagacttaaaacatatgctcacctctttgatgataaggtgatgaaacagcttaaacgtcgccgttat<br>actggttggggacgtttgtctcgaaaattgattaatggtattagggataagcaatctggcaaaacaatattagatttttgaaatcagatggttttgccaatcgcaattttatgcagc<br>tgatccatgatgatagtttgacatttaaagaagacattcaaaaagcacaagtgtctggacaaggcgatagtttacatgaacatattgcaaatttagctggtagccctgctattaa<br>aaaaggtattttacagactgtaaaagttgttgatgaattggtcaaagtaatggggcggcataagccagaaaatatcgttattgaaatggcacgtgaaaatcagacaactcaa<br>aagggccagaaaaattcgcgagagcgtatgaaacgaatcgaagaaggtatcaaagaattaggaagtcagattcttaaagagcatcctgttgaaaatactcaattgcaaa<br>atgaaaagctctatctctattatctccaaaatggaagagacatgtatgtggaccaagaattagatattaatcgtttaagtgattatgatgtcgatcacattgttccacaaagtttcct<br>taaagacgattcaatagacaataaggtcttaacgcgttctgataaaaatcgtggtaaatcggataacgttccaagtgaagaagtagtcaaaaagatgaaaaactattggag<br>acaacttctaaacgccaagttaatcactcaacgtaagtttgataatttaacgaaagctgaacgtggaggtttgagtgaacttgataaagctggttttatcaaacgccaattggtt<br>gaaactcgccaaatcactaagcatgtggcacaaattttggatagtcgcatgaatactaaatacgatgaaaatgataaacttattcgagaggttaaagtgattaccttaaaatct<br>aaattagtttctgacttccgaaaagatttccaattctataaagtacgtgagattaacaattaccatcatgcccatgatgcgtatctaaatgccgtcgttggaactgctttgattaag<br>aaatatccaaaacttgaatcggagtttgtctatggtgattataaagtttatgatgttcgtaaaatgattgctaagtctgagcaagaaataggcaaagcaaccgcaaaatatttctt<br>ttactctaatatcatgaacttcttcaaaacagaaattacacttgcaaatggagagattcgcaaacgccctctaatcgaaactaatggggaaactggagaaattgtctgggata<br>aagggcgagattttgccacagtgcgcaaagtattgtccatgccccaagtcaatattgtcaagaaaacagaagtacagacaggcggattctccaaggagtcaattttaccaa<br>aaagaaattcggacaagcttattgctcgtaaaaaagactgggatccaaaaaaatatggtggttttgatagtccaacggtagcttattcagtcctagtggttgctaaggtggaa<br>aaagggaaatcgaagaagttaaaatccgttaaagagttactagggatcacaattatggaaagaagttcctttgaaaaaaatccgattgactttttagaagctaaaggatata<br>aggaagttaaaaaagacttaatcattaaactacctaaatatagtctttttgagttagaaaacggtcgtaaacggatgctggctagtgccggagaattacaaaaaggaaatga<br>gctggctctgccaagcaaatatgtgaatttttatatttagctagtcattatgaaaagttgaagggtagtccagaagataacgaacaaaaacaattgtttgtggagcagcataa<br>gcattatttagatgagattattgagcaaatcagtgaattttctaagcgtgttattttagcagatgccaatttagataaagttcttagtgcatataacaaacatagagacaaaccaat<br>acgtgaacaagcagaaaatattattcatttatttacgttgacgaatcttggagctcccgctgcttttaaatattttgatacaacaattgatcgtaaacgatatacgtctacaaaaga<br>agttttagatgccactcttatccatcaatccatcactggtctttatgaaacacgcattgatttgagtcagctaggaggtgactga	
nmCas9	atggccgccttcaagcccaaccccatcaactacatcctgggcctggacatcggcatcgccagcgtgggctgggccatggtggagatcgacgaggacgagaaccccatc<br>tgcctgatcgacctgggtgtgcgcgtgttcgagcgcgctgaggtgcccaagactggtgacagtctggctatggctcgccggcttgctcgctctgttcggcgccttactcgccgg<br>cgcgctcaccgccttctgcgcgctcgccgcctgctgaagcgcgagggtgtgctgcaggctgccgacttcgacgagaacggcctgatcaagagcctgcccaacactccttg<br>gcagctgcgcgctgccgctctggaccgcaagctgactcctctggagtggagcgccgtgctgctgcacctgatcaagcaccgcggctacctgagccagcgcaagaacga<br>gggcgagaccgccgacaaggagctgggtgctctgctgaagggcgtggccgacaacgcccacgccctgcagactggtgacttccgcactcctgctgagctggccctgaa<br>caagttcgagaaggagagcggccacatccgcaaccagcgcggcgactacagccacaccttcagccgcaaggacctgcaggccgagctgatcctgctgttcgagaag<br>cagaaggagttcggcaaccccccacgtgagcggcggcctgaaggagggcatcgagaccctgctgatgacccagcgccccgccctgagcggcgacgccgtgcagaag<br>atgctgggccactgcaccttcgagccagccgagcccaaggccgccaagaacacctacaccgccgagcgcttcatctggctgaccaagctgaacaacctgcgcatcctg<br>gagcagggcagcgagcgcccccctgaccgacaccgagcgcgccaccctgatggacgagccctaccgcaagagcaagctgacctacgcccaggcccgcaagctgct<br>gggtctggaggacaccgccttcttcaagggcctgcgctacggcaaggacaacgccgaggccagcaccctgatggagatgaaggcctaccacgccatcagccgcgccc<br>tggagaaggagggcctgaaggacaagaagagtcctctgaacctgagccccgagctgcaggacgagatcggcaccgccttcagcctgttcaagaccgacgaggacat<br>caccggccgcctgaaggaccgcatccagcccgagatcctggaggccctgctgaagcacatcagcttcgacaagttcgtgcagatcagcctgaaggccctgcgccgcat<br>cgtgcccctgatggagcagggcaagcgctacgacgaggcctgcgccgagatctacggcgaccactacggcaagaagaacaccgaggagaagatctacctgcctcct<br>atccccgccgacgagatccgcaaccccgtggtgctgcgcgccctgagccaggcccgcaaggtgatcaacggcgtggtgcgccgctacggcagccccgcccgcatcca<br>catcgagaccgcccgcgaggtgggcaagagcttcaaggaccgcaaggagatcgagaagcgccaggaggagaaccgcaaggaccgcgagaaggccgccgccaa<br>gttccgcgagtacttccccaacttcgtgggcgagcccaagagcaaggacatcctgaagctgcgcctgtacgagcagcagcacggcaagtgcctgtacagcggcaagga<br>gatcaacctgggccgcctgaacgagaagggctacgtggagatcgaccacgccctgcccttcagccgcacctgggacgacagcttcaacaacaaggtgctggtgctggg<br>cagcgagaaccagaacaagggcaaccagacccccctacgagtacttcaacggcaaggacaacagccgcgagtggcaggagttcaaggcccgcgtggagaccagcc<br>gcttcccccgcagcaagaagcagcgcatcctgctgcagaagttcgacgaggacggcttcaaggagcgcaacctgaacgacacccgctacgtgaaccgcttcctgtgcc<br>agttcgtggccgaccgcatgcgcctgaccggcaagggcaagaagcgcgtgttcgccagcaacggccagatcaccaacctgctgcgcggcttctggggcctgcgcaag<br>gtgcgcgccgagaacgaccgccaccacgccctggacgccgtggtggtggcctgcagcaccgtggccatgcagcagaagatcacccgcttcgtgcgctacaaggagat<br>gaacgccttcgacggtaaaaccatcgacaaggagaccggcgaggtgctgcaccagaagacccacttcccccagccctgggagttcttcgcccaggaggtgatgatccg<br>cgtgttcggcaagcccgacggcaagcccgagttcgaggaggccgacacccccgagaagctgcgcaccctgctggccgagaagctgagcagccgccctgaggccgtg<br>cacgagtacgtgactcctctgttcgtgagccgcgcccccaaccgcaagatgagcggtcagggtcacatggagaccgtgaagagcgccaagcgcctggacgagggcgt<br>gagcgtgctgcgcgtgcccctgacccagctgaagctgaaggacctggagaagatggtgaaccgcgagcgcgagcccaagctgtacgaggccctgaaggcccgcctg<br>gaggcccacaaggacgaccccgccaaggccttcgccgagcccttctacaagtacgacaaggccggcaaccgcacccagcaggtgaaggccgtgcgcgtggagcag<br>gtgcagaagaccggcgtgtgggtgcgcaaccacaacggcatcgccgacaacgccaccatggtgcgcgtggacgtgttcgagaagggcgacaagtactacctggtgcc	The sequence was amplified from Addgene plasmid #48646 (Esvelt *et al.*, 2013). The sequence of NmCas9 was cloned in place of the cas9 gene in pSpyCas9_Fig3C.

	catctacagctggcaggtggccaagggcatcctgcccgaccgcgccgtggtgcagggcaaggacgaggaggactggcagctgatcgacgacagcttcaacttcaagtt<br>cagcctgcaccccaacgacctggtggaggtgatcaccaagaaggcccgcatgttcggctacttcgccagctgccaccgcggcaccggcaacatcaacatccgcatcca<br>cgacctggaccacaagatcggcaagaacggcatcctggagggcatcggcgtgaagaccgccctgagcttccagaagtaccagatcgacgagctgggcaaggagatc<br>cgcccctgccgcctgaagaagcgccctcctgtgcgctaa	
lbCas12	atgtcaaagctcgagaaattcaccaactgttattcgttgagcaaaacactgcggtttaaagcgattccagtcggcaagactcaagagaatatagacaataagcggctgttg<br>gtggaagatgaaaagcgcgcggaagactacaaaggggtgaagaagttgttggacagatactacctctcttttatcaatgatgtcttgcactcaatcaaattgaagaatctga<br>acaactacatctccctcttcagaaagaaaacaaggacagaaaaggagaataaggaacttgaaaatttggagatcaatctgaggaaagagatcgcgaaagcctttaaag<br>gcaacgaaggatacaaaagtctgttcaagaaggatataattgagacaattttgccagagttcctcgatgacaaggacgagattgcgctggtcaattcgttcaacggattcac<br>aacagcattcacaggcttctttgataatcgggaaaatatgttctctgaggaggcaaagtccacttctattgcgttcaggtgtatcaatgagaatctcactaggtacatttccaaca<br>tggatatctttgagaaggttgacgcaatttttgacaagcacgaagttcaggagattaaggagaagatcctcaattccgattatgacgttgaggacttcttcgaaggtgagtttttt<br>aatttcgtgctcactcaagagggtatcgacgtgtataatgcgatcatcggtgggttcgtgactgagtccggtgaaaagattaagggattgaacgagtatatcaacctttacaac<br>caaaagacgaaacagaagctgccaaagttcaagcctctttacaaacaggttctttcagaccgcgagtcactctcgttctatggggagggctacacttcggatgaggaagtc<br>ctggaggtgttcaggaatactctcaataagaattcggagattttctcttctataaaaaaactggaaaagttgtttaagaattttgacgaatactctagcgccggcatatttgtgaa<br>aaacggcccggccatatcaacgataagtaaagatatcttcggcgaatggaacgtgatcagagacaaatggaacgcggagtatgacgatattcacctgaagaagaagg<br>ctgtcgtaacggagaagtacgaggatgatcgcaggaaaagcttcaaaaagatcggaagtttcagcctggaacagttgcaggagtatgctgacgccgatcttagcgtcgtc<br>gagaagttgaaggagataatcatccaaaaggtcgacgagatatataaagtctatggatcaagtgaaaaactgttcgacgccgacttcgttttggagaagtccctgaagaa<br>gaacgacgctgttgttgccattatgaaggatctgctcgacagcgtgaagagtttcgagaactatattaaggctttttttcggggaggggaaggagactaacagagatgagtcct<br>tctacggagacttcgtcctcgcgtacgatatactccttaaggtagaccacatctacgacgcaatcagaaattacgtgacacaaaagccgtacagcaaggacaagttcaaa<br>ctctacttccagaacccccagttcatgggcggctgggacaaggacaaggaaacggattacagggctacgatcctgaggtatggttcaaaatactacttggcgattatggac<br>aagaagtacgccaagtgtctccagaagattgacaaagacgatgtcaatggcaattatgagaagatcaactacaagctgcttccgggtccgaacaagatgctcccaaagg<br>ttttcttcagcaagaaatggatggcctactataacccaagcgaggacatccagaagatttataagaacggtacgttcaagaagggcgacatgttcaatcttaacgactgtca<br>caagctgatcgacttcttcaaagactcaattagccggtacccaaagtggtctaacgcctatgacttcaacttttcggaaaccgagaagtacaaggatatagccggattttata<br>gagaggtggaagagcagggctacaaggtgtcattcgagtccgccagcaagaaggaagtggacaagctcgtggaagagggtaagctctacatgttccagatttataataa<br>agactttagcgataagagccacgggacacctaatctccacacaatgtatttcaagctgctcttcgacgagaataaccacggccaaatcaggttgtcaggaggggctgaact<br>cttcatgcggcgcgctagccttaagaaggaggagcttgtagtccaccctgcgaatagtccaattgcgaataagaacccggacaatcctaaaaagactacaacattgagct<br>acgacgtgtacaaggataagaggttttccgaggatcagtacgagctccacatcccgattgcgatcaacaagtgcccaaagaatattttcaagataaacacagaggtgcgt<br>gtactcctgaagcatgacgacaatccttacgtcattgggattgatcggggcgagaggaacctcctctatattgtggtggtggacgggaaggggaacatagtcgaacagtac<br>tcccttaacgaaataattaacaatttcaacggcatccgtatcaagaccgactaccattcgttgctggacaagaaggagaaggagagatttgaggcgcggcaaaattggac<br>aagtatcgagaacatcaaggaactcaaagcaggttatatctctcaagttgtgcataagatatgcgagctggttgagaagtatgacgcagtgatcgctcttgaggacctcaac<br>tcgggctttaagaattctagagttaaagtggagaagcaggtctatcaaaagttcgagaagatgcttatagataagctcaactacatggtcgataagaaatcgaacccatgtg<br>ccaccggcggcgcactcaaaggttaccaaataacaaacaaattcgagtccttcaaatcgatgagtactcagaatgggttcatattttatataccggcgtggcttacgtctaag<br>atcgacccgtcaactggttttgtcaacctgttgaagacgaaatacacgtccattgccgattcgaaaaagttcatatctagttttgatcgtattatgtacgtcccagaggaagatctt<br>ttcgagtttgctctcgactacaaaaacttttcgcggaccgatgcggattacattaaaaaatggaaactctattcgtacggcaacagaatcaggatttttcgcaaccctaagaag<br>aataacgtctttgattgggaggaagtttgcttgactagcgcgtacaaggagctctttaataagtatggcattaactaccaacagggtgatatcagagcactgctttgcgaacaa<br>tctgacaaggctttctactcatccttcatggctttgatgagcctgatgctccagatgagaaattcaattacaggcagaaccgacgtggatttcttgatctccccggttaaaaattct<br>gatggcatcttttacgatagcaggaactatgaagcgcaagagaatgcgattctgccaaaaaaatgcagacgccaacggtgcctataacatcgccaggaaagtcctgtggg<br>cgatcggccagttcaaaaaggccgaagacgaaaaattggacaaggtcaaaatcgctatcagcaacaaagagtggctggagtatgctcagacatccgtaaagcattaa	The amino acid sequence was obtained from Uniprot accession no A0A182DWE3 and codon optimized for expression in *E.coli*. The DNA sequence was synthetized by Twist bioscience and cloned in place of the cas9 gene in pSpyCas9.
fnCas12	atgtcaatttatcaagaatttgttaataaatatagtttaagtaaaactctaagatttgagttaatcccacagggtaaaacacttgaaaacataaaagcaagaggtttgattttaga<br>tgatgagaaaagagctaaagactacaaaaaggctaaacaaataattgataaatatcatcagtttttatagaggagatattaagttcggtttgtattagcgaagatttattacaa<br>aactattctgatgtttattttaaacttaaaaagagtgatgatgataatctacaaaaagattttaaaagtgcaaaagatacgataaagaaacaaatatctgaatatataaaggac<br>tcagagaaatttaagaatttgtttaatcaaaaccttatcgatgctaaaaaagggcaagagtcagatttaattctatggctaaagcaatctaaggataatggtatagaactattta<br>aagccaatagtgatatcacagatatagatgaggcgttagaaataatcaaatcttttaaaggttggacaacttattttaagggttttcatgaaaatagaaaaaatgtttatagtag<br>caatgatattcctacatctattatttataggatagtagatgataatttgcctaaatttctagaaaataaagctaagtatgagagtttaaaagacaaagctccagaagctataaact<br>atgaacaaattaaaaaagatttggcagaagagctaacctttgatattgactacaaaacatctgaagttaatcaaagagtttttcacttgatgaagtttttgagatagcaaacttt<br>aataattatctaaatcaaagtggtattactaaatttaatactattattggtggtaaatttgtaaatggtgaaaatacaaagagaaaaggtataaatgaatatataaatctatactca<br>cagcaaataaatgataaaacactcaaaaaatataaaatgagtgttttatttaagcaaattttaagtgatacagaatctaaatcttttgtaattgataagttagaagatgatagtga<br>tgtagttacaacgatgcaaagtttttatgagcaaatagcagcttttaaaacagtagaagaaaaatctattaaagaaacactatctttattatttgatgatttaaaagctcaaaaac<br>ttgatttgagtaaaatttattttaaaaatgataaatctcttactgatctatcacaacaagtttttgatgattatagtgttattggtacagcggtactagaatatataactcaacaaatag<br>cacctaaaaatcttgataaccctagtaagaaagagcaagaattaatagccaaaaaaactgaaaaagcaaaatacttatctctagaaactataaagcttgccttagaagaa<br>tttaataagcatagagatatagataaacagtgtaggtttgaagaaatacttgcaaactttgcggctattccgatgatatttgatgaaatagctcaaaacaaagacaatttggca<br>cagatatctatcaaatatcaaaatcaaggtaaaaaagacctacttcaagctagtgcggaagatgatgttaaagctatcaaggatcttttagatcaaactaataatctcttacat<br>aaactaaaaatatttcatattagtcagtcagaagataaggcaaatattttagacaaggatgagcatttttatctagtatttgaggagtgctactttgagctagcgaatatagtgcct<br>ctttataacaaaattagaaactatataactcaaaagccatatagtgatgagaaatttaagctcaattttgagaactcgactttggctaatggttgggataaaaataaagagcct<br>gacaatacggcaattttatttatcaaagatgataaatattatctgggtgtgatgaataagaaaaataacaaaatatttgatgataaagctatcaaagaaaataaaggcgagg<br>gttataaaaaaattgtttataaacttttacctggcgcaaataaaatgttacctaaggttttcttttctgctaaatctataaaattttataatcctagtgaagatatacttagaataagaa<br>atcattccacacatacaaaaaatggtagtcctcaaaaaggatatgaaaaatttgagtttaatattgaagattgccgaaaatttatagatttttataaacagtctataagtaagcat<br>ccggagtggaaagattttggatttagattttctgatactcaaagatataattctatagatgaattttatagagaagttgaaaatcaaggctacaaactaacttttgaaaatatatca<br>gagagctatattgatagcgtagttaatcagggtaaattgtacctattccaaatctataataaagatttttcagcttatagcaaagggcgaccaaatctacatactttatattggaa	The sequence was amplified from Addgene plasmid #69973 (Zetsche *et al.*, 2015). The sequence of FnCas12 was cloned in place of the cas9 gene in pSpyCas9_Fig3C.

	agcgctgtttgatgagagaaatcttcaagatgtggtttataagctaaatggtgaggcagagctttttttatcgtaaacaatcaatacctaaaaaaatcactcacccagctaaaga<br>ggcaatagctaataaaaacaaagataatcctaaaaaagagagtgtttttgaatatgatttaatcaaagataaacgctttactgaagataagtttttctttcactgtcctattacaat<br>caattttaaatctagtggagctaataagtttaatgatgaaatcaatttattgctaaaagaaaaagcaaatgatgttcatatattaagtatagatagaggtgaaagacatttagctt<br>actatactttggtagatggtaaaggcaatatcatcaaacaagatactttcaacatcattggtaatgatagaatgaaaacaaactaccatgataagcttgctgcaatagagaa<br>agatagggattcagctaggaaagactggaaaaagataaataacatcaaagagatgaaagagggctatctatctcaggtagttcatgaaatagctaagctagttatagagt<br>ataatgctattgtggtttttgaggatttaaattttggatttaaaagagggcgtttcaaggtagagaagcaggtctatcaaaagttagaaaaaatgctaattgagaaactaaactat<br>ctagttttcaaagataatgagtttgataaaactgggggagtgcttagagcttatcagctaacagcaccttttgagacttttaaaaagatgggtaaacaaacaggtattatctact<br>atgtaccagctggttttacttcaaaaatttgtcctgtaactggttttgtaaatcagttatatcctaagtatgaaagtgtcagcaaatctcaagagttctttagtaagtttgacaagattt<br>gttataaccttgataagggctattttgagtttagttttgattataaaaactttggtgacaaggctgccaaaggcaagtggactatagctagctttgggagtagattgattaactttag<br>aaattcagataaaaatcataattgggatactcgagaagtttatccaactaaagagttggagaaattgctaaaagattattctatcgaatatgggcatggcgaatgtatcaaag<br>cagctatttgcggtgagagcgacaaaaagtttttttgctaagctaactagtgtcctaaatactatcttacaaatgcgtaactcaaaaacaggtactgagttagattatctaatttca<br>ccagtagcagatgtaaatggcaatttctttgattcgcgacaggcgccaaaaaatatgcctcaagatgctgatgccaatggtgcttatcatattgggctaaaaggtctgatgcta<br>ctaggtaggatcaaaaataatcaagagggcaaaaaactcaatttggttatcaaaaatgaagagtattttgagttcgtgcagaataggaataactaa	

References for S5 Table.

Esvelt, K.M., Mali, P., Braff, J.L., Moosburner, M., Yaung, S.J., and Church, G.M. (2013). Orthogonal Cas9 proteins for RNA-guided gene regulation and editing. Nat. Methods 10, 1116–1123.

Forsberg, K.J., Bhatt, I.V., Schmidtke, D.T., Javanmardi, K., Dillard, K.E., Stoddard, B.L., Finkelstein, I.J., Kaiser, B.K., and Malik, H.S. (2019). Functional metagenomics-guided discovery of potent Cas9 inhibitors in the human microbiome. Elife 8.

Rauch, B.J., Silvis, M.R., Hultquist, J.F., Waters, C.S., McGregor, M.J., Krogan, N.J., and Bondy-Denomy, J. (2017). Inhibition of CRISPR-Cas9 with Bacteriophage Proteins. Cell 168, 150-158 e110.

Zetsche, B., Gootenberg, J.S., Abudayyeh, O.O., Slaymaker, I.M., Makarova, K.S., Essletzbichler, P., Volz, S.E., Joung, J., van der Oost, J., Regev, A., et al. (2015). Cpf1 Is a Single RNA-Guided Endonuclease of a Class 2 CRISPR-Cas System. Cell.
