# Supplementary material for: The novel anti-CRISPR AcrIIA22 relieves DNA torsion in target plasmids and impairs SpyCas9 activity
Source: PLoS Biol. 2021 Oct 13;19(10):e3001428. doi: 10.1371/journal.pbio.3001428 (PMC8545432; doi:10.1371/journal.pbio.3001428)
Supplement: S1 Raw Images — (PDF) [file pbio.3001428.s025.pdf]

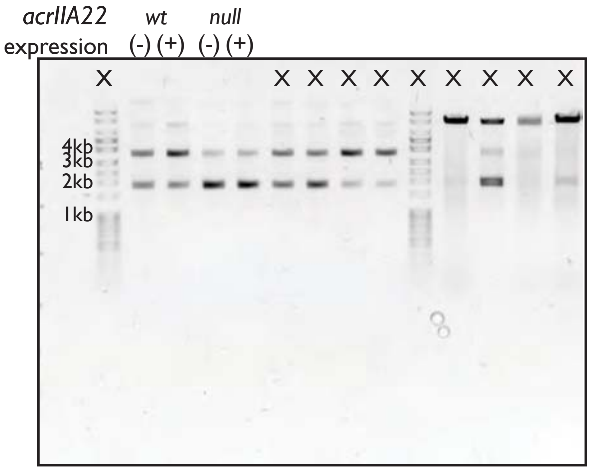

Figure 5A

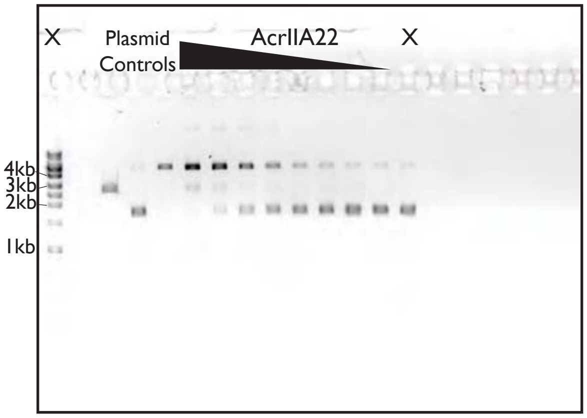

Figure 5B

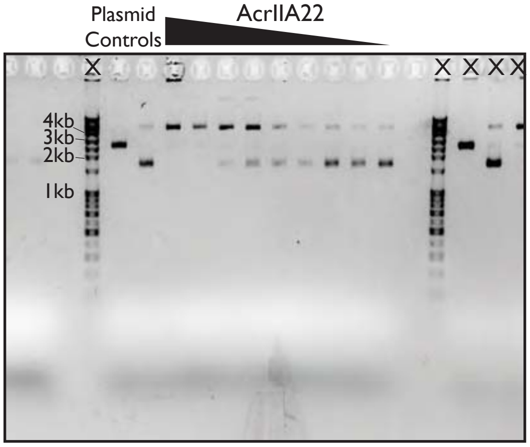

Figure 5C

X=lane not included in final figure (a marker, unused or redundant sample, or unrelated experiment)

These uncropped images appear cropped on occasion. This false crop effect results from the original camera zoom rather than any post-processing. The black borders were added to images to distinguish them from the white background. All images were captured in a Biorad geldoc and are EtBr DNA gels.

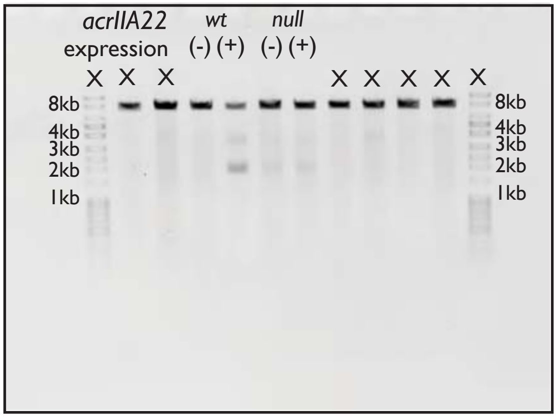

Figure 7A

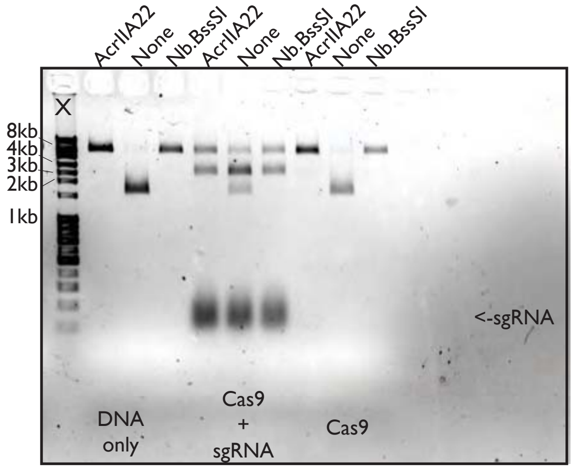

Figure 7C

X=lane not included in final figure (a marker, unused or redundant sample, or unrelated experiment)

The black borders were added to images to distinguish them from the white background.
All images were captured in a Biorad geldoc and are EtBr DNA gels.

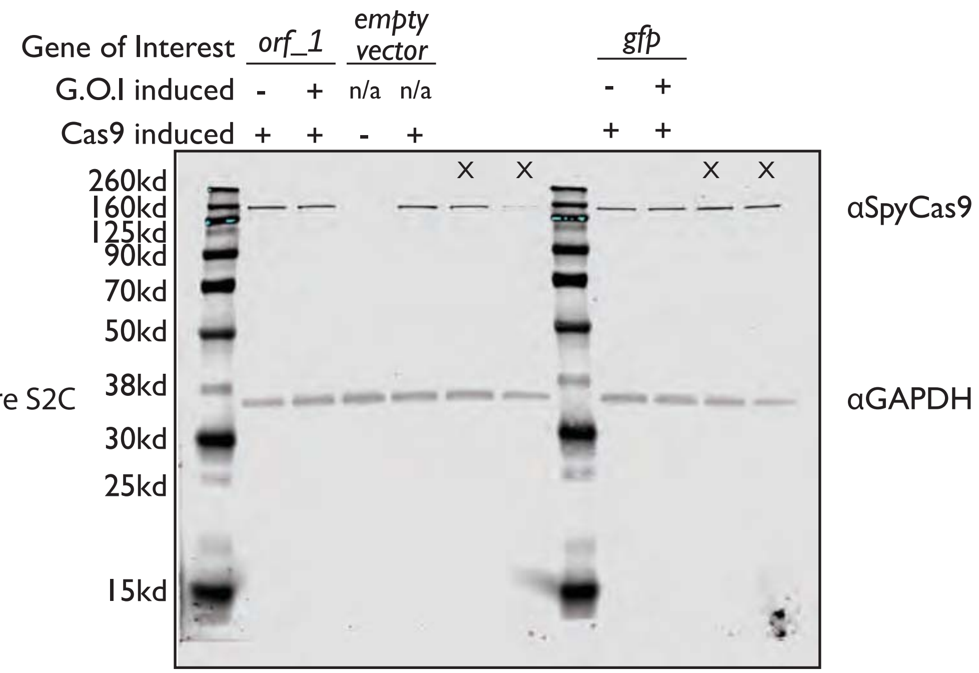

Figure S2C

3 hours SpyCas9 induction

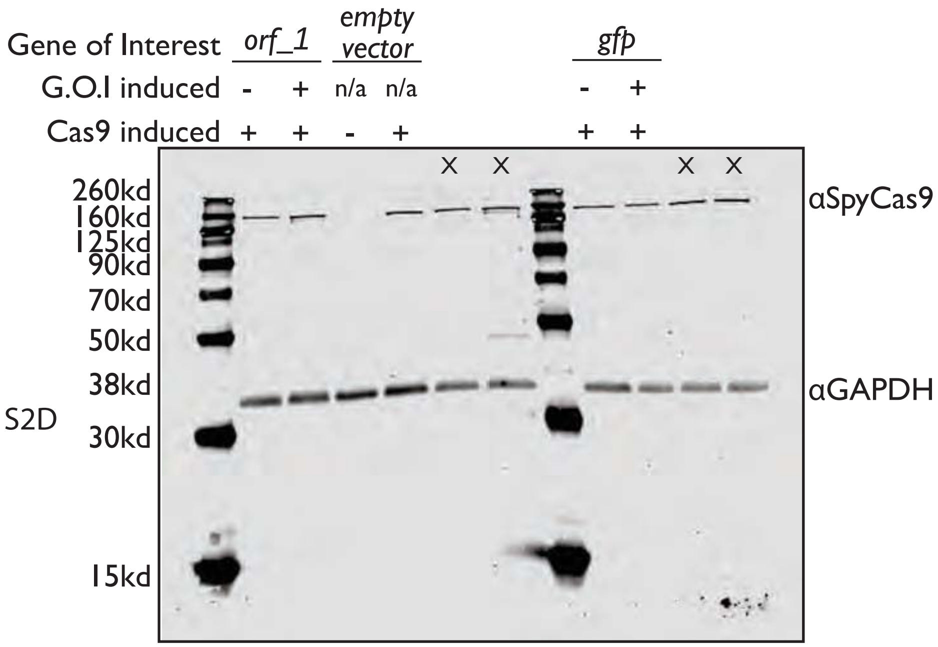

Figure S2D

6 hours SpyCas9 induction

X=lane not included in final figure (a marker, unused or redundant sample, or unrelated experiment)

The black borders were added to images to distinguish them from the white background.
All images were captured following a Western blot using a LiCor Odyssey Imager

Figure S6

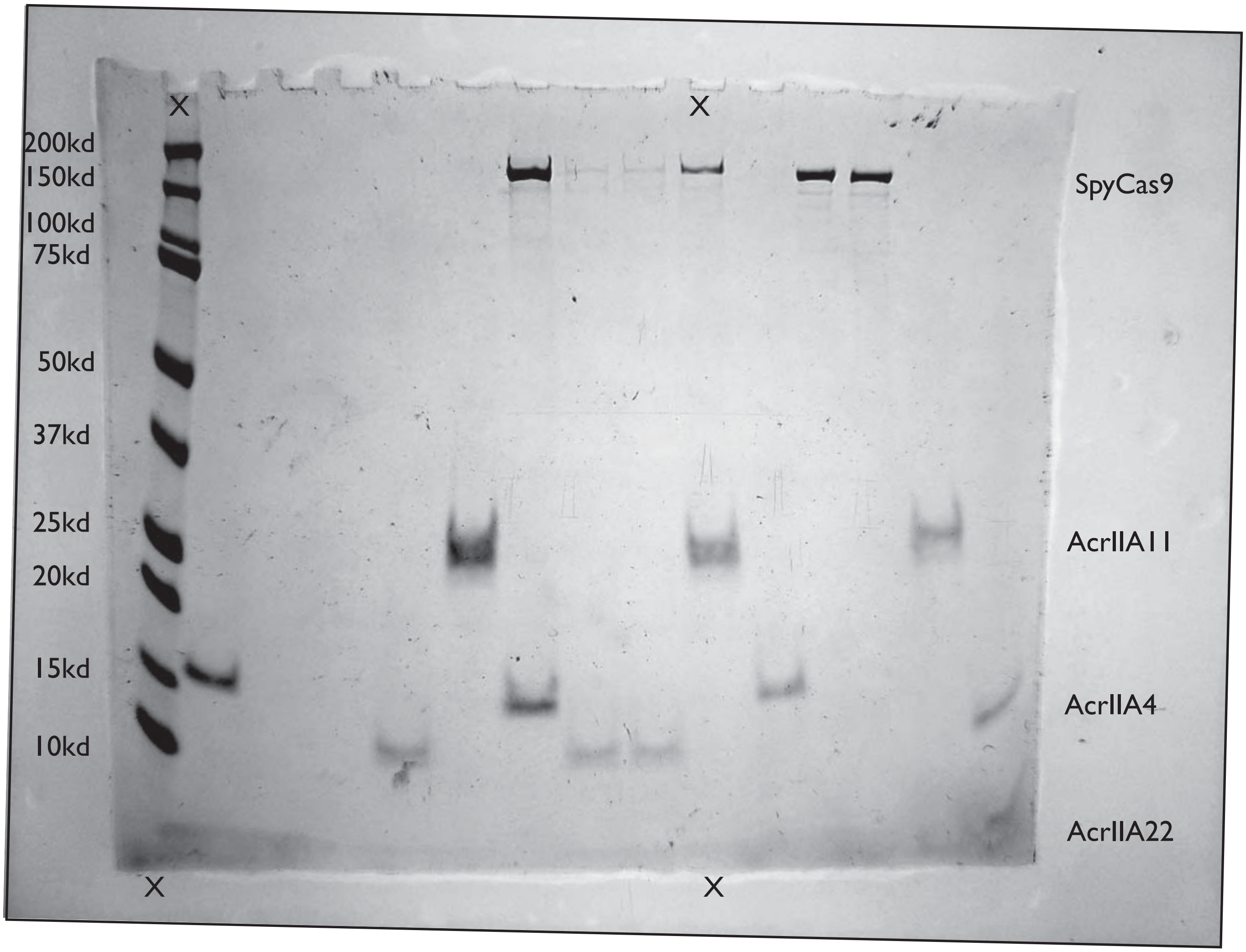

AcrIIA22-TS	-	-	-	+	-	-	+	+	-	-	-	-	+
AcrIIA4-TS	+	-	-	-	-	+	-	-	+	-	-	-	-
SpyCas9	-	+	+	-	-	+	+	+	-	+	+	-	-
sgRNA	-	+	-	-	-	+	+	-	-	+	-	-	-

AcrIIA11 bands omitted from figure S6 for clarity

X=lane not included in final figure (a marker, unused or redundant sample, or unrelated experiment)

The black border was added to images to distinguish it from the white background.
All images were captured via a cell phone camera following a Coomassie protein stain

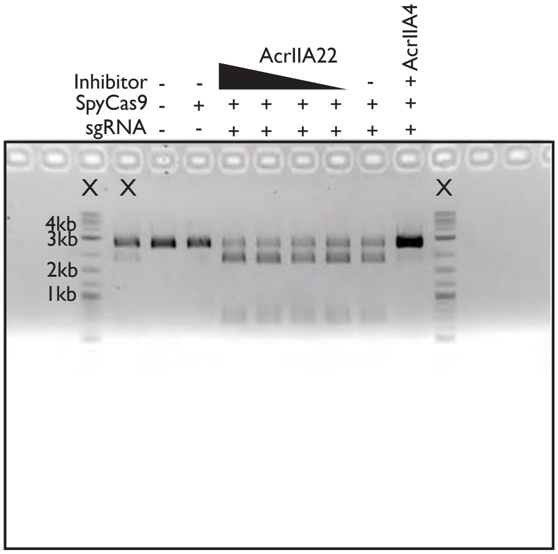

Figure S7B

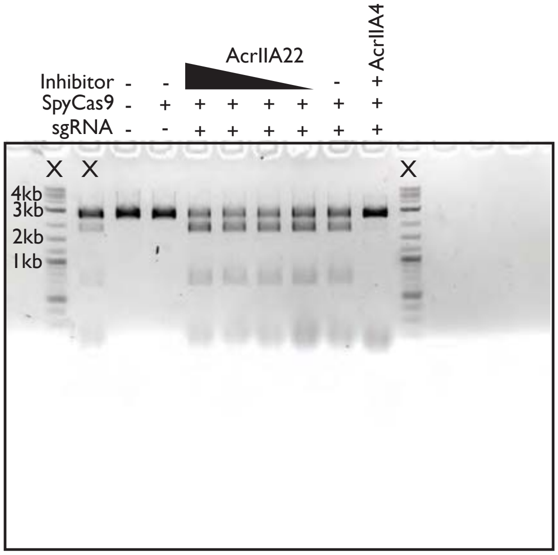

Figure S7D

X=lane not included in final figure (a marker, unused or redundant sample, or unrelated experiment)

These uncropped images appear cropped on occasion. This false crop effect results from the original camera zoom rather than any post-processing. The black borders were added to images to distinguish them from the white background. All images were captured in a Biorad geldoc and are EtBr DNA gels.

Figure S10A
(and part of S10B)

Total Protein (Coomassie Stain)

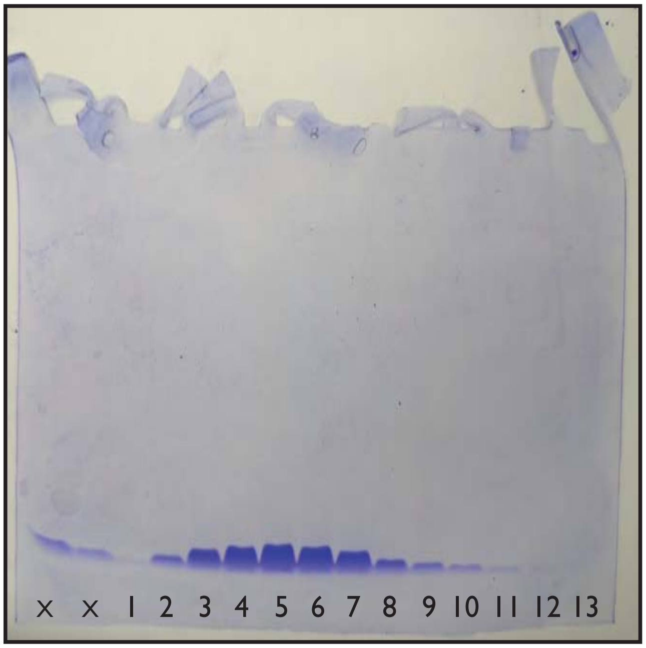

Figure S10B

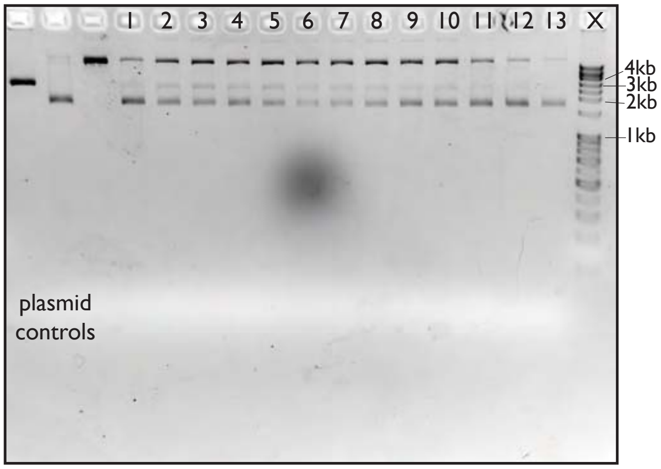

Figure S10E

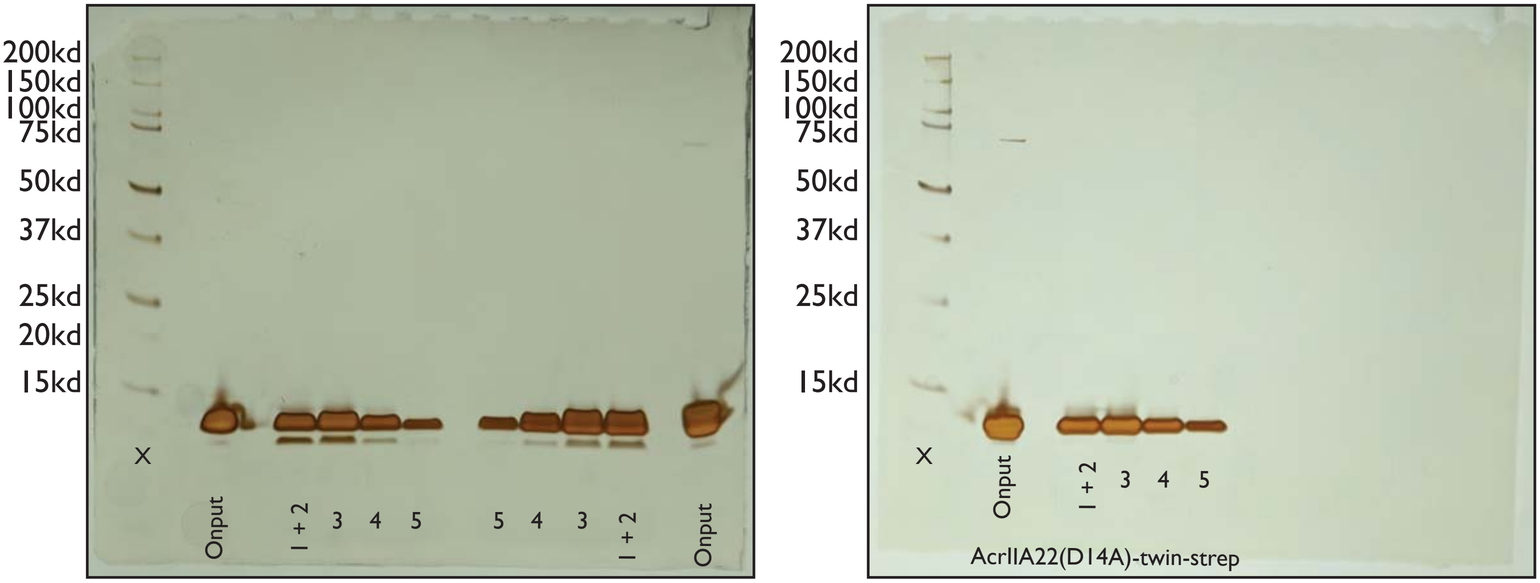

AcrIIA22-twin-strep

AcrIIA22a-twin-strep
(mirror flipped in S10E)

X=lane not included in final figure (a marker, unused or redundant sample, or unrelated experiment)

These uncropped images appear cropped on occasion. This false crop effect results from the original camera zoom rather than any post-processing. The black borders were added to images to distinguish them from the white background. Images were captured as follows:

(A) Cell phone camera + Coomassie Stain
(B) EtBr DNA Gel + BioRad Geldoc Imager
(E) Cell phone camera + Silver Stain

Figure S10E shows panesl from three samples across two gels, as shown.

Figure S10G

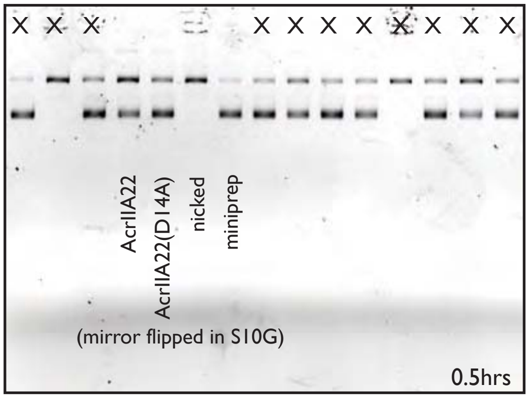

Figure S10H

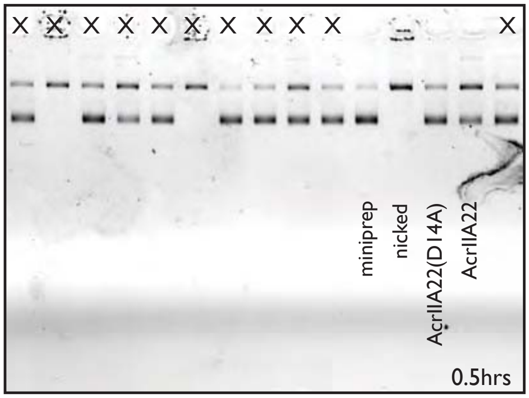

Figure S10G

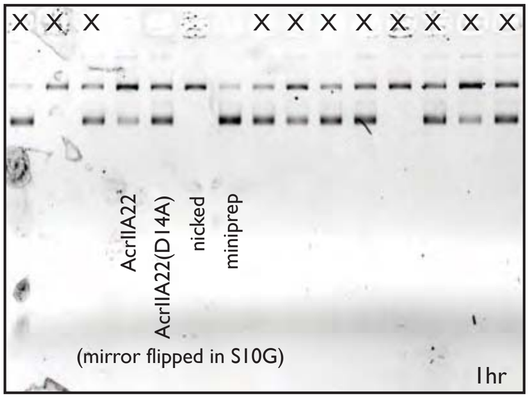

Figure S10H

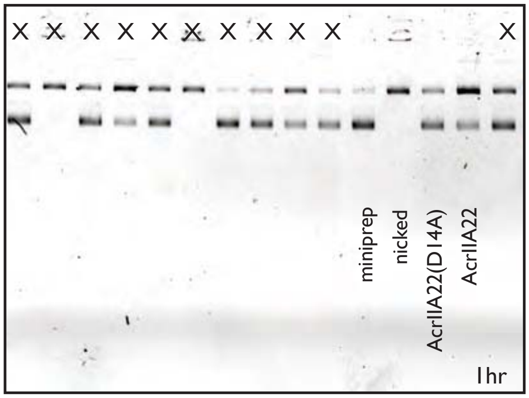

Figure S10G

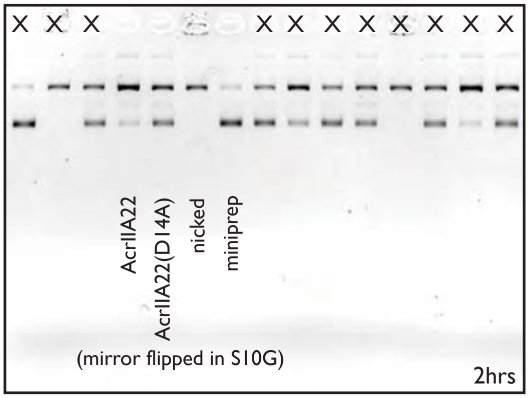

Figure S10H

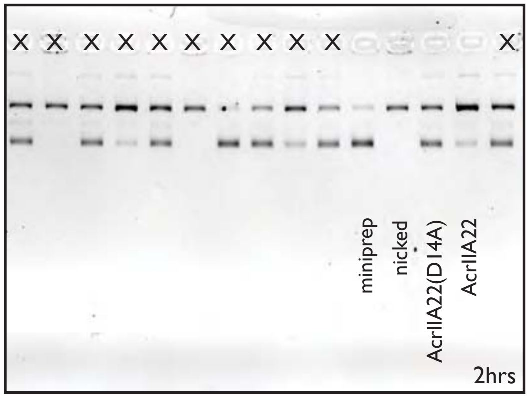

Figure S10G

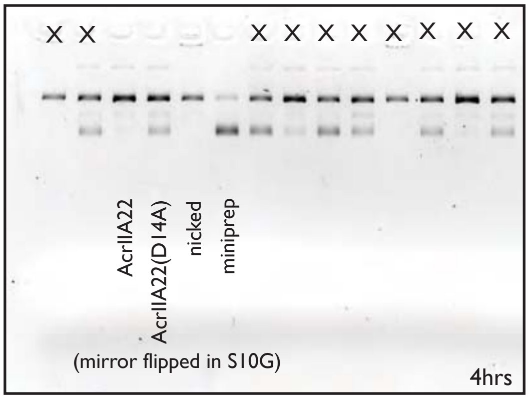

Figure S10H

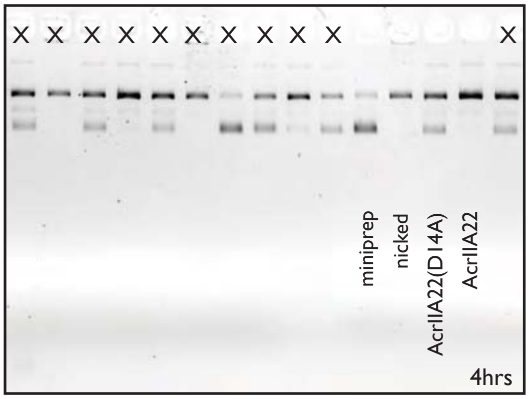

X=lane not included in final figure (a marker, unused or redundant sample, or unrelated experiment)

These uncropped images appear cropped on occasion. This false crop effect results from the original camera zoom rather than any post-processing. The black borders were added to images to distinguish them from the white background. All images were captured in a Biorad geldoc and are EtBr DNA gels.
