# Supplementary material for: The novel anti-CRISPR AcrIIA22 relieves DNA torsion in target plasmids and impairs SpyCas9 activity
Source: PLoS Biol. 2021 Oct 13;19(10):e3001428. doi: 10.1371/journal.pbio.3001428 (PMC8545432; doi:10.1371/journal.pbio.3001428)
Supplement: Attachment — Submitted filename: 112317_1_rebuttal_1964345_qmws1m.pdf [file pbio.3001428.s026.pdf]

We thank all reviewers and editorial staff for their careful consideration of our manuscript. We understand the time and effort that is required to provide constructive comments, and we are very appreciative.

In general, the reviews were largely positive, which was quite affirming. We also found each reviewer's suggestions for improvement to be very helpful and we are confident in our ability to address most concerns. In synthesizing these reviews, three topic areas seem to be the most critical: (i) a determination of the structural and functional basis for AcrIIA22's nickase activity, (ii) a clearer presentation of Cas9's lower activity on open circle plasmids, and (iii) a more detailed structural analysis of AcrIIA22 and its homology to PC4-like family proteins. We are optimistic that we can address each of these topics and agree with the reviewers that these additions will strengthen our manuscript. However, we do not anticipate that the associated experiments will substantially alter the major conclusions of our manuscript concerning AcrIIA22 or its anti-Cas9 activity, regardless of their outcomes.

Organizationally, we have formatted each ***reviewer's comments in bold*** *and indicate our responses in italics. For clarity, each significant reviewer comment is also enumerated across the entire document, so that we may easily refer to each specific point. Finally, we underline the specific actions we undertake to take as part of our Revision plan.*

Reviewer #1 (Evidence, reproducibility and clarity (Required)):

Forsberg et al. describe the characterization of a small 54-aa protein, encoded by hypervariable regions of clostridial bacteria and their prophages, that was selected from a metagenome library based on its capacity to completely protect a plasmid from being degraded by SpCas9. The authors call the protein an anti-CRISPR protein, AcrIIA22. However, characterization of the protein reveals a rather general mode of action. It does not, like many Acr proteins, directly interact with some of the key proteins of the CRISPR-Cas machinery. Its crystal structure reveals homology to PC4-like nucleic acid binding proteins such as the single-stranded binding protein of E.coli phage T5 that is involved in unwinding of dsDNA, and/or recombination. Analysis of the AcrIIA22 protein reveals it is has nickase activity that result in relaxation of supercoiled dsDNA. This activity appears to cause a substantial decrease in interference by SpCas9, protecting the plasmid from being cleaved. At the same time, it also caused slight toxicity as deduced from a 7% drop in growth rate. This is an interesting study, well executed and presented. There are some matters that should be addressed as indicated below.

We thank the reviewer for their positive remarks and helpful suggestions for improving our manuscript.

****Major comments****

1) As the authors admit, there is no direct interaction of AcrIIA22 with the type IIA Cas9 nuclease. Hence, the question is if this protein should be called an AcrIIA system, or even whether it should be classified as an anti-CRISPR protein. Given the indirect effect on Cas9 targeting, it is anticipated that the nickase activity will affect all enzymes that rely on a negatively supercoiled dsDNA configuration, possibly including non-CRISPR defence systems, but certainly including Type IE-IF Cascade/Cas3 systems (REF 32), Type IIA-C Cas9 systems (REFS 13-16). In addition, one would assume that also Type V Cas12 systems would be sensitive to the topology of their dsDNA target. So at best the name should be "general Acr protein".

We agree with the reviewer's prediction, that AcrIIA22 should be able to broadly inhibit Type II and perhaps other CRISPR-Cas systems, based on their preference for supercoiled DNA. In our Revision, we will test whether AcrIIA22 can inhibit other Cas9 and Cas12 Class II CRISPR-Cas systems in collaboration with two new co-authors: Ruben Uribe and Morten Sommer.

Regarding nomenclature, we agree and discuss the ambiguity about classifying AcrIIA22 among the currently described anti-CRISPRs because of its unconventional mode of action. Indeed, we devote substantial space in

our discussion section to this question (lines 381-391). We also provide significant genomic and phylogenetic evidence that independently supports our use of the 'Acr' designation and suggests that Cas9 may be AcrIIA22's specific target (lines 176-190). Consistent with these data, Cas9 is more dependent on negative supercoiling than most host enzymes and many CRISPR-Cas systems. Thus, we are confident calling AcrIIA22 an anti-CRISPR. We note that Reviewer 2 said that our "experiments convincingly establish that AcrIIA22 is a member of a family of anti-CRISPR proteins that inhibit Cas9 in both anti-plasmid and anti-phage interference", in agreement with our position.

Furthermore, we choose to maintain the 'IIA' sub-classification in AcrIIA22's name for three reasons: (i) type IIA CRISPR-Cas is thus far the only sub-type that we show is inhibited by AcrIIA22, (ii) as with other anti-CRISPRs, the 'IIA' classification matches the sub-type of CRISPR-Cas system for which AcrIIA22's activity was first established, and (iii) the genomic evidence (lines 176-190) supports Cas9 inhibition but no other CRISPR-Cas inhibition.

2) To make claims that the AcrIIA22 protein is more potent to protect MGEs against Type II Cas9 than against Type I Cascade, either this experiment should be done, or the claims (line 343-345) should be toned down.

<u>We will amend our language to make this point clearer</u>. We thank the reviewer for their suggestion.

3) A crystal structure of AcrIIA22 is provided, evidence is provided for metal-dependent nickase activity, and alignments have been made (to build a phylogenetic tree) of closely and distantly related sequences. Still, nothing is mentioned about conserved (metal-binding) residues that might constitute the catalytic site that allows for nicking. At least an in silico analysis should be included, and whenever possible an experimental verification of the prediction.

This point was raised by multiple reviewers; <u>we will do as this reviewer requests and provide an in-silico analysis. We also plan to make mutations to several residues across multiple putative active sites and test them functionally</u>. Though it is possible that the results of these experiments will be inconclusive (e.g. if mutations impact protein stability or oligomerization status), we will report them regardless of their outcome in our Revision.

4) Line 110 - Details should be provided on the genetic material of phage Mu (RNA/ssDNA/dsDNA, linear/circular), as basis for discussion why AcrIIA22 does not seem to protect it from Cas9 cleavage.

We agree with this comment. <u>We will add these details</u> in the discussion section.

****Minor comments****

Line 55 - provide detail on 'novel mechanism' of AcrIIA11

<u>We will add additional details</u> in our Revision.

Line 72 - what inducible promoter?

We apologize for the inadvertent omission. <u>We will add additional details</u> in our Revision.

Reviewer #1 (Significance (Required)):

This is an interesting study, well executed and presented. There are some matters that should be addressed as indicated above.

My expertise is Mol Microbiology, with focus on CRISPR-Cas biochemistry

We appreciate the reviewer's affirming praise and agree with his/her comments except for the AcrIIA22 nomenclature, <u>which we will justify better in our discussion</u>. As outlined, we intend to address each of the reviewer's suggestions in our Revision.

~~~~~~

Reviewer #2 (Evidence, reproducibility and clarity (Required)):

Anti-CRISPRs (Acrs) are small proteins harbored by phages that inactivate CRISPR-Cas systems and thereby enable tolerance of infection. Identifying Acrs and understanding their mechanism of action has proven to be important for both understanding host/phage interactions as well as controlling CRISPR-based biotechnologies, most notably, Cas9-mediated genome editing. The known Acrs are diverse in their amino acid sequence, protein structure, and mechanism of Cas inhibition. The authors recently described a genetic approach which identified a new Acr (AcrIIA11) by screening metagenomic libraries for inhibition of Cas9-mediated plasmid cleavage in E. coli (Forsberg et al. Elife 2019).

In this manuscript, the authors characterized a second metagenomic contig that was hit in the screen. They identified a single gene (AcrIIA22) from this contig which is responsible for inhibiting Cas9-mediated interference of the targeted plasmid and to a lesser extent, Mu phage. Using thorough bioinformatic analysis the authors detected several functional AcrIIA22 homologs in prophages and hypervariable islands within the Clostridial genus CAG-217. The authors purified the AcrIIA22 protein and determined its crystal structure, found that it forms a tetramer in solution, and identify residues required for oligomerization. Unlike most Acrs, this one did not exhibit detectable binding to its cognate Cas protein and did not inhibit Cas9 cleavage of linear DNA substrates. Instead, the authors used a structural homology search and plasmid mobility assays (both in vivo and in vitro) to show that AcrIIA22 possesses DNA nickase activity. They perform experiments on pre-nicked plasmid substrates that suggest AcrIIA22 inhibits Cas9 by removing the DNA supercoils that Cas9 requires for maximal activity.

We are grateful for the reviewer's careful reading of our manuscript, their summary, and their constructive suggestions.

Major comments:

5) The in vivo experiments convincingly establish that AcrIIA22 is a member of a family of anti-CRISPR proteins that inhibit Cas9 in both anti-plasmid and anti-phage interference. The in vitro data demonstrating that AcrIIA22 is a nickase are compelling as well. The Δ2aa mutant data suggest that oligomerization is required for both nicking and Cas9 inhibition. I commend the authors on their thorough exploration of AcrIIA22 function and evolution.

We thank the reviewer for their very kind comments.

6) I have some reservations regarding the claim that AcrIIA22-nicked plasmids are resistant to Cas9 cleavage. The major piece of experimental evidence supporting this idea is presented in Figure 6C, where plasmid substrates were pre-nicked by either AcrIIA22 or another nickase, and then are tested as substrates in SpyCas9 DNA cleavage assays. The manuscript text states "SpyCas9 showed a clear preference for cleaving the supercoiled substrate versus the AcrIIA22-treated open-circle plasmid", but after looking at the figure (either on screen or a print-out) I had some difficulty agreeing with this assessment. This is largely because in the reactions presented, Cas9-mediated cleavage of the un-treated substrate (Fig 6C - lane 5) has not gone to completion: most of the supercoiled substrate remains uncleaved and there is a fairly weak linear cleavage product. Both of the pre-nicked substrates (lanes 4 and 6) also appear to have weak linear product bands. To my eye, there is very slightly more linear Cas9 cleavage product for the supercoiled substrate than there is for pre-nicked. The authors note in the Figure 6 legend that some of the Cas9 cleavage product is open-circular (a single strand cut). However, the untreated plasmid (lane 2) also contains open-circular DNA. Altogether it is difficult to tell whether the nicked plasmid is indeed any less sensitive to Cas9 than the supercoiled form. A few suggestions / points related to this:
~~~~~~

We appreciate this suggestion and agree with the reviewer's central point: it would be ideal if figure 6C depicted complete Cas9 digestions. <u>We intend to achieve this goal, by incorporating the reviewer's helpful suggestions along the way;</u> they will strengthen the presentation and conclusions of figure 6C.

7) Can the reactions be run for longer time periods to generate more linear Cas9 product? This should make the difference between the nicked and supercoiled substrates more apparent, if the nicked substrates are indeed resistant to cleavage.

We agree with and appreciate this suggestion. <u>We will perform our assays with longer reaction times and incorporate some of the ideas offered by reviewer #3 (e.g. changing temperature).</u>

8) The experiment shown in Fig S10B (same experiment as Fig 6C, different reaction conditions) seems to suggest that pre-nicked substrates are still susceptible to cleavage by Cas9. The authors state in the legend that "the reaction proceeded too quickly to detect SpyCas9's substrate preference", however the reaction is only at about 50% completion and the three substrates have been cleaved equally. Why shouldn't the interpretation be that Cas9 prefers neither substrate?

The reviewer is right to point out an apparent discrepancy – figure S10B appears to show only 50% cleavage and yet this reaction has gone to completion; later timepoints with the same reaction also showed about 50% DNA cleavage. <u>We will include these later time points in a revised manuscript</u>. We can reconcile these seemingly paradoxical observations of fast, but partial, digestion because Cas9 is a single-turnover enzyme in vitro (it remains bound to the DNA substrate after cleavage). Thus, differences in Cas9 reaction rate can be difficult to detect in vitro compared to more canonical reactions with conventional, multi-turnover enzymes. With multi-turnover enzymes, small differences in reaction rate are amplified across successive reaction rounds. In our case, each functional Cas9 protein acts only once in vitro, so reactions proceed quickly. Cas9 acts as a multi-turnover enzyme in vivo when it is evicted from its DNA substrate by host proteins. Thus, changes to reaction rates can have multiplicative effects in vivo that are not detectable in vitro. The reviewer is right that we did not articulate this message well in figure S10; <u>we will improve our explanation in a revised manuscript</u>.

Importantly, Cas9's unique in vitro properties also impact other studies of anti-crispr activity. Many in vitro experiments do not depict complete digestion reactions because providing a stoichiometric excess of Cas protein required to drive the digestion reaction to completion could obscure the inhibitory effect of the anti-CRISPR under consideration. In our critical experiment, 44% of the DNA remains as supercoiled substrate (quantified in figure S10A), which is in line with previous assays using bona-fide anti-CRISPRs. Our goal in providing both Fig. 6C and Fig S10B was to be completely transparent about how different reaction conditions affect our ability to discern substrate preference. Empirically, we found that buffer conditions were important for slowing Cas9 activity to reveal its substrate preference. Reaction conditions can also determine efficiency of other anti-CRISPRs under different in vitro conditions. If the reviewer and editor feel this point provides a source of confusion, <u>we would be willing to eliminate Fig S10B in our Revision if recommended</u>.

Finally, we note that moderate reductions to Cas9 cleavage kinetics in vitro can cause major functional defects in vivo. This is because Cas9 is engaged in a kinetic race with its target, so must eliminate a target faster than the target can replicate (discussed on lines 312-315). For these reasons, we are confident that the moderate substrate preference that we demonstrate for Cas9 in vitro satisfactorily explains AcrIIA22's phenotype in vivo.

9) The authors state that AcrIIA22 "capitalizes on SpyCas9's stringent requirement for negative supercoils to form a productive R-loop". Does SpyCas9 strictly require negative supercoiling for R-loop formation and DNA cleavage? SpyCas9 is capable of cleaving short linear DNA substrates, which should have minimal supercoiling. Previous single-molecule studies investigating Cas9's supercoiling dependence (references 13-16) seem to suggest that rather than a stringent topology requirement, Cas9 binding and cleavage is enhanced by negative supercoiling. Thus, perhaps a quantitative assessment of cleavage kinetics for each substrate would be revealing?

We agree with the reviewer's comment. Although negative supercoiling is clearly important to Cas9 function, especially in vivo, it is not a strict requirement for activity in vitro, where it instead enhances function. <u>We will edit our language to reflect this more nuanced perspective</u>.

Reviewer #2 (Significance (Required)):

Anti-CRISPRs hold promise for enhanced control of Cas9-mediated genome editing. The discovery of a new Acr with a new mechanism of action could lead to tighter off-switches for Cas9, especially when used in combination with other known Acrs. Understanding the effects of DNA topology on Cas9 function will inform future technological uses of Cas9.

We thank the reviewer for their constructive suggestions and favorable comments.

****Referees cross-commenting****

10) I agree with Reviewer #1's major comments 1-2 regarding the expected general anti-nuclease property of AcrIIA22. Testing the inhibitory activity of AcrIIA22 against other CRISPR-Cas types would improve the study.

We also agree. <u>We will include these experiments in our Revision</u>, as outlined in response to comment #1.

~~~~~~

Reviewer #3 (Evidence, reproducibility and clarity (Required)):

Summary:

Forsberg et al. describe a new anti-CRISPR protein AcrIIA22, which was identified in a previously reported screen for anti-CRISPR effectors. A genomics analysis revealed that the presence of AcrIIA22 is a feature shared by diverse hypervariable regions of clostridial genomes. Crystal structure of AcrIIA22 is presented with the main finding being the structural basis of its oligomerization. This information was used to design a deletion mutant which did not oligomerize and was unable to inhibit Cas9. The authors also show that in vitro plasmids pre-treated with AcrIIA22 are nicked and less susceptible to Cas9 cleavage. These results lead to a model in which AcrIIA22 nicks DNA to relieve its supercoiling required for Cas9 activity. This inhibits Cas9 activity which would constitute a novel mode of indirect anti-CRISPR action.

We thank the reviewer for their accurate summary our manuscript.

Major comments:

11a) The main concern about this work is the proposed nickase activity of AcrIIA22. PC4 proteins are known to bind nucleic acids but have not been shown to have enzymatic activities. The basis of the proposed enzymatic activity of AcrIIA22 is in fact not even discussed in the paper. The metal dependence of the nickase activity (Suppl. Fig 9) follows a pattern typical for many metal-dependent nucleases with Mg2+ supporting the activity and Ca2+ being inhibitory. This would imply that AcrIIA22 is a typical nuclease with a metal ion-binding active site. No such active site has been identified in PC4-related proteins and it is not described in the current work based on the AcrIIA22 structure itself. To support the main conclusions of the manuscript, the authors need to better document AcrIIA22 nuclease activity for example by relatively simple experiments with short synthetic DNA substrates (single- and double-stranded).

Reviewer #1 raised similar concerns, that we have not sufficiently described the structural and functional basis for AcrIIA22's nickase activity (see comment #3). We agree with both reviewers that this is an important point to address. As discussed in response to reviewer #1, <u>we will identify candidate active sites in the proposed AcrIIA22 tetramer based on in silico analyses and test them via targeted mutagenesis and functional assays in vivo and in vitro. We will report these findings in our Revision</u>.
~~~~~~

11b) It is also very important to exclude the possibility that the nickase activity of purified AcrIIA22 comes from impurities in the protein preparation. DNase is a common activity in living cells. As a minimum, the quality of the protein needs to be shown in the Supplementary Information. The possible basis of the proposed enzymatic activity of PC4 needs to be thoroughly discussed, preferably based on the structural data.

We agree with this reviewer that it is important to address this possibility. To address it, we will provide two pieces of evidence that indicate AcrIIA22's nickase activity is not the result of co-purifying cellular nucleases. As the reviewer requests here, we will show the purity of the AcrIIA22 protein preparation in our supplementary materials. Furthermore, we will also show all raw gels that correspond to figure 5C, which demonstrates that an oligomerization deficient AcrIIA22 mutant is also impaired for nickase activity. Because this mutant was purified identically to the wild-type protein, this experiment suggests that impurities in the protein preparation do not underlie the observed nuclease activity.

12) A key element of the proposed mechanism is lower activity of Cas9 on open circle form of plasmid DNA. It is concerning that under certain conditions (Supp. Fig 10B), supercoiled and and nicked plasmids are cleaved by Cas9 equally well. The authors argue that this was due to the high reaction rate. This needs to be confirmed by time-course experiments at lower temperature which should slow down the reaction.

This point was also raised by reviewer #2. As we discuss in response to comment #6, we will improve the presentation of these results by incorporating the helpful suggestions from both reviewers.

13) Multiple structures of PC4 proteins in dimeric state and with nucleic acid bound are available (for example PMID: 16415882). The dimer architecture is conserved between PC4 and PC4-like protein from phage T5, so it is likely a universal feature of these proteins. Figure 4 needs to show more clearly how the typical PC4 dimerization is similar and different from the dimer present in the asymmetric unit of AcrIIA22 structure (Fig. 4A) and the proposed oligomeric state of the protein (Fig. 4D). Would the delta2aa mutant disrupt this canonical dimerization? Is the reported PC4 DNA binding mode compatible with the oligomeric state of AcrIIA22 or do authors imply a novel mode of the interactions with nucleic acid (dashed arrow in Fig 4E)? It is also important to show the ability of AcrIIA22 and its deletion mutant to bind single-stranded DNA. This can be done by a relatively simple extension of the gel filtration experiment shown in Fig 4C with protein-DNA complexes. Preferably, if possible, these experiments should be done with an in-line MALS-based measurement of the molecular weight. This will provide much more accurate MWs determination compared with column calibration traces.

We agree with this suggestion. We will provide the additional structural comparisons requested by the reviewer. These comparisons will show that the typical PC4 dimer resembles the lower portion of our proposed tetramer (depicted in blue hues in figure 4D). The predicted mode of DNA binding is broadly compatible with reports from other PC4-like proteins. The delta2aa mutant likely prevents the red-hued monomers from attaching to this blue-hued base, as we attempted to indicate via the dashed box in figure 4D and figure 4F. The asymmetric unit of AcrIIA22 depicts a dimerization interface along the N-terminal β-strand of each monomer (figure 4A). This is unlike most PC4 dimers but, interestingly, this interface may permit additional AcrIIA22 monomers to bind the exposed N-terminal β-strands of the red-hued monomers in figure 4D and form a higher-order oligomer. We find this interesting, in part, because the resulting filament-like structure would be reminiscent of the ssDNA-dependent oligomerization used by some PC4 homologs to unwind duplex DNA.

We did not discuss this possibility in our original manuscript because the functional impacts of this oligomerization (both for AcrIIA22 and other PC4 homologs) are unclear. However, it is an additional parallel between AcrIIA22 and other PC4-like proteins that we would be happy to discuss in our Revision.

While the detailed analysis of AcrIIA22's interaction with synthetic ssDNA fragments would certainly be interesting, especially to determine which biochemical properties it shares with other PC4-like homologs, we believe these experiments are outside the scope of our manuscript (which focuses primarily on AcrIIA22's anti-Cas9 function).

14) Samples of electron density maps are not shown in the manuscript and the detailed PDB validation report was not attached to this submission. However, based on Table 1 the structural work appears very solid with good dataset and structure statistics.

> *We thank the reviewer for their kind comments and apologize for the omission. <u>We will attach the detailed PDB validation report as part of our revised submission</u>.*

15) For in vitro experiments the numbers of replicates are not reported and quantification graphs do now show error bars. Quantification of nickase activity of delta2aa variant is shown in Fig 5C. This is an important experiment which may support the notion that the reported activity does indeed come from AcrIIA22 and not from impurities in its preparation. Therefore, the gels used to generate these data need to be shown in Supplementary Information, similar to the experiments for which the quantification is shown in Fig 5C.

> *We agree with this comment. As mentioned in our response to comment #11, <u>we will provide the supplementary gel images that correspond to figure 5C</u>, as requested by the reviewer. <u>We will also indicate the number of replicates performed and show error bars</u>.*

****Minor comments:****

16) Authors state the homology to PC4 suggested that AcrIIA22 is a nickase. However, the rationale for this is not clearly described. Otherwise, the presentation of the data is clear and previous work is properly referenced.

> *We thank the reviewer for pointing out that the rationale was unclear. The homology to PC4-like proteins prompted us to look at AcrIIA22's potential interaction with DNA but gave no initial indication of nickase activity. Instead, we empirically observed nickase activity when we began investigating AcrIIA22's effect on a target plasmid. We apologize if this logic was not clear. We <u>will edit our manuscript to more directly address this issue</u>.*

17) The nature of the proposed mechanism and the results with Mu phage (weak protection by AcrIIA22) suggest that the presence of AcrIIA22 would create an advantage for circular dsDNA phages and only when their genome is present in supercoiled form. The authors may want to discuss this and the prevalence of circular dsDNA phages of Clostridium bacteria.

> *This is a good suggestion; we thank the reviewer for it. <u>We will incorporate it into our revised Discussion</u>.*

Reviewer #3 (Significance (Required)):

18) The main finding from the structural part is the basis of AcrIIA22 oligomerization, but it needs to be further analyzed as described above. The fact that AcrIIA22 is similar to PC4-like proteins was likely apparent from the protein sequence because a homology model of the protein could be built which was successfully used for solving the crystal structure by molecular replacement.

> *<u>As indicated above, we intend to perform the structural analyses requested</u>. We also note that, though the initial AcrIIA22 homology model did show similarity to PC4-like structures, we were not confident in this homology until the final structure was solved. This is because AcrIIA22 folds into a relatively simple 4-stranded β-sheet. The homology model had only a moderate confidence score and was similar to many structures, due to its simplicity. These ambiguities were clarified only after our detailed structural analysis.*

19) Overall, this is a potentially very interesting work describing a novel mode of indirect CRISPR inhibition by relieving of DNA supercoiling. This could be an important conceptual advance. CRISPR mechanisms are obviously a very timely and popular subject, so this work would be interesting for a wide audience interested in these mechanisms.

We thank the reviewer for their very positive assessment of our manuscript's conceptual importance, broad appeal, and impact in the field.

Reviewer's expertise: nucleic acid enzymes, protein biochemistry, structural biology.
I cannot evaluate the genomics part of the manuscript very thoroughly .
