# Supplementary material for: The novel anti-CRISPR AcrIIA22 relieves DNA torsion in target plasmids and impairs SpyCas9 activity
Source: PLoS Biol. 2021 Oct 13;19(10):e3001428. doi: 10.1371/journal.pbio.3001428 (PMC8545432; doi:10.1371/journal.pbio.3001428)
Supplement: Attachment — Submitted filename: Forsberg_ReviewerResponse.pdf [file pbio.3001428.s029.pdf]

**<u>Comments from the Professional Editor:</u>**

**As you will see in their reports at the end of this email, the reviewers are generally satisfied with your responses to their comments and feel that the additional data supports the nickase activity of AcrIIA22. Nevertheless, you will also see from the outstanding concerns of Reviewer 1 and of the Academic Editor (whose comments you will also find at the end of this email) that they remain sceptical as to the native activity of AcrIIA22 and whether it functions as an anti-CRISPR protein in vivo via the proposed mechanism of relieving torsional stress in the DNA. Although we are editorially interested in the work and there is enough reviewer support to move forward, we ask that you carefully consider the concerns raised by the Academic Editor and by Reviewer #1 and respond to the points raised both in your 'Response to Reviewers' file and in the manuscript text by clearly discussing the caveats, recasting and tempering the claims if/as needed. It is ultimately in your best interest to put forth claims that have the best possible chance of standing the test of time.**

*Thank you to the editors and reviewers for there careful consideration of our manuscript. We are encouraged by all four reviews – their consensus is that our manuscript is greatly improved. We have entirely satisfied reviewers #2 and #3. The critique from reviewer #1 (Dr. John Van der Oost) states that we have under-claimed AcrIIA22's true breadth of activity: our data, in conjunction with its underlying mechanism, implies that AcrIIA22 may function as a more general anti-CRISPR than its name would otherwise suggest. Through our responses below, we believe that we also have addressed these concerns. Thus, we believe to have now addressed all substantive comments from <u>all</u> three original reviewers of our manuscript from Review Commons/PLOS Biology.*

*On the other hand, the academic editor points out that we have not definitively demonstrated a causal link between AcrIIA22's anti-CRISPR and nicking activities (though they do acknowledge that we have provided a "convincing correlation" between these properties). We agree with this comment. We have altered our title and relaxed our claims of causality in response. Though some lingering philosophical disagreement may prevent us from reaching a complete accord with the academic editor, we remain confident of the validity of our claims and are hopeful that our updated manuscript and point-by-point response will alleviate most of their remaining concerns.*

---

**Reviewer #1 (John Van der Oost, signs his review): I am not satisfied with the authors' response to some of my earlier comments, again copied below:**

**4) As the authors admit, there is no direct interaction of AcrIIA22 with the type IIA Cas9 nuclease. Hence, the question is if this protein should be called an AcrIIA system, or even whether it should be classified as an anti-CRISPR protein. Given the indirect effect on Cas9 targeting, it is anticipated that the nickase activity will affect all enzymes that rely on a negatively supercoiled dsDNA configuration, possibly including non-CRISPR defence systems, but certainly including Type IE-IF Cascade/Cas3 systems (REF 32), Type IIA-C Cas9 systems (REFS 13-16). In addition, one would assume that also Type V Cas12 systems would be sensitive to the topology of their dsDNA target. So at best the name should be "general Acr protein".**

*INITIAL AUTHOR RESPONSE: We have followed the reviewer's suggestion and have tested AcrIIA22 against additional CRISPR-Cas systems (revised Figure 3C). We chose type II and type V systems, as these are the only ones naturally occurring with its host CAG-217 genus. We found that AcrIIA22 can protect plasmids from Cas9, but not Cas12-based systems. Thus, we have elected to keep the type II nomenclature for AcrIIA22.*

**REVIEWER RESPONSE: The data provided on Cas12 are convincing, their activity seems not to be affected by the nickase. Still, the currently used name (AcrIIA22) suggests that the protein specifically counteracts Type IIA systems. However, it also counteracts the NmCas9, which is Type IIC. Hence, it seems to be a rather generic nickase that affects the functionality of enzymes that rely on negative supercoiling (including Type I, see below).**

*Author Re-Response: We thank reviewer #1 for their praise and for raising this important question of nomenclature. We agree that AcrIIA22's activity against II-A and II-C systems could warrant a more encompassing gene name, especially when considered in conjunction with the general nature of its*

*proposed mechanism. However, nearly all previous examples of broadly acting anti-CRISPRs have been named solely for the CRISPR system against which activity was originally identified, despite additional antagonisms being later discovered. Some examples include AcrIIA5, which works against II-A and II-C subtypes (Garcia, 2019), AcrIC6/C7/C8 against I-C and I-E subtypes (León, 2021), and AcrVA3 against V-A and I-C systems (Marino, 2018). The only exceptions to this convention are the AcrIE4-IF7 fusion protein and AcrIII-1, which cleaves cyclic oligonucleotides to impair any type III system that uses these second messengers. Since AcrIIA22 does not fall neatly into either of these exceptions, we choose to maintain the conservative 'AcrIIA' prefix among our imperfect options. Reassuringly, this subtype is by far the most prevalent in AcrIIA22's host genus and probably represents a real-world selection pressure for its phages (unlike type II-C systems).*

**5) To make claims that the AcrIIA22 protein is more potent to protect MGEs against Type II Cas9 than against Type I Cascade, either this experiment should be done, or the claims (line 343-345) should be toned down.**

*INITIAL AUTHOR RESPONSE: We thank the reviewer for their suggestion. We have amended our language to make this point clearer. We now refer to published in vitro data that demonstrates Cas9 is more susceptible to supercoil state than type I Cascade.*

**REVIEWER RESPONSE: It is very difficult to compare experiments that have been independently reported in the literature. As said before, either experiments have to be included in which a direct comparison of Cas9 and Cascade is performed, or the clains that this nickase only affects Cas9 should be rephrased in: "this nickase affects the functionality of proteins/enzymes that rely on negative supercoiling, including Cas9, but probably also other (Cas/non-Cas proteins/enzymes".**

*We agree with reviewer #1 and have tempered our claims accordingly. We still cite the original references but rephrase our interpretation as suggested.*

---

**Reviewer #2: The authors have gone to great lengths experimentally to address each of the points raised in review. The new data has convinced me that AcrIIA22-nicked plasmids are less sensitive to Cas9 cleavage in vitro. The additional mutants and biochemical analyses are compelling evidence of AcrIIA22-mediated nicking, and in every case, there is a strong correlation between nickase activity and Cas9 inhibition. The demonstration that AcrIIA22 is functional in preventing targeting by NmeCas9 and SpyCas9, but not either Cas12 homolog, argues that AcrIIA22 is not simply coating DNA and non-specifically interfering with nuclease activity. Overall, the authors have provided a thorough mechanistic characterization of AcrIIA22 and provided solid evidence to support their claims; therefore I recommend publication.**

*We thank reviewer #2 for their kind praise.*

---

**Reviewer #3: The authors addressed my comments appropriately. In particular they provided additional experiments supporting the nickase activity and prepared panels with structural analysis.**

*We thank reviewer #3 for their kind praise.*

---

**Academic Editor Comments:**

**This revised manuscript is improved over the original. The authors have provided much stronger evidence that AcrIIA22 is a nickase. They also show a convincing correlation between the nickase activity of AcrIIA22 and its plasmid-rescuing activity in vivo.**

*We are pleased that the academic editor agrees (1) AcrIIA22 is a nickase, (2) that it inhibits SpyCas9, and (3) that there is a "convincing correlation" between these activities. The academic editor has two major criticisms of our*

*findings: (1) that we fail to demonstrate a causal link between these behaviors and (2) that AcrIIA22 may not function as an anti-CRISPR in its natural context. In this point-by-point response, we address each of these concerns, acknowledge the causality criticism (for instance, by changing the title), and re-state why we remain confident in our conclusions (which have satisfied all three original reviewers).*

**0. Despite the improvements in the manuscript, I am still entirely unconvinced by the proposed mechanism for this Acr. In addition, there is also no reason to believe that this protein functions as an anti-CRISPR within the context of the phages where its gene is located.**

*To provide context here, there seems to be little disagreement from any editor or reviewer on the following points:*

- *We identified an entirely novel antagonist, AcrIIA22, which inhibits SpyCas9 in a heterologous system.*
- *We have characterized its biochemistry and structure to demonstrate that AcrIIA22 is a nickase*
- *We show that SpyCas9 cleaves nicked and AcrIIA22-treated plasmids poorly*
- *For multiple mutants, AcrIIA22's nicking activity in vitro correlates with its anti-CRISPR activity in vivo.*

*Taken together, we believe these points provide good evidence that AcrIIA22 uses a torsion-based mechanism to inhibit SpyCas9. Due to their obscurity, almost no data are available on the native biology of AcrIIA22, its host bacteria, or its host phages. With only genome sequence to guide us, we cannot be sure of AcrIIA22's true role in nature. However, the available evidence supports a natural anti-CRISPR function for AcrIIA22, in line with the findings from the rest of our manuscript. In particular, the sequence data indicate that:*

- *Type II-A CRISPR-Cas systems predominate among AcrIIA22-encoding bacteria*
- *AcrIIA22 shares genomes, gene neighborhoods, and other genomic features with bona-fide Acrs.*
- *These observations are unusual for a typical clostridial gene but are expected for a type II-A anti-CRISPR.*

*Thus, we strongly believe there is reason to call AcrIIA22 an anti-CRISPR. Nonetheless, we acknowledge this remains a hypothesis of AcrIIA22's true role in nature and devote much of paragraph #4 in our discussion section to this topic.*

**These are the problems:**

**1. AcrIIA22 displays absolutely no in vitro inhibition of SpyCas9 activity on linear dsDNA.**

*We were surprised by this criticism; this observation is completely consistent with our model. <u>A torsion-based defense mechanism against SpyCas9 should not protect linear DNA</u>, which is relaxed. We make this point in the first paragraph of our discussion section.*

**2. AcrIIA22 only very weakly prevents SpyCas9 from blocking phage infection.**

*To provide context, <u>AcrIIA22 confers 100-1,000-fold protection to phage Mu against SpyCas9</u>. This is not an insubstantial degree of protection. Admittedly, this activity is less than that provided by other, well-characterized anti-CRISPRs which is why we have originally described this as 'weak' in relative terms. This difference in activity is probably because traditional anti-CRISPRs bind SpyCas9 so are less sensitive to differences in phage replication strategies or DNA topology than AcrIIA22, which has an unusual, DNA-centric mechanism. Considering how little is known about AcrIA22's natural phages, it is remarkable that it protects phage Mu at all, especially in light of Mu's unusual transposition-based replication strategy, in which it spends little time as an extrachromosomal element where it is most susceptible to torsional effects. Like our response to point #1, we discuss this topic in the first paragraph of our discussion.*

**3. The in vitro assay for AcrIIA22 activity on super-coiled DNA does not seem physiologically relevant. As I understand it, the authors first treat DNA with AcrIIA22, then purify the open circle DNA produced. This DNA is then treated with SpyCas9. In vivo, whether a plasmid is introduced into a cell by conjugation, or a phage genome is introduced by injection, the CRISPR-Cas system will be present before any Acr is produced. Thus, an Acr must have strong inhibitory activity in the presence of the CRISPR-Cas system. Pre-treating DNA with**

**AcrIIA22 and then treating it with SpyCas9 is entirely artificial with respect to the in vivo situation. What would happen if SpyCas9 and AcrIIA22 were added to a reaction at the same time?**

*It is not informative to add SpyCas9 and AcrIIA22 simultaneously in this in vitro assay because both enzymes produce linear DNA via an open-circle intermediate. This makes it difficult to attribute any specific band on a gel to the activity of either enzyme.*

*Importantly, our assay was not designed to mimic physiological conditions, especially because so little is known about AcrIIA22's native context. Instead, it tested whether open-circle DNA was resistant to SpyCas9 cleavage, a key pillar of our model. Nonetheless, there are scenarios in which this assay may be physiologically relevant. As we outline in the penultimate paragraph of our discussion, weak Acrs provide numerous benefits to a host phage. In fact, in the context of early phage infection, it may be easier for a phage to quickly reap these benefits by modifying the topology of its single-copy genome than to inactivate many pre-circulating Cas9 proteins.*

**4. There is no assay where the authors treat linear DNA, open circle DNA, and supercoiled DNA with SpyCas9 under the same conditions and run on the same gel. What is SpyCas9 most active in cleaving? From what is shown, it looks like SpyCas9 cleaves linear DNA faster than supercoiled. I do not understand how a DNA cleaving enzyme would cleave supercoiled and linear DNA well, but cleave open circles poorly. Topologically, I don't see how linear DNA and open circle DNA would be distinguishable in vitro. They are both fully relaxed. Or am I missing something? The authors must provide an explanation for this. The authors say, "Our findings are consistent with previous in vitro experiments, which indicated that Cas9 requires a higher degree of negative supercoiling than type I CRISPR-Cas systems to provide the free energy needed for R-loop formation." If this were true, wouldn't Cas9 cut supercoiled DNA much better than linear? Why is it only open circle that seems to be cut poorly?**

*We recognize that the confusion regarding SpyCas9's preference for linear vs supercoiled DNA stems from a comparison of figures 7 and S7. Figure S7 depicts a test of whether AcrIIA22 prevents SpyCas9 from cleaving linear DNA and was performed as part of a standard, early inquiry of AcrIIA22's anti-CRISPR properties. This occurred years prior to our interrogation of supercoiled and open-circle DNA substrates (figure 7) and used different reaction conditions with different (more active) Cas9 enzyme preparations. Therefore, a head-to-head comparison of these figures is inappropriate. For this reason, we avoid discussing these results in connection with one another in our original manuscript. Importantly, both experiments were internally well-controlled and our claims of SpyCas9 substrate preference are robustly supported by independent experiments performed in triplicate. However, we agree this could be a source of confusion; we now explicitly caution against comparing figures 7 and S7, in the caption of figure S7.*

*Linear and supercoiled substrates have been directly compared multiple times in the literature on Cas9 enzymology, which consistently indicates the importance of negative supercoils, and which we cite to support our model (e.g. Ivanov, 2020). For this reason, and because linear DNA is less relevant to our in vivo plasmid protection assay (because it is rapidly eliminated in cells), we do not believe an additional experiment with linear, open-circle, and supercoiled substrates would meaningfully add to our manuscript.*

**5. The authors state that "we show that AcrIIA22 acts by nicking supercoiled DNA to relieve torsional stress on a target plasmid, thereby impairing SpyCas9 activity in vivo and in vitro." This is misleading. The authors have not shown that the relieving of torsional stress is inhibiting SpyCas9 activity in vivo. They have shown a correlation between nicking activity in vitro and plasmid rescue in vivo. They have no evidence that SpyCas9 is responding to the degree of torsional stress in vivo. In fact, the torsional state of the plasmids in vivo has not been assayed at all. The only "in vivo" assay of torsional stress comes from plasmid isolation after growth in the presence of AcrIIA22. It is possible that the plasmid nicking observed on gels occurs during the preparation of the DNA (i.e. AcrIIA22 nicks the DNA in vitro after cell lysis). Maybe the nickase activity of AcrIIA22 stimulates plasmid recombination which helps overcome SpyCas9 cleavage.**

*We appreciate this suggestion and agree with the academic editor. We have shown AcrIIA22 behaves as an anti-CRISPR, that it is a nickase, and that these activities correlate. However, we acknowledge that we have not*

*definitively demonstrated a causal link between AcrIIA22's anti-CRISPR and nickase functions and have updated this paragraph accordingly. We have now rephrased the title and key parts of the paper to make this clear.*

**6. To explain how AcrIIA22 works, the authors write, "We speculate that AcrIIA22 treads a fine balance between activity and toxicity; its nicking activity is high enough to antagonize SpyCas9 in a kinetic race, but not so high that it would be toxic to the host cell (Supplemental Figure 1). To strike this balance, AcrIIA22's nicking activity may somehow be regulated in vivo, perhaps via transient interactions with Cas9, other host factors, or its oligomerization". If AcrIIA22 must tread such a fine balance in vivo that might involve host factors, why is it working in a totally artificial system in which it is overexpressed and blocking a type of Cas9 that it would not encounter in nature? I still find it hard to imagine how a presumably non-specific nickase would be able to provide just the right amount of nicking in vivo to inhibit Cas9 but not inhibit other processes that are crucial for the phage or plasmid. To me, this mechanism appears to be fundamentally infeasible.**

*We feel the academic editor is not considering the important role of a number of overlapping host processes that could buffer the effects of AcrIIA22's nicking activity on its plasmid protection and toxicity phenotypes. These include widespread mechanisms for DNA repair or for homeostatic control of DNA topology and plasmid copy number. Such processes are expected to be operational in many heterologous systems, which means a broader range of nicking activities could yield the observed outcome than one may intuit. Moreover, AcrIIA22 was selected from a library of millions of clones for its plasmid protection phenotype and so any perceived improbability must be counterbalanced against the huge number of enzymes screened in this library.*

*Importantly, more specific forms of regulating AcrIIA22 activity may exist in its native context, which are not mutually exclusive with these general homeostatic mechanisms. Options include regulation via oligomerization, heterotypic interactions with Cas9 or other factors, or preferred DNA sequence/structural motifs. Though we cannot reasonably guess which forms of regulation operate in AcrIIA22's native context, we consider the general phenomenon to be likely for exactly the reasons mentioned by the academic editor – that it must avoid impairing other host processes. In response to this critique, we have modified the paragraph in question to specifically mention these possibilities.*

**To conclude, I do not doubt that AcrIIA22 inhibits SpyCas9-mediated plasmid loss in E. coli and I accept that AcrIIA22 has nickase activity in vitro. I am not convinced that relief of torsional stress is the mechanism for the in vivo activity of AcrIIA22 or that the biological role of AcrIIA22 is to act as an anti-CRISPR. Given that the title of this paper is "AcrIIA22 is a novel anti-CRISPR that impairs SpyCas9 activity by relieving DNA torsion of target plasmids", my objections create a problem for publication of the manuscript in its current state.**

*We agree that the causal link between AcrIIA22's nicking and anti-CRISPR activities has not been definitively established. Instead, as the academic editor has mentioned, we have only demonstrated a "convincing correlation" between these properties. In accord with this view, we have now altered our title and have rephrased the appropriate sections of our manuscript.*
