# Supplementary material for: The novel anti-CRISPR AcrIIA22 relieves DNA torsion in target plasmids and impairs SpyCas9 activity
Source: PLoS Biol. 2021 Oct 13;19(10):e3001428. doi: 10.1371/journal.pbio.3001428 (PMC8545432; doi:10.1371/journal.pbio.3001428)
Supplement: Attachment — Submitted filename: Forsberg_Reviewer_Response.docx [file pbio.3001428.s028.docx]

*We thank all reviewers and editorial staff for their careful consideration of our manuscript. We appreciate the time and effort that are required to provide constructive comments. In general, the reviews were positive. Where editors and reviewers made suggestions for improvement, we found these to be very helpful and are grateful for the constructive criticisms. We have performed new computational and experimental analyses and have repeated key experiments that we believe have satisfactorily addressed the issues that they raise. Major new experiments and analyses in our revision are summarized in the bulleted list below and detailed in the point-by-point response that follows. For ease of reference, we have re-numbered all editorial and reviewer comments sequentially.*

- ***We perform extensive new biochemical analyses that establish AcrIIA22 is a nickase.*** *We isolated pure AcrIIA22 via two different purification schemes, provide Coomassie and silver stains to demonstrate purity, quantify nicking activity, and demonstrate that activity tracks with protein content across fractions.*
- ***We describe natural and engineered AcrIIA22 mutants deficient for nicking activity.*** *An alanine scan of putative catalytic residues revealed a single amino acid change that substantially impaired nickase function. Additionally, a natural AcrIIA22 homolog that differs by two amino acids from our original hit shows reduced nicking activity. In both instances, these nickase-deficient mutants are impaired for SpyCas9 inhibition in vivo, linking these phenotypes. Neither mutant alters the oligomerization of AcrIIA22.*
- ***We have added substantial new structural comparisons.*** *These analyses support AcrIIA22’s homology to PC4-like family proteins, clarify our model for its oligomerization, and provide structural rationale for nickase-deficient mutants we identify.*
- ***We strengthen evidence for SpyCas9’s preference for supercoiled DNA substrates.*** *We optimized our Cas9 cleavage assay to depict substrate preference more clearly. We also provide triplicate measurements, time course data, and statistics to support our claims.*
- ***We test AcrIIA22’s inhibitory range across multiple CRISPR-Cas types.*** These analyses show that AcrIIA22 can inhibit multiple diverged Cas9 orthologs, but no Cas12-based CRISPR-Cas system. This result is consistent with the distribution of CRISPR-Cas types in AcrIIA22’s host genus, CAG-217: most systems encode Cas9, a rare few encode Cas12, and no other CRISPR-Cas type is detected.

Some of these additional experiments were carried out by new collaborators, Ruben Uribe and Morten Sommer, who are now added as co-authors in the revision. To comply with submission guidelines, areas of the main text which discuss substantially new or changed material have been indicated by highlight.

**Academic editor:**

**1a) The authors have not proved that AcrIIA22 is a nickase. The proteins that are close in structure are not nickases. There is no obvious active site in AcrIIA22. The authors do no not discuss sequence similarity between their anti-CRISPR and any enzyme that cuts DNA. I assume that there is none. The biochemistry is not convincing.**

*We thank the editor for this critique. It strikes at the core of our work and the opportunity to address it have significantly improved our manuscript. We have now added substantial structural and biochemical data to support our claim of AcrIIA22’s nicking activity. These data are summarized in responses below and detailed in revised Figures 4, 5, 6 and revised Supplemental Figures 8, 9, 10. We also discuss AcrIIA22’s lack of sequence similarity to known nucleases (or any known protein).*

**1b) To support that AcrIIA22 is a nickase, careful fractionation of preparations through at least two columns would need to be documented. The peak of nickase activity needs to correlate with the peak of AcrIIA22 protein elution. Nickase activity needs to be assayed in all fractions especially those with little or no AcrIIA22 in them. Nickase could easily come from a contaminating protein present at low abundance. Assuming that AcrIIA22 is a nickase, what is the level of activity (e.g. rate of nick introduction/weight of protein/time). How does this rate compare to known nickases.**

*We thank the editor for these detailed suggestions to provide more convincing evidence of ArcIIA22’s nicking activity. We have performed these suggested experiments and have added additional analyses in our revision. (1) We re-purified AcrIIA22 as requested and performed the suggested fractionation experiment, which shows a strong correlation between nicking activity and peak protein elution. (2) To address the issue of a co-purifying contaminant, we also purified AcrIIA22 via a different affinity tag (using three steps of column chromatography) and demonstrate that this new purification scheme preserves nicking activity. We now show that neither Coomassie nor silver stains reveal contaminating proteins, supporting our claims of AcrIIA22’s purity. (3) The second (new) purification scheme used a C-terminal twin-strep tag and exhibited much higher nicking activity than our initial, N-terminal His6-tagged preparations. This result was mirrored in vivo; a C-terminally tagged AcrIIA22 construct could inhibit SpyCas9 whereas an N-terminally tagged construct could not. Thus, tag placement may explain the low nicking activity observed in our initial protein preparations that we reported in our original submission. These data can be seen in Figure 5 and Supplemental Figure 10. (4) We now quantify the rate at which supercoiled plasmids are nicked and cite another nickase involved in phage-bacterial conflicts with similar kinetics. Overall, these additional experiments provide much stronger support for our nickase claims.*

**1c) Purification of the deletion mutant of AcrIIA22 (2 aa truncation) is not a valid control. This protein has a different oligomerization state. Proteins co-purifying with it may be completely different than wild-type. The protein could also be partially unfolded or much less stable than wild-type, which would also alter its chromatographic behaviour. For a valid comparison of purified proteins, the authors need a mutant (preferably a single amino acid substitution) that abrogates nickase activity but has no effect on oligomerization or protein stability. Protein stability needs to be accurately assessed using biophysical methods. Since the authors are proposing a very unusual anti-CRISPR mechanisms that is only weakly supported by the other data presented, their biochemistry must be absolutely rigorous.**

*We agree with the editor’s comment. To address it in our revision, we now describe the discovery and characterization of two additional loss-of-nicking variants of AcrIIA22. One of these variants was discovered using an alanine-scanning strategy and varies from the original AcrIIA22 by just a single amino acid. The second variant is naturally occurring and varies from the original sequence by only two amino acids. We show that both variants are substantially impaired for nicking activity in vitro and for SpyCas9 inhibition in our plasmid protection assay in vivo. Yet, both variants show similar chromatography profiles to the wild-type protein, demonstrating that they fold correctly and have the same oligomeric state. As with our wild-type AcrIIA22 protein, we provide silver stained gels to show these mutant proteins are very pure. Unlike our truncation mutant in the initial submission, these two variants have similar oligomerization profiles to AcrIIA22. Yet, in all cases, in vitro nicking activity correlates with in vivo plasmid protection. These new biochemical data significantly bolster our claim that AcrIIA22 is a nickase and that nicking activity is required for SpyCas9 inhibition. These data can be seen in Figures 3, 6, and Supplemental Figure 10.*

**1d) The authors should also determine whether AcrIIA22 is co-purifying with nucleic acid. Removal of nucleic acid during purification may be important as other DNA-interacting proteins may be co-purifying with bound nucleic acid. Does AcrIIA22 bind to DNA?**

*This is an interesting suggestion, given AcrIIA22’s homology to other PC4-like homologs. However, even if AcrIIA22 were to co-purify with nucleic acids, a few lines of evidence argue against the possibility that co-purification of other DNA-binding proteins is responsible for the nicking activity we have observed. First, as highlighted in point 1b (above), our original and especially revised protein preparation schemes yield extremely pure AcrIIA22. Second, single or double amino acid variants that lose nicking activity also lose anti-CRISPR activity. Given the already broad scope of our manuscript, from functional metagenomics, to comparative genomes, structure, (now) mutational analysis, and biochemical mechanism, we respectfully believe these additional biochemical characterizations are outside the scope of this manuscript’s focus on AcrIIA22’s anti-SpyCas9 function.*

**2) The proposed mechanism of AcrIIA22 function is tenuous. SpyCas9 cuts linear DNA well in vitro as the authors show. The cutting of open circles by SpyCas9 in vitro is not reduced under all conditions. I find it hard to imagine how a presumably non-specific nickase would be able to provide just the right amount of nicking in vivo to inhibit SpyCas9 but not inhibit other processes that are crucial for the phage or plasmid. To convincingly argue about the mechanism of AcrIIA22, the authors would need some bona fide in vivo system where they would be able to correlate nick formation with anti-CRISPR activity. They have not done this even in the artificial in vivo assay in E. coli, and so this -if not experimentally proven- would need to be appropriately discussed and caveated.**

*To completely address the editor’s suggestion, we would have to test our model in as close to natural settings as possible. Unfortunately, the CAG-217 genus remains entirely uncultured in the lab. Moreover, no known plasmids or phages have been isolated from these bacteria. Instead, as detailed in point 1c (above), we now provide much stronger evidence of a strict correlation between nick formation in vitro with anti-CRISPR activity in vivo, using our heterologous system in Escherichia coli. Additionally, we have also substantially added to and improved our original analysis of SpyCas9’s substrate preference, as we detail in point 9 (below) and show in Figure 7. We also point out the caveats and limitations of a heterologous system as suggested.*

**3) The results do not convincingly support that the natural function of AcrIIA22 is as an anti-CRISPR. This protein is likely involved in recombination functioning as a single-stranded DNA-binding protein as suggested by its structure. Most phages encode recombination proteins. When over-expressed in E. coli, AcrIIA22 does inhibit SpyCas9, but it may inhibit many other DNA-interacting enzymes. It could be binding all over the plasmid DNA and inhibiting SpyCas9 that way. Given that AcrIIA22 homologs are mostly encoded in phages, it is discouraging that it does not inhibit phage replication particularly well even when overexpressed from a plasmid. To function within the context of a phage infection, high potency is required as the anti-CRISPR is initially present at very low concentration after genome injection. How do the authors consider that this may be working? Ideally, data would be provided to support a role during infection, but at a minimum they would need to discuss AcrIIA22 function in this context.**

*There are several points raised here by the editor. The first concern raised is that AcrIIA22 might be a general inhibitor of many other DNA-interacting enzymes. In new experiments, we show this is not the case. In revised Figure 3C, we provide new data that shows AcrIIA22 protects against Cas9 but not Cas12, which suggests it does not broadly inhibit DNA-interacting enzymes. The second point raised is that the level of inhibition observed by AcrIIA22 is too low to be considered a bona fide Acr. In this regard, we discuss several roles for weak Acr activity in nature. For instance, we highlight previous findings that phages can cooperate to bring about Cas9 inhibition cumulatively through sequential rounds of infection, even when a single round of infection is insufficient for (bona fide) Acr-mediated inhibition of Cas9. This dynamic can favor weak Acrs over strong ones, by suppressing ‘cheater’ phages in the population that lack Acrs. Also, many Acrs, including some Cas9 inhibitors, act via mechanisms that are most likely beneficial to integrated prophages, rather than lytic viral particles. When expressed from prophages, more time may be available for anti-CRISPRs to act and thus a wider array of mechanisms possible. In most of these cases, prophage-specific acrs co-localize with lytic-specific acr genes, as does acrIIA22 with acrIIA17. When discussing these data, we now describe previous work detailing these prophage Acrs. Finally, as suggested by the editor, we have now added significant discussion that speculates on potential roles for AcrIIA22 in CAG-217 phage and plasmid infection in our revision.*

~~~~~~~~~~~~~~~~~~~~~~~~~~~~~~~~~~~~~~~~~~~~~~~~~~~~~~~~~~~~~~~~~~~~~~~~~~~~~~~~~~~~~~~~

**Reviewer #1 (Evidence, reproducibility and clarity (Required)):**

**Forsberg et al. describe the characterization of a small 54-aa protein, encoded by hypervariable regions of clostridial bacteria and their prophages, that was selected from a metagenome library based on its capacity to completely protect a plasmid from being degraded by SpCas9. The authors call the protein an anti-CRISPR protein, AcrIIA22. However, characterization of the protein reveals a rather general mode of action. It does not, like many Acr proteins, directly interact with some of the key proteins of the CRISPR-Cas machinery. Its crystal structure reveals homology to PC4-like nucleic acid binding proteins such as the single-stranded binding protein of E.coli phage T5 that is involved in unwinding of dsDNA, and/or recombination. Analysis of the AcrIIA22 protein reveals it is has nickase activity that result in relaxation of supercoiled dsDNA. This activity appears to cause a substantial decrease in interference by SpCas9, protecting the plasmid from being cleaved. At the same time, it also caused slight toxicity as deduced from a 7% drop in growth rate. This is an interesting study, well executed and presented. There are some matters that should be addressed as indicated below.**

*We thank the reviewer for their positive remarks and helpful suggestions for improving our manuscript.*

****Major comments****

**4) As the authors admit, there is no direct interaction of AcrIIA22 with the type IIA Cas9 nuclease. Hence, the question is if this protein should be called an AcrIIA system, or even whether it should be classified as an anti-CRISPR protein. Given the indirect effect on Cas9 targeting, it is anticipated that the nickase activity will affect all enzymes that rely on a negatively supercoiled dsDNA configuration, possibly including non-CRISPR defence systems, but certainly including Type IE-IF Cascade/Cas3 systems (REF 32), Type IIA-C Cas9 systems (REFS 13-16). In addition, one would assume that also Type V Cas12 systems would be sensitive to the topology of their dsDNA target. So at best the name should be "general Acr protein".**

*We have followed the reviewer’s suggestion and have tested AcrIIA22 against additional CRISPR-Cas systems (revised Figure 3C). We chose type II and type V systems, as these are the only ones naturally occurring with its host CAG-217 genus. We found that AcrIIA22 can protect plasmids from Cas9, but not Cas12-based systems. Thus, we have elected to keep the type II nomenclature for AcrIIA22.*

**5) To make claims that the AcrIIA22 protein is more potent to protect MGEs against Type II Cas9 than against Type I Cascade, either this experiment should be done, or the claims (line 343-345) should be toned down.**

*We thank the reviewer for their suggestion. We have amended our language to make this point clearer. We now refer to published in vitro data that demonstrates Cas9 is more susceptible to supercoil state than type I Cascade.*

**6) A crystal structure of AcrIIA22 is provided, evidence is provided for metal-dependent nickase activity, and alignments have been made (to build a phylogenetic tree) of closely and distantly related sequences. Still, nothing is mentioned about conserved (metal-binding) residues that might constitute the catalytic site that allows for nicking. At least an in silico analysis should be included, and whenever possible an experimental verification of the prediction.**

*We agree with this comment. This point was raised by multiple reviewers and is discussed in response to point 1c. Briefly, we performed an alanine mutagenesis scan to identify putatively catalytic residues important for function, purified the most affected mutant, demonstrated impaired nicking activity despite no change in oligomeric effects, and provide a structural analysis of the putative metal-binding residues. These new data are presented in revised Figure 6 and revised Supplemental Figures 8 and 10.*

**7) Line 110 - Details should be provided on the genetic material of phage Mu (RNA/ssDNA/dsDNA, linear/circular), as basis for discussion why AcrIIA22 does not seem to protect it from Cas9 cleavage.**

*We have now added these details in our revised Discussion (see also response to point 3, above).*

****Minor comments****

**Line 55 - provide detail on 'novel mechanism' of AcrIIA11**

*We’ve updated our language to avoid discussing AcrIIA11’s mechanism all together. Though it exhibits novel features, full mechanistic clarity remains incomplete and is outside the scope of this manuscript.*

**Line 72 - what inducible promoter?**

*We apologize for the lack of clarity. We have updated language in our main text and methods to clarify that this refers to the arabinose-inducible promoter, pBAD.*

**Reviewer #1 (Significance (Required)):

This is an interesting study, well executed and presented. There are some matters that should be addressed as indicated above.

My expertise is Mol Microbiology, with focus on CRISPR-Cas biochemistry**

*We appreciate the reviewer’s affirming praise and thank them for their constructive criticisms. We hope they are satisfied by our new experiments to fully address their comments.*

~~~~~~~~~~~~~~~~~~~~~~~~~~~~~~~~~~~~~~~~~~~~~~~~~~~~~~~~~~~~~~~~~~~~~~~~~~~~~~~~~~~~~~~~

**Reviewer #2 (Evidence, reproducibility and clarity (Required)):**

**Anti-CRISPRs (Acrs) are small proteins harbored by phages that inactivate CRISPR-Cas systems and thereby enable tolerance of infection. Identifying Acrs and understanding their mechanism of action has proven to be important for both understanding host/phage interactions as well as controlling CRISPR-based biotechnologies, most notably, Cas9-mediated genome editing. The known Acrs are diverse in their amino acid sequence, protein structure, and mechanism of Cas inhibition. The authors recently described a genetic approach which identified a new Acr (AcrIIA11) by screening metagenomic libraries for inhibition of Cas9-mediated plasmid cleavage in E. coli (Forsberg et al. Elife 2019).**

**In this manuscript, the authors characterized a second metagenomic contig that was hit in the screen. They identified a single gene (AcrIIA22) from this contig which is responsible for inhibiting Cas9-mediated interference of the targeted plasmid and to a lesser extent, Mu phage. Using thorough bioinformatic analysis the authors detected several functional AcrIIA22 homologs in prophages and hypervariable islands within the Clostridial genus CAG-217. The authors purified the AcrIIA22 protein and determined its crystal structure, found that it forms a tetramer in solution, and identify residues required for oligomerization. Unlike most Acrs, this one did not exhibit detectable binding to its cognate Cas protein and did not inhibit Cas9 cleavage of linear DNA substrates. Instead, the authors used a structural homology search and plasmid mobility assays (both in vivo and in vitro) to show that AcrIIA22 possesses DNA nickase activity. They perform experiments on pre-nicked plasmid substrates that suggest AcrIIA22 inhibits Cas9 by removing the DNA supercoils that Cas9 requires for maximal activity.**

*We are grateful for the reviewer’s careful reading of our manuscript, their summary, and their constructive suggestions.*

****Major comments:****

**8) The in vivo experiments convincingly establish that AcrIIA22 is a member of a family of anti-CRISPR proteins that inhibit Cas9 in both anti-plasmid and anti-phage interference. The in vitro data demonstrating that AcrIIA22 is a nickase are compelling as well. The ∆2aa mutant data suggest that oligomerization is required for both nicking and Cas9 inhibition. I commend the authors on their thorough exploration of AcrIIA22 function and evolution.**

*We thank the reviewer for their very kind comments.*

**9) I have some reservations regarding the claim that AcrIIA22-nicked plasmids are resistant to Cas9 cleavage. The major piece of experimental evidence supporting this idea is presented in Figure 6C, where plasmid substrates were pre-nicked by either AcrIIA22 or another nickase, and then are tested as substrates in SpyCas9 DNA cleavage assays. The manuscript text states "SpyCas9 showed a clear preference for cleaving the supercoiled substrate versus the AcrIIA22-treated open-circle plasmid", but after looking at the figure (either on screen or a print-out) I had some difficulty agreeing with this assessment. This is largely because in the reactions presented, Cas9-mediated cleavage of the un-treated substrate (Fig 6C - lane 5) has not gone to completion: most of the supercoiled substrate remains uncleaved and there is a fairly weak linear cleavage product. Both of the pre-nicked substrates (lanes 4 and 6) also appear to have weak linear product bands. To my eye, there is very slightly more linear Cas9 cleavage product for the supercoiled substrate than there is for pre-nicked. The authors note in the Figure 6 legend that some of the Cas9 cleavage product is open-circular (a single strand cut). However, the untreated plasmid (lane 2) also contains open-circular DNA. Altogether it is difficult to tell whether the nicked plasmid is indeed any less sensitive to Cas9 than the supercoiled form. A few suggestions / points related to this:**

*We appreciate this suggestion and agree with the reviewer. For this purpose, we re-optimized this assay and have extensively re-worked the relevant figure panels (now Figures 7C-E). In addition to generating clearer gel images, we also provide triplicate measurements, time course data, and statistics to support our claims for SpyCas9 substrate preference.*

**10) Can the reactions be run for longer time periods to generate more linear Cas9 product? This should make the difference between the nicked and supercoiled substrates more apparent, if the nicked substrates are indeed resistant to cleavage.**

*This suggestion was very helpful. We have gratefully incorporated it into our revision.*

**11) The experiment shown in Fig S10B (same experiment as Fig 6C, different reaction conditions) seems to suggest that pre-nicked substrates are still susceptible to cleavage by Cas9. The authors state in the legend that "the reaction proceeded too quickly to detect SpyCas9's substrate preference", however the reaction is only at about 50% completion and the three substrates have been cleaved equally. Why shouldn't the interpretation be that Cas9 prefers neither substrate?**

*We agree with the reviewer’s criticism that this figure panel was confusing. For reasons we speculate about below, buffer choice seems important for detecting differences in cleavage rates for bulk Cas9 digestions. So, we initially included this panel in service of full transparency. However, in response to confusion from this reviewer and others, we have elected to remove these data from our revised manuscript. Now, we simply report the buffer conditions used for our cleavage reaction, as is typical for nearly all anti-CRISPR mechanistic studies. Nonetheless, we offer a detailed answer to this reviewer’s question below:*

*The reviewer’s interpretation of Figure S10B is accurate in isolation but is inconsistent with our other data and an extensive body of literature, which demonstrates Cas9 has improved activity on negatively supercoiled dsDNA substrates (e.g. Szczelkun, PNAS, 2014; Tsui, ACS Synth Bio, 2017; Ivanov, PNAS, 2020). We can reconcile this apparent discrepancy because Cas9 is a single-turnover enzyme in vitro (it remains bound to the DNA substrate after cleavage). Thus, differences in Cas9 reaction rates can be difficult to detect in vitro compared to more canonical reactions with conventional, multi-turnover enzymes (with multi-turnover enzymes, small differences in reaction rate are amplified across successive reaction rounds). Empirically, we found that buffer conditions were important for slowing Cas9 activity to reveal its substrate preference. Notably, the buffer we settled on (used in Figures 7C-E) is identical to cleavage buffers used in other anti-CRISPR mechanistic studies (e.g. Harrington, Cell, 2017).*

*In vivo, Cas9 acts as a multi-turnover enzyme because it is evicted from its DNA substrate by host proteins. Thus, changes to reaction rates can have multiplicative effects in vivo that are not detectable in vitro. As a corollary, detectable changes in vitro are likely to have outsized impacts on Cas9 cleavage rates in vivo. Moderate reductions to Cas9 cleavage kinetics can, in turn, cause major functional defects in target clearance. This is because Cas9 is engaged in a kinetic race with its target, so must eliminate a target faster than the target can replicate. We more extensively discuss this in our revision. For these reasons, we are confident that the moderate substrate preference that we demonstrate for Cas9 in vitro satisfactorily explains AcrIIA22’s phenotype in vivo.*

**12) The authors state that AcrIIA22 "capitalizes on SpyCas9's stringent requirement for negative supercoils to form a productive R-loop". Does SpyCas9 strictly require negative supercoiling for R-loop formation and DNA cleavage? SpyCas9 is capable of cleaving short linear DNA substrates, which should have minimal supercoiling. Previous single-molecule studies investigating Cas9's supercoiling dependence (references 13-16) seem to suggest that rather than a stringent topology requirement, Cas9 binding and cleavage is enhanced by negative supercoiling. Thus, perhaps a quantitative assessment of cleavage kinetics for each substrate would be revealing?**

*We agree with the reviewer’s comment. Although negative supercoiling is clearly important to Cas9 function, especially in vivo, it is not a strict requirement for activity in vitro, where it instead enhances function. We have edited our language to reflect this more nuanced perspective.*

**Reviewer #2 (Significance (Required)):**

**Anti-CRISPRs hold promise for enhanced control of Cas9-mediated genome editing. The discovery of a new Acr with a new mechanism of action could lead to tighter off-switches for Cas9, especially when used in combination with other known Acrs. Understanding the effects of DNA topology on Cas9 function will inform future technological uses of Cas9.**

*We thank the reviewer for their constructive suggestions and favorable comments.*

****Referees cross-commenting****

**13) I agree with Reviewer #1's major comments 1-2 regarding the expected general anti-nuclease property of AcrIIA22. Testing the inhibitory activity of AcrIIA22 against other CRISPR-Cas types would improve the study.**	*We have now performed these experiments, as we have detailed in response to reviewer #1.*

~~~~~~~~~~~~~~~~~~~~~~~~~~~~~~~~~~~~~~~~~~~~~~~~~~~~~~~~~~~~~~~~~~~~~~~~~~~~~~~~~~~~~~~~

**Reviewer #3 (Evidence, reproducibility and clarity (Required)):**

****Summary:**

Forsberg et al. describe a new anti-CRISPR protein AcrIIA22, which was identified in a previously reported screen for anti-CRISPR effectors. A genomics analysis revealed that the presence of AcrIIA22 is a feature shared by diverse hypervariable regions of clostridial genomes. Crystal structure of AcrIIA22 is presented with the main finding being the structural basis of its oligomerization. This information was used to design a deletion mutant which did not oligomerize and was unable to inhibit Cas9. The authors also show that in vitro plasmids pre-treated with AcrIIA22 are nicked and less susceptible to Cas9 cleavage. These results lead to a model in which AcrIIA22 nicks DNA to relieve its supercoiling required for Cas9 activity. This inhibits Cas9 activity which would constitute a novel mode of indirect anti-CRISPR action.**

*We thank the reviewer for their accurate summary our manuscript.*

****Major comments:****

**14a) The main concern about this work is the proposed nickase activity of AcrIIA22. PC4 proteins are known to bind nucleic acids but have not been shown to have enzymatic activities. The basis of the proposed enzymatic activity of AcrIIA22 is in fact not even discussed in the paper. The metal dependence of the nickase activity (Suppl. Fig 9) follows a pattern typical for many metal-dependent nucleases with Mg2+ supporting the activity and Ca2+ being inhibitory. This would imply that AcrIIA22 is a typical nuclease with a metal ion-binding active site. No such active site has been identified in PC4-related proteins and it is not described in the current work based on the AcrIIA22 structure itself. To support the main conclusions of the manuscript, the authors need to better document AcrIIA22 nuclease activity for example by relatively simple experiments with short synthetic DNA substrates (single- and double-stranded).**

*The academic editor and reviewer #1 raised similar concerns, that we have not sufficiently described the structural and functional basis for AcrIIA22’s nicking activity. We agree with all editors and reviewers that this was an important point to address. As we discuss in response to points 1c and 6, we now believe we have satisfactorily addressed these concerns.*

**14b) It is also very important to exclude the possibility that the nickase activity of purified AcrIIA22 comes from impurities in the protein preparation. DNase is a common activity in living cells. As a minimum, the quality of the protein needs to be shown in the Supplementary Information. The possible basis of the proposed enzymatic activity of PC4 needs to be thoroughly discussed, preferably based on the structural data.**

*We believe we have addressed this concern, as we detail in response to points 1b and 1c. We also provide a much more thorough structural examination of AcrIIA22, as we detail in response to point 16.*

**15) A key element of the proposed mechanism is lower activity of Cas9 on open circle form of plasmid DNA. It is concerning that under certain conditions (Supp. Fig 10B), supercoiled and and nicked plasmids are cleaved by Cas9 equally well. The authors argue that this was due to the high reaction rate. This needs to be confirmed by time-course experiments at lower temperature which should slow down the reaction.**

*As the reviewer requests, we now depict time course experiments in Figure 7E. We also discuss this supplemental figure in detail in response to point 11.*

**16) Multiple structures of PC4 proteins in dimeric state and with nucleic acid bound are available (for example PMID: 16415882). The dimer architecture is conserved between PC4 and PC4-like protein from phage T5, so it is likely a universal feature of these proteins. Figure 4 needs to show more clearly how the typical PC4 dimerization is similar and different from the dimer present in the asymmetric unit of AcrIIA22 structure (Fig. 4A) and the proposed oligomeric state of the protein (Fig. 4D). Would the delta2aa mutant disrupt this canonical dimerization? Is the reported PC4 DNA binding mode compatible with the oligomeric state of AcrIIA22 or do authors imply a novel mode of the interactions with nucleic acid (dashed arrow in Fig 4E)? It is also important to show the ability of AcrIIA22 and its deletion mutant to bind single-stranded DNA. This can be done by a relatively simple extension of the gel filtration experiment shown in Fig 4C with protein-DNA complexes. Preferably, if possible, these experiments should be done with an in-line MALS-based measurement of the molecular weight. This will provide much more accurate MWs determination compared with column calibration traces.**

*We thank the reviewer for requesting these additional structural comparisons as they significantly strengthen our manuscript. Figure 4D and Supplemental Figures S8C-E now show that the typical PC4-like dimer resembles the lower portion of our proposed tetramer. The predicted mode of AcrIIA22’s DNA binding is broadly compatible with reports from other PC4-like proteins, as we attempt to show in Figure 4D and Supplemental Figures 8B-E. The delta2aa mutant likely prevents the red-hued monomers from attaching to the blue-hued base in our proposed tetramer, as we now indicate in Figures 4C, 4F, and Supplemental Figure S8A.*

*The asymmetric unit of AcrIIA22 depicts a dimerization interface along the N-terminal β-strand of each monomer (Figure 4E). This is unlike most PC4 dimers and our proposed tetramer but, interestingly, this interface may permit additional AcrIIA22 monomers to bind the exposed N-terminal β-strands of the red-hued monomers in Figure 4C to form a higher-order oligomer. We find this interesting, in part, because the resulting filament-like structure would be reminiscent of the ssDNA-dependent oligomerization used by some PC4 homologs to unwind duplex DNA in an ATP-independent manner (Janowski, 2020). We do not speculate on this possibility, however, because the functional impacts of this oligomerization (both for AcrIIA22 and other PC4-like homologs) are unclear.*

*The detailed analysis of AcrIIA22’s interaction with synthetic ssDNA fragments would certainly be interesting, especially to determine which biochemical properties it shares with other PC4-like homologs. However, we believe these experiments are outside the scope of our manuscript’s focus on AcrIIA22’s anti-Cas9 function.*

**17) Samples of electron density maps are not shown in the manuscript and the detailed PDB validation report was not attached to this submission. However, based on Table 1 the structural work appears very solid with good dataset and structure statistics.**

*We apologize for the omission. We now include this detailed PDB validation report as part of our revised submission (Supplementary Dataset 7). We thank the reviewer for their kind comments.*

**18) For in vitro experiments the numbers of replicates are not reported and quantification graphs do now show error bars. Quantification of nickase activity of delta2aa variant is shown in Fig 5C. This is an important experiment which may support the notion that the reported activity does indeed come from AcrIIA22 and not from impurities in its preparation. Therefore, the gels used to generate these data need to be shown in Supplementary Information, similar to the experiments for which the quantification is shown in Fig 5C.**

*We agree with this comment. All experiments are now performed in triplicate, statistics are quantified, and representative gel images are shown for key experiments.*

****Minor comments:****

**19) Authors state the homology to PC4 suggested that AcrIIA22 is a nickase. However, the rationale for this is not clearly described. Otherwise, the presentation of the data is clear and previous work is properly referenced.**

*We agree with this comment about unclear rationale and apologize for it. We have now clarified this in our revision. Briefly, the homology to PC4-like proteins prompted us to look at AcrIIA22’s potential interaction with DNA and topology effects, but gave no initial indication of nicking activity. Instead, we empirically observed nicking activity when we began investigating AcrIIA22’s effect on a target plasmid.*

**20) The nature of the proposed mechanism and the results with Mu phage (weak protection by AcrIIA22) suggest that the presence of AcrIIA22 would create an advantage for circular dsDNA phages and only when their genome is present in supercoiled form. The authors may want to discuss this and the prevalence of circular dsDNA phages of Clostridium bacteria.**

*This is a good suggestion and we have gratefully incorporated these points into our discussion.*

**Reviewer #3 (Significance (Required)):**

**21) The main finding from the structural part is the basis of AcrIIA22 oligomerization, but it needs to be further analyzed as described above. The fact that AcrIIA22 is similar to PC4-like proteins was likely apparent from the protein sequence because a homology model of the protein could be built which was successfully used for solving the crystal structure by molecular replacement.**

*As we discuss in response to point 16, we have performed these additional structural analyses. We also note that, though the initial AcrIIA22 homology model did show similarity to PC4-like structures, we were not confident in this homology until the final structure was solved. This is because AcrIIA22 folds into a relatively simple 4-stranded β-sheet. Due to its simplicity, the homology model had, at best, moderate confidence scores and was topologically similar to many structures. These ambiguities were clarified only after detailed structural analyses, including examining potential oligomeric assemblages.*

**22) Overall, this is a potentially very interesting work describing a novel mode of indirect CRISPR inhibition by relieving of DNA supercoiling. This could be an important conceptual advance. CRISPR mechanisms are obviously a very timely and popular subject, so this work would be interesting for a wide audience interested in these mechanisms.**

*We thank the reviewer for their very positive assessment of our manuscript’s conceptual importance, broad appeal, and impact in the field.*

**Reviewer's expertise: nucleic acid enzymes, protein biochemistry, structural biology.
I cannot evaluate the genomics part of the manuscript very thoroughly.**
